# Timing and deciphering mitochondrial DNA macro-haplogroup R0 variability in Central Europe and Middle East

**DOI:** 10.1186/1471-2148-8-191

**Published:** 2008-07-04

**Authors:** Anita Brandstätter, Bettina Zimmermann, Janine Wagner, Tanja Göbel, Alexander W Röck, Antonio Salas, Angel Carracedo, Walther Parson

**Affiliations:** 1 Institute of Legal Medicine, Innsbruck Medical University, Innsbruck, Austria; 2 Division of Genetic Epidemiology, Department of Medical Genetics, Molecular and Clinical Pharmacology, Innsbruck Medical University, Innsbruck, Austria; 3 Research Center for Agriculture and Forestry Laimburg, Auer, Italy; 4 Institute of Legal Medicine, University of Cologne, Cologne, Germany; 5 Institute of Mathematics, University of Innsbruck, Innsbruck, Austria; 6 Institute of Legal Medicine, Genomic Medicine Group, CIBERER, University of Santiago de Compostela, Spain

## Abstract

**Background:**

Nearly half of the West Eurasian assemblage of human mitochondrial DNA (mtDNA) is fractioned into numerous sub-lineages of the predominant haplogroup (hg) R0. Several hypotheses have been proposed on the origin and the expansion times of some R0 sub-lineages, which were partially inconsistent with each other. Here we describe the phylogenetic structure and genetic variety of hg R0 in five European populations and one population from the Middle East.

**Results:**

Our analysis of 1,350 mtDNA haplotypes belonging to R0, including entire control region sequences and 45 single nucleotide polymorphisms from the coding region, revealed significant differences in the distribution of different sub-hgs even between geographically closely located regions. Estimates of coalescence times that were derived using diverse algorithmic approaches consistently affirmed that the major expansions of the different R0 hgs occurred in the terminal Pleistocene and early Holocene.

**Conclusion:**

Given an estimated coalescence time of the distinct lineages of 10 – 18 kya, the differences in the distributions could hint to either limited maternal gene flow after the Last Glacial Maximum due to the alpine nature of the regions involved or to a stochastic loss of diversity due to environmental events and/or disease episodes occurred at different times and in distinctive regions. Our comparison of two different ways of obtaining the timing of the most recent common ancestor confirms that the time of a sudden expansion can be adequately recovered from control region data with valid confidence intervals. For reliable estimates, both procedures should be applied in order to cross-check the results for validity and soundness.

## Background

According to the newest interpretation of C14 calibration data, and according to previous studies on human evolution, Europe was populated around 41–46 thousand years ago (kya) [1-3]. The main features of the post-glacial colonization of Europe was reliably reconstructed using parts of the human mitochondrial genome (mainly the hypervariable segment I; HVS-I [2-5]) or the entire mtDNA molecule [6-8].

In Europe, with the exception of U5 and V, which most likely arose *in situ*, all mtDNA hgs (H, I, J, K, T, U2e, U3, U4, X, and W) are most likely of Middle Eastern origin and were introduced by either the protocolonization 41–46 kya, by later arrivals in the late Paleolithic or more recent contacts [2,9,10].

Nearly half of the West Eurasian pool of human mtDNA lineages is composed of subclades of the predominant West Eurasian hg R0 [6,11-13]. R0, formerly known as pre-HV [14], is defined by substitutions at nucleotide positions (nps) 73 and 11719 relative to R [15,16]. Its frequency as a whole declines towards the East and South, but in the Near East, the Caucasus and Central Asia its frequency is still as high as 10–30% [6,13,17]. Until now, more than 20 sub-lineages of hg H, the predominant subclade of R0, which accounts for roughly 40% of West Eurasian mtDNAs, have been described [6,12,13,17] and the variance of their regional distributions has been discussed [4,6,13,17,18].

Previous studies have proposed that hg H originated in the Middle East ~30 – 25 kya, expanded into Europe in association with a second Paleolithic wave (25 – 20 kya) and was strongly involved in late-glacial expansions from ice-age refugia after the LGM (20 kya) [2,9]. For a few sub-hg of hg H, coalescence ages were determined using either entire mtDNA genomes [6] or parts of the mtDNA control region [17]. Hg H1, H3 and V share an estimated common origin in the terminal Pleistocene (16 – 11.5 kya), with major expansion in the early Holocene (~10 kya) [6]. Recent estimates on expansion times of selected H sub-hgs [17] are in conflict with the appraisals derived by [6], thus leaving the question on the reliability on the applied methods unanswered.

The objective of this study was to provide new information concerning the phylogenetic characteristics of macro-hg R0, as well as to determine spatial distribution patterns and coalescence ages of all its major sub-hgs.

## Results and Discussion

In a total of 3,569 samples from five European populations residing in Central and South-East Europe (Austria, Germany, Hungary, Macedonia and Romania) and one Middle East population (Dubai), we found 1,408 samples (~39%) to belong to hg R0 based on either control region or coding region analysis. Of these, 1,350 samples contained enough DNA to obtain a dataset of full haplotypes consisting of entire control region sequences and 45 SNPs from the coding region. These haplotypes are listed in the Additional file 1.

### Haplogroup frequencies

**Figure 1:**
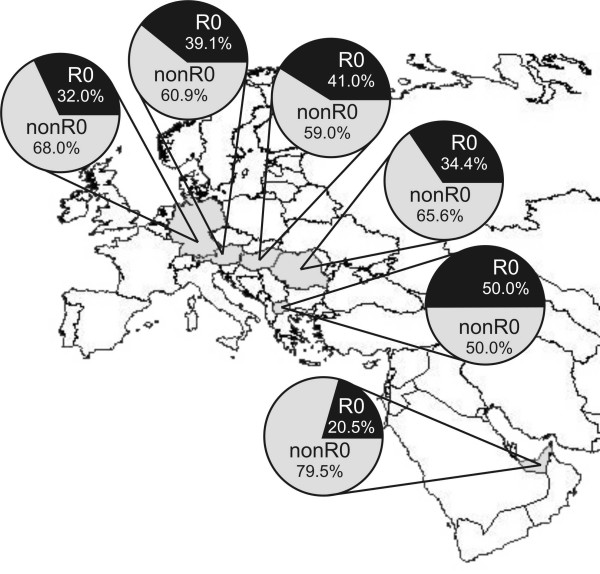
Distribution of mitochondrial hg R0 in population samples from Germany, Austria, Hungary, Romania, Macedonia and the Dubai (shown clockwise).

**Figure 2:**
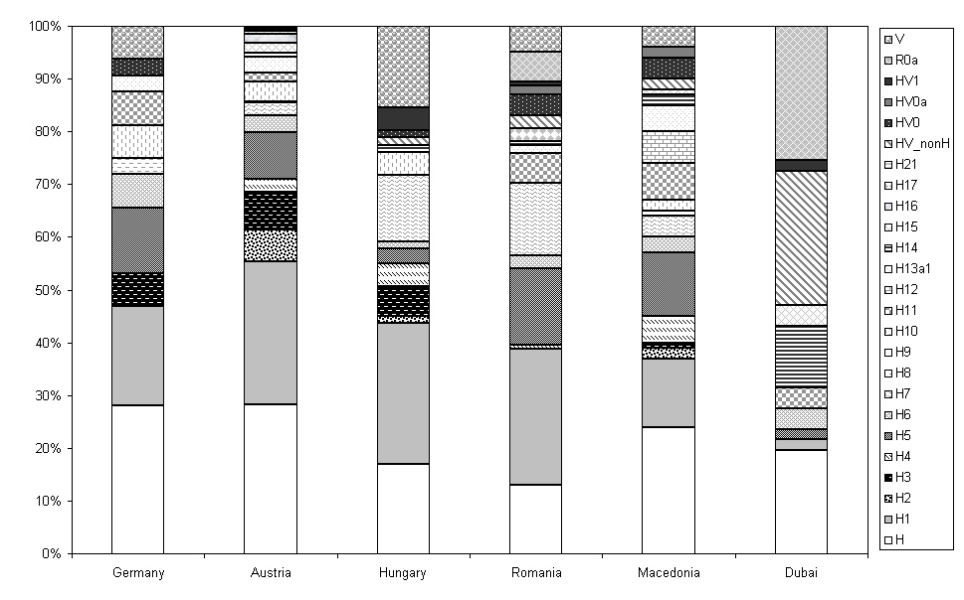
Relative frequencies of R0-hgs in different central European populations and one population from Dubai based on 45 mtDNA coding region SNPs and entire control region sequences.

The frequency of R0 in the different populations varied significantly (chi-square test; df = 5; p < 0.01) when taking all populations into account. Within Europe however, no significant differences were observed between populations (chi-square test; df = 4; p < 0.01). The location of the chosen populations and the respective proportion of R0 are shown in Figure 1. The five European populations from Austria, Germany, Hungary, Macedonia and Romania showed significant differences in the abundance of hgs H*, H2, H3, H7, H8, H9, H11, H12, HV0, HV1, R0a and V (chi-square test; df = 4; unadjusted p < 0.01). The prevalence of the remaining sub-hgs H1, H5, H6, H10, H13, H14, H15, H16, H17 and H21 was consistent across Europe (Table 1). Analyses of molecular variance (AMOVA; Tables 2, 3, 4) revealed significant statistical differences between most of the populations, except for the German population sample, which reflected well the typical European haplotype composition. A highly probable cause for the pronounced heterogeneity of the genetic substructure of R0 between the different populations (Figure 2) is genetic drift, which is particularly strong for less frequent haplogroups as most of R0 sub-haplogroups. Hg H was under-represented in the two Hungarian speaking populations Csángó and Székely from Romania, which could be explained by the described effects of genetic isolation especially in the Csángó population [19]. H2 and H3 exceeded the expected values in Austrians, while these hgs were not observed in the Romanians at all. H7, H11 and V occurred at unexpectedly high frequencies in the Hungarians from Romania and Macedonians. H12 was only found in the Macedonian population sample. Apart from Dubai, R0a was only observed in Romanian Hungarians. This observation reflects well the spatial frequency distribution of R0a, with the center of gravity in the Arabian Peninsula and only faint concurrencies in the south-eastern part of Europe [4].

**Table 1:** Frequency estimates of R0 sub-hgs found in six European populations (Austria, Germany, Hungary, Macedonia and Romania) and one Middle East population (Dubai).

Hg	Frequency	Percentage
H	346	25.6
H1	168	12.4
H1a	28	2.1
H1a1	12	0.9
H1b	53	3.9
H1c	46	3.4
H1c1	18	1.3
H1c2	7	0.5
H1f	3	0.2
H2a	9	0.7
H2a1	25	1.9
H2a2	24	1.8
H2a3	3	0.2
H3	69	5.1
H3b	6	0.4
H3c	1	0.1
H4	10	0.7
H4a	12	0.9
H4a1	1	0.1
H4a1a	10	0.7
H5	67	5.0
H5a	33	2.4
H5a1	24	1.8
H6	3	0.2
H6a	1	0.1
H6a1	37	2.7
H6b	1	0.1
H7	53	3.9
H8	2	0.2
H9	1	0.1
H10	44	3.3
H11	9	0.7
H11a	26	1.9
H12	6	0.4
H13a1	15	1.1
H13a1a	22	1.6
H14	16	1.2
H14a	2	0.2
H15	20	1.5
H16	18	1.3
H17	7	0.5
H21	1	0.1
HV_nonH	21	1.6
HV0	14	1.0
HV0a	4	0.3
HV1	5	0.4
R0a	20	1.5
V	27	2

Total	1350	100

**Table 2:** Design and results of AMOVA (Analysis of Molecular Variance)

Source of variation	d.f.	Sum of squares	Variance components	Percent of variation
Among populations	5	137.858	0.19128 Va	4.96
Within populations	1344	4923.636	3.66342 Vb	95.04
Total	1349	5061.494	3.85470	
Fixation index	FST:	0.04962		

**Table 3:** Population pairwise F_ST_s

	Hungary	Romania	Dubai	Austria	Macedonia	Germany
Hungary	0.000					
Romania	0.015	0.000				
Dubai	0.110	0.088	0.000			
Austria	0.041	0.030	0.185	0.000		
Macedonia	0.018	0.014	0.117	0.022	0.000	
Germany	0.011	0.006	0.107	0.007	-0.002	0.000

**Table 4:** F_ST _p-values.

	Hungary	Romania	Dubai	Austria	Macedonia	Germany
Hungary	*					
Romania	0.007	*				
Dubai	0.000	0.000	*			
Austria	0.000	0.000	0.000	*		
Macedonia	0.002	0.000	0.000	0.000	*	
Germany	0.106	0.158	0.000	0.078	0.549	*

### Phylogenetic topology of haplogroup R0

**Figure 3:**
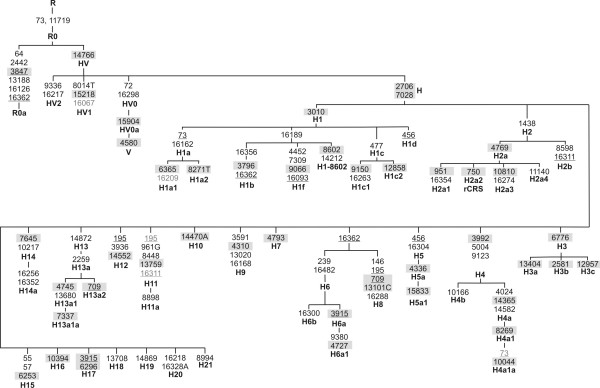
**Backbone of the phylogenetic tree of R0 subclades.** Mutations are transitions unless the base change is explicitly indicated. Deletions are indicated by a "d" following the deleted nucleotides. Underlining indicates recurrent mutations, whereas mutations in grey were added when observed with a high frequency in a certain branch. Mutations in grey boxes can be targeted with the SNP-multiplex published in [12].

R0 delineates from R* by the absence of nucleotide substitutions relative to the rCRS at positions 73 and 11719 and it can further be divided into two major clades: HV* is defined by the absence of transition C14766T, while R0a is defined by a set of variants at positions 64, 2442, 3847, 13188, 16166, and 16362 [20]. Figure 3 gives an overview of the phylogenetic backbone of R0 as described before and updated by [17] including new information from the present study.

The R0a sub-lineage is defined by the motif C64T T2442C T3847C C13188T T16126C T16362C [20]. This hg shows a particular instability in the neighborhood of position 64. All of our R0a samples showed C64T (which is part of the basal motif), half of them showed a T-insertion at position 60 (indicated in [20] as 57+T). This constitutes a sub-clade of R0a, R0a2; in [20] this sub-clade is named as (preHV)1b). All but one of the 20 R0a samples carried an additional transition T58C; which could correspond with (preHV)1a1 in [20]; this seems to constitute part of the motif defining a sub-branch of hg (preHV)1a (identified by transition 827 on top of the basal motif); here we rename (preHV)1a and (preHV)1a1 as R0a1 and R0a1a for consistency. Transition T58C appears also within R0a2 [20]. Conclusively, we found four different haplotypes between nps 58 and 64 within R0a: T58C-60.1T-C64T (10 individuals), 60.1T-C64T (1 individual), T58C-64T (7 individuals) and C64T alone (2 individuals). In [20] we find additional variation at this segment, for instance, their complete genome Ar222 (GenBank accession number DQ904241) carries a transversion T59A, while their sample Ar20 (DQ904235) carries transversion T58A.

Transition 16209C was found in eleven out of twelve H1a1-samples; therefore, this variant could be perfectly part of the motif of an H1a1 sub-branch. Position 73 has been traditionally used as a diagnostic position of hg R together with only one coding region transition, G11719A; this variant appears also quite often within hg H [21]. In addition, this variant appears for instance in complete mtDNA genomes within H1a (e.g. [6]), H3 [22], H4a (e.g. [23]), etc. Therefore, defining hg R by only A73G is imprecise. In our dataset, we observed for example the occurrence of A73G within H4a1. Within H7, a new sub-lineage, here identified as H7-709 seems to be characterized at least by the following array of variants G709A, T16172C and C16173T. This lineage seems to be geographically restricted to eastern Europe since it appears only in the Székely [19]; note that the control region motif T16172C C16173T appears also in the Kurdish ([24], but within hg I, as well as in other L and M hgs [25,26]. Within H10, another new sub-lineage carrying the additional control region markers T16093C and C16221T could be distinguished; the complete genome #32/H from [22] give support to this new branch. The transition C16114T could be part of the motif of a sister clade, as inferred from two complete genomes: Tor31 (AY738970; [6]) and the one from Family Tree DNA retrieved from GenBank with accession number EU073971. Transition T195C is probably part of the basal motif of H11, as identified in three complete genomes [6,27]. Our data also support the unnamed cluster identified by [17] characterized by the transition G7337A (and also T13326C). It can be tentatively said that H17 is additionally identified by position G16129A; however, so far there is only one complete genome available supporting this fact (AY495154; [23]).

### Demographic expansion and coalescence times

In order to estimate the time of the most recent common ancestor (TMRCA) of the different R0-subclades, two different approaches were applied: on the one hand, the shape of the distribution of the number of observed differences between pairs of entire control region sequences (16024–16569; 1–576) was used for timing expansions [28]. The mismatch distribution is usually multimodal in samples drawn from populations at demographic equilibrium, as it reflects the highly stochastic shape of gene trees, but it is usually unimodal in populations having passed through a recent demographic expansion [29,30] or through a range expansion with high levels of migration between neighbouring demes [31,32]. On the other hand, model-free rho statistics [33] were calculated on the basis of HVS-I (16024–16365) and entire control region sequences and used for estimating the coalescence time of the different subclades. Finally, the results were compared to each other and to so far published estimates (Table 5).

**Table 5:** Age estimates of R0 subclades.

Hg	N	τ	T ± ΔT (kya) (CR)^a^	ρ^b ^(CR)	T ± ΔT (kya) (CR)^c^	ρ^b ^(HVS-I)	T ± ΔT (kya) (HVS-I)^c^	T ± ΔT (kya) (HVS-I)^d^	T ± ΔT (kya) (Coding Region)^e^
**H**	346	3.3	**18.7 ± 2.4**	1.19	11.0 ± 1.5	0.86	**17.4 ± 4.1**	n.d.^f^	**18.4 ± 2.0**
**H1 (w/o H1a)**	295	2.6	**13.0 ± 0.9**	1.29	**12.0 ± 2.6**	0.56	**12.0 ± 3.2**	15.2 ± 5.1	**12.8 ± 2.4**
**H1a**	40	3.3	**18.7 ± 2.4**	2.03	**18.7 ± 6.7**	n.d.	n.d.	6.4 ± 3.6	n.d.
**H10**	44	2.3	13.1 ± 5.8	0.75	6.9 ± 3.0	0.59	11.9 ± 5.9	n.d.	n.d.
**H11**	35	1.9	10.8 ± 7.3	1.57	14.5 ± 4.1	1.03	20.7 ± 7.8	43.9 ± 18.5	n.d.
**H13a1**	37	1.9	**10.8 ± 1.9**	0.85	**8.0 ± 3.0**	0.49	**9.8 ± 5.7**	n.d.	n.d.
**H14**	18	4.5	**25.6 ± 5.4**	2.24	**20.7 ± 8.3**	1.18	**23.7 ± 8.6**	n.d.	n.d.
**H15**	20	1.8	**10.2 ± 3.4**	1.10	**10.2 ± 4.1**	0.10	2.0 ± 1.4	n.d.	n.d.
**H16**	18	2.8	16.0 ± 6.6	0.55	**5.1 ± 2.4**	0.39	**7.9 ± 4.9**	n.d.	n.d.
**H2a**	61	3.4	**19.3 ± 2.0**	1.89	**17.4 ± 7.1**	0.69	**13.9 ± 8.0**	12.3 ± 4.6	n.d.
**H3**	76	1.8	**10.2 ± 5.0**	1.11	**10.2 ± 2.8**	0.76	15.4 ± 6.0	16.0 ± 8.1	**10.3 ± 2.4**
**H4**	33	1.7	**9.7 ± 3.0**	0.79	**7.3 ± 3.5**	0.18	3.7 ± 2.1	11.5 ± 5.4	n.d.
**H5**	124	2.4	**13.6 ± 1.2**	0.98	9.0 ± 1.7	0.66	**13.3 ± 3.3**	**12.6 ± 4.4**	n.d.
**H6**	42	1.7	**9.7 ± 3.1**	0.81	**7.5 ± 2.0**	0.45	**9.1 ± 2.8**	3.4 ± 1.7	n.d.
**H7**	53	2.9	**16.5 ± 5.2**	1.09	10.1 ± 3.5	0.89	**17.9 ± 7.4**	**16.1 ± 7.4**	n.d.
**HV0**	45	3.0	17.0 ± 2.0	1.44	**13.4 ± 3.8**	0.47	9.4 ± 3.3	n.d.	**12.4 ± 2.5**^g^
**R0a**	20	6.1	35.0 ± 6.6	2.85	26.4 ± 9.1	1.50	30.3 ± 12.4	n.d.	n.d.

Concordant estimates (those showing overlapping intervals) are bold.

^a ^Estimate of the time of the most recent common ancestor of each cluster, using the demographic parameter τ inferred from the entire mtDNA control region (nps 16024–16569; 1–576)

^b ^Average number of base substitutions in the mtDNA entire CR (nps 16024–16569; 1–576) or HVS-I (nps 16024–16365) from the ancestral sequence type (rho statistics)

^c ^Estimate of the TMRCA of each cluster, using rho statistics inferred from entire CR or HVS-I sequences

^d ^Estimate of the TMRCA as published in [17] based on HVS-I sequences

^e ^Estimate of the TMRCA as published in [6] based on the mtDNA coding region (nps 577–16023) sequences

^f ^n.d.: not determined

^g ^Estimate was derived for hg V.

Interestingly, although different sequence ranges were used and different mathematical and conceptually different methods were applied, the estimates of the majority of subclades corresponded well with each other. In general, it can be said that most hg H sub-lineages show a strong signal for the beginning of the population expansion after the LGM, covering the Late Pleistocene and early Holocene.

**Figure 4:**
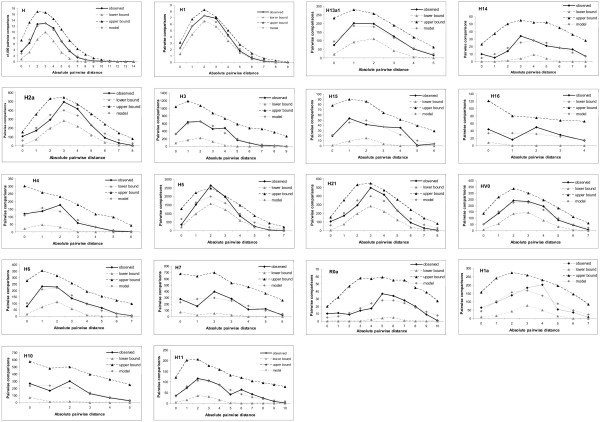
**Mismatch distributions within 18 different sub-hgs of R0 analyzed for the entire mitochondrial DNA control region.** Upper and lower limits represent 95% confidence intervals for the mismatch distributions. In all cases, an infinite-sites mutation model was used, assuming gamma distribution of mutation rates (α = 0.2).

The coalescence age for the entire hg H was estimated as 18.7 ± 2.4 thousand years ago (kya) using the parameter τ from the MMD of the entire CR, and 17.4 ± 4.1 kya using rho statistics based on HVS-I. Both estimates are in good concordance with each other and with the estimate from [6] (18.4 ± 2.0 kya). The expected and the observed MMD for hg H were found to be in good agreement (Figure 4), with an SSD *P *value of 0.312. SSD *P *values > 0.05 hint to the adequacy of the sudden expansion model, thus making the observed mismatch distributions compatible with the estimated parameters [28]. Despite an apparent lack of goodness-of-fit of some distributions (Figure 4), the adequacy of the sudden expansion model could only be rejected for hg H5 (SSD *P *value = 0.006). Interestingly, the rho estimate derived from entire CR sequences was low (11.0 ± 1.5 kya).

The near eastern hg R0a, which is geographically centred in the midst of the Arabian Peninsula [4], was estimated to have expanded between 26.4 ± 9.1 kya (rho statistics from the CR) and 35.0 ± 6.6 kya (MMD); the estimate derived from HVS-I lay in between (30.3 ± 12.4 kya).

The oldest sublineage of hg H was found to be H14, with highly concordant estimates for the TMRCA of 25.6 ± 5.4 kya (MMD) respectively 23.7 ± 8.6 kya (rho statistics). As in hg H, the estimate derived from CR rho statistics was lowest (20.6 ± 8.3 kya).

Consistent estimates for the TMRCA using both computational methods were obtained for most of the hgs (e.g. H, H1, H1a, H5, H6, H7, H13a1, H14, H15, H16) (Table 5). However, apart from H1 and H13a1, where our three estimates (MMD, rho statistics from entire CR and from HVS-I sequences) were highly consistent with each other, matching estimates were derived by comparing the MMD estimate with rho statistics from either CR or HVS-I sequences. In hgs H11 and HV0 the different approached used yielded very different mean values but associated with very large and overlapping confidence intervals.

Published estimates for the coalescence time of hg H3 lay at 16.0 ± 8.1 kya [17] or 10.3 ± 2.4 kya [6]. Our estimates were 10.2 ± 5.0 kya (MMD), 10.2 ± 2.8 kya (rho CR) and 16.7 ± 6.1 kya (rho HVS-I). Thus, both our values (MMD and rho) derived from entire control region sequences fit very well with the estimate from coding region sequences, while our rho estimate from HVS-I sequences fits well with the Roostalu's et al. HVS-I appraisal. It seems that HVS-I sequences in hg H3 do not correspond to the molecular clock calibration from the Eurasian tree, in a sense that mutations in HVS-I occur at a higher rate in H3 compared to phylogenetically closely related lineages.

Most recently, molecular dating has been questioned fundamentally, and the calibration of the molecular clock of the mtDNA control region as basic requirement for timing the most recent common ancestor has led to an ongoing discussion (for a review see [34,35]). However, occasional concerns that the molecular clock might be elusive and not tick regularly for human mtDNA were widely eliminated [35]. A coding-region rate estimate of 5140 years per substitution [36] corresponds well with estimates for the HVS-I rate [33] and with a rate of one control-region mutation per 9250 years for the Eurasian tree [35] and can be used to estimate the coalescence time of various haplogroups of the mtDNA phylogeny. A straight forward way to estimate the age of a particular "root" haplotype, given the mutation rate, is to consider all available descendant individual sequences and take the arithmetic mean over all distances to the root haplotype. This method is referred to as "rho" estimation [33] and can be performed using the freely available software package Network.

One of the disadvantages of the simple rho method is the conversion of DNA-data into files that can be handled with the network program. Without some in-house executables that assist the conversion, this would be an elaborate and error-prone process. On the other hand, network offers the calculation of the standard error of rho, which would be very complicated if calculated by hand.

The estimation of the demographic parameter τ from the mismatch distributions between pairs of sequences can be performed with ARLEQUIN, which seems to be more user-friendly regarding data import. One of the disadvantages of this method is that it does not account for the rate heterogeneity of the different mutations, and that it needs calibration against an archaeological or historical record.

The observed differences between the two approaches might thus rely on the slightly imprecise estimate of the mtDNA control region's molecular clock affecting the rho estimate and on the disregard of variation in site-specific mutation rates affecting the MMD estimation.

It seems that the rho statistics derived from entire CR-sequences tends to underestimate the TMRCA, while the rho statistics derived from HVS-I sequences tends to predate the TMRCA. This is most likely due to the calibration of the molecular clock of the different DNA stretches of the mitochondrial genome, which need further fine-tuning. Under this perspective, it seems advisable to follow both approaches for timing the coalescence age of phylogenetic clusters, and to put the estimates into a geological-historical context. In general, more complete genome information is desirable in order to get more accurate estimates.

### Genetic variability within R0-subhaplogroups

Hg H1 accounted for 12.4% of the samples and showed a high diversity in terms of different lineages (Additional file 2; mean number of pairwise differences: 4.92). Reticulations within H1 were mainly caused by mutational hotspots in the control region that emerged in its different sub-hgs, such as variation at 16129, 16189, etc.

Hg H2a represented 4.6% of the samples and displayed less genetic variation (Additional file 3; mean number of pairwise differences: 4.62). The control region marker 456 identifies hg H1d [17] and H5, but also popped up on an H2a2 background and led to one reticulation in the H2a network. This transition is however slightly unstable since it appears frequently in other non-H hg backgrounds, such as hg K [37].

Hg H3 was assigned to 5.6% of samples and was characterized by several clusters of identical profiles, thus implying little genetic differentiation (Additional file 4; mean number of pairwise differences: 3.06). The network of Additional file 4 shows just two reticulations provoked by two fast control region variants, T152C and T16311C.

Hg H4 corresponded to 2.5% of the samples, and showed very little genetic variation (Additional file 5; mean number of pairwise differences: 3.12). As in hg H3, mutations at position 16311 were responsible for network reticulations.

Hg H5 was realized in 9.2% of samples and revealed an enormous amount of lineage diversity (Additional file 6; mean number of pairwise differences: 4.51), especially when compared to the putatively same-aged Hg H3. Again, some fast positions are responsible for a number of reticulations, 146, 152, etc.

Hg H6 appeared in 3.1% of the samples and in relation to its relatively recent arrival in Europe (~9.7 kya), it exhibited pronounced genetic diversification (Additional file 7; mean number of pairwise differences: 2.97).

Hg H7 rendered for 3.9% of samples and demonstrated perfect tree-like evolution of lineages (Additional file 8; mean number of pairwise differences: 2.61).

Hgs H8, H9, H12, H17 and H21 could be differentiated in less than 0.5% of samples each. Due to small sample sizes, corresponding networks could only be constructed for H8 (Additional file 9) and H17 (Additional file 10).

Hg H10 was determined in 3.3% of the sequences but manifested hardly any genetic heterogeneity (Additional file 11; mean number of pairwise differences: 1.70).

Hg H11 encompassed 2.6% of the profiles and was estimated to have arisen 43.9 kya [17]. With respect to its frequency, its genetic distinction (Additional file 12; mean number of pairwise differences: 4.67) and our TMRCA estimations, the age estimate of [17] seems overstated.

Hg H13a1 comprised 2.7% of samples and similar to hg H7, the profiles grouped in big clusters mirroring a perfect tree-like pattern of evolution (Additional file 13; mean number of pairwise differences: 2.60).

Hg H14 and H16 covered 1.3% of sequences each, but the network of H14 (Additional file 14; mean number of pairwise differences: 5.84) indicated higher genetic diverseness than the network of H16 (Additional file 15; mean number of pairwise differences: 1.80).

Hg H15 embraced 1.5% of samples and was characterized by an array of uncommon mutations that led to a reticulation-free structure of the network (Additional file 16; mean number of pairwise differences: 4.91).

Hg H* (without the above described sub-hgs) was found in 25.6% of samples and showed an amazingly high grade of diversification (Additional file 17; mean number of pairwise differences: 4.13).

Hg R0a spanned only 1.5% of samples, but the corresponding network suggest an old age of the hg, with a complete tree-like structure (Additional file 18; mean number of pairwise differences: 5.85).

Hgs V, HV* (without H), HV1, HV0 and HV0a together comprehended 5.3% of the study individuals and map onto a straightforward phylogenetic network, with reticulations mainly caused by mutations on position 72 (Additional file 19).

## Conclusion

Our analysis of 1,350 mtDNA haplotypes belonging to R0, the most common hg-cluster in West Eurasia, revealed significant differences in the distribution of different sub-hgs even between geographically closely located regions. Given an estimated coalescence time of the distinct lineages of 10 – 18 kya, the differences in the distributions could hint to either limited maternal gene flow after the LGM due to the alpine nature of the regions involved, or to a stochastic loss of diversity due to environmental events and/or disease episodes occurred at different times and in distinctive regions (e.g. Black Plague between 1347–1666), or most likely, to genetic drift, particularly strong for less frequent haplogroups as most of R0 sub-haplogroups characterized here.

Our comparison of two different ways of obtaining the timing of the TMRCA confirms that the time of a sudden expansion (τ) can be adequately recovered from control region data with valid confidence intervals. The observed differences between the two approaches might rely on the slightly imprecise estimate of the mtDNA control region's molecular clock affecting the rho estimate and on the disregard of variation in site-specific mutation rates affecting the MMD estimation. So for reliable estimates, both procedures should be applied in order to cross-check the results for validity and soundness.

## Methods

### DNA samples

A total of 1,350 samples that belong to the hg cluster R0 were selected from five West Eurasian populations and one population from the Middle East, corresponding to a total of 3,569 samples. The majority of samples came from Austria (nr. of total samples: 2,487; nr. of R0-samples: 973) [12,38], the donors of the remaining samples originated in Germany (nr. of total samples: 100; nr. of R0-samples: 31) [39], Hungary (nr. of total samples: 173; nr. of R0-samples: 71) [40], Macedonia (nr. of total samples: 200; nr. of R0-samples: 100) [41], Romania (nr. of total samples: 360; nr. of R0-samples: 124) [19] and Dubai (nr. of total samples: 249; nr. of R0-samples: 51) [42].

### Genotyping

All samples were sequenced for the entire mtDNA control region (positions 16024–16569; 1–576) as described in [38] and screened for hg R0-specific single nucleotide polymorphisms within the mtDNA coding region as described in [12]. 847 Austrian samples had already been typed for the 45 coding region SNPs before [12] and were sequenced for the entire control region for the present study. Of the remaining 503 samples the control region sequences had been generated before [19,38-42] and the coding region SNPs were additionally typed for the present study.

### Phylogenetic reconstructions

Phylogenetic networks were constructed with Network.exe [43] applying the median joining algorithm [44] with the parameter epsilon set to 0. Polymorphisms within the control region were divided into five classes roughly according to their presumptive mutation rate [35]. Superfast positions (152, 16519, insertions at 573) were weighted by one, fast positions (16093 and the AC-repeat around 523–524) by three and speedy positions (93, 150, 151, 182, 183, 194, 195, 198, 199, 200, 204, 207, 228, 499, 513, 16051, 16069, 16111, 16124, 16126, 16129, 16145, 16148, 16153, 16163, 16166, 16167, 16168, 16169, 16172, 16173, 16184, 16186, 16187, 16189, 16192, 16209, 16214, 16218, 16223, 16224, 16230, 16234, 16243, 16245, 16248, 16249, 16256, 16260, 16261, 16264, 16266, 16270, 16271, 16278, 16287, 16290, 16292, 16294, 16295, 16298, 16300, 16309, 16311, 16316, 16319, 16320, 16325, 16327, 16344, 16355, 16360, 16362, 16390) by six. Transversions at the following positions were weighted by 15: 95, 185, 189, 16114, 16147, 16176, 16188, 16206, 16207, 16257, 16265, 16266, 16286, 16293 and 16318. The remaining polymorphisms within the control region were assigned weight 10. All coding region SNPs were assigned weight 25. Length heteroplasmic C-insertions at 309 and 315 were weighted by zero.

### Population genetic analysis

ARLEQUIN (Version 2.0; [45]) was used for the calculation of haplotype frequencies and analysis of molecular variance (AMOVA). Permutation tests (1,000 replicates) were used to evaluate the significance of F_ST _inter-population genetic distances. For all population genetic analyses, length heteroplasmic C-insertions were not taken into consideration.

### Demographic expansion and coalescence ages

The mismatch distributions (MMDs) within hgs (based on entire control region sequences) were calculated as the distribution of the observed number of differences between pairs of haplotypes with ARLEQUIN. The age of the demographic expansion, i.e. the demographic parameter τ, was estimated from the mismatch distributions of the different R0 subclades using a generalized nonlinear least-square approach [28]. The demographic parameter τ was calculated by minimizing the sum of square deviations (SSD) between the observed mismatch distribution and its expectation under an infinite-sites model. Approximate confidence intervals for τ were obtained by parametric bootstrapping with 1000 replicates. In order to test the validity of the sudden expansion model, SSD was complementarily used as a test statistic. The conversion of the expansion time τ into years since a population entered a demographic expansion phase was performed according to the equation τ = 2 ut, where u is the total mutation rate per generation per gene and t is the number of generations passed since the demographic expansion. Assuming a (matrilineal) generation time of 25 years, and dating the age of hg H at 18,400 years [6], u is estimated as 0.0022 in correlation with a value for τ of 3.3 (Table 5).

As a complementary model-free approach to date the most recent common ancestor of R0 sub-clades, median-joining networks of hypervariable segment I (HVS-I) sequences (16024–16365) and of entire control region sequences were constructed with the parameters as described above. The rho (ρ) statistics were calculated as the average number of mutations from the nodes to the root of the sub-hg networks and scaled by the mutation rate for HVS-I, where one transitional step was taken equal to 20,180 years [33], or by the rate of one control region mutation per 9,250 years [35]. Standard deviations for age estimates were calculated as in [46].

## Authors' contributions

AB carried out the phylogenetic, statistical and mathematical computations, and drafted the manuscript. BZ, JW and TG carried out the SNP typing and CR sequencing. AWR participated in the phylogenetic and mathematical computations. AS and AC helped to draft the manuscript. WP conceived of the study, and participated in its design and coordination and helped to draft the manuscript. All authors read and approved the final manuscript.

## Supplementary Material

Additional file 1 List of all 1350 samples with their hg affiliations, the state of origin, coding region SNPs and control region polymorphisms. The exact geographical origin was as follows: HUN: Ashkenazi Jews from Budapest; ROU: Hungarian speaking populations from Transylvania; ARE: a population sample from Dubai; AUT: a population sample from Innsbruck; MKD: a population sample from Macedonia; DEU: a population sample from the city of Ulm. Click here for file

Additional file 2 Median joining network of hg H1. Node sizes are proportional to haplotype frequencies. Variable positions are indicated along links that connect haplotypes. Nucleotide changes are specified only in the case of transversions. Polymorphisms in bold correspond to coding region sites. Italic polymorphisms are those of the central haplotype and are found in all haplotypes of the respective network if not indicated otherwise. Click here for file

Additional file 3 Median joining network of hg H2a. please see Additional file 2. Click here for file

Additional file 4 Median joining network of hg H3. please see Additional file 2. Click here for file

Additional file 5 Median joining network of hg H4. please see Additional file 2. Click here for file

Additional file 6 Median joining network of hg H5. please see Additional file 2. Click here for file

Additional file 7 Median joining network of hg H6. please see Additional file 2. Click here for file

Additional file 8 Median joining network of hg H7. please see Additional file 2. Click here for file

Additional file 9 Median joining network of hg H8. please see Additional file 2. Click here for file

Additional file 10 Median joining network of hg H17. please see Additional file 2. Click here for file

Additional file 11 Median joining network of hg H10. please see Additional file 2. Click here for file

Additional file 12 Median joining network of hg H11. please see Additional file 2. Click here for file

Additional file 13 Median joining network of hg H13a1. please see Additional file 2. Click here for file

Additional file 14 Median joining network of hg H14. please see Additional file 2. Click here for file

Additional file 15 Median joining network of hg H16. please see Additional file 2. Click here for file

Additional file 16 Median joining network of hg H15. please see Additional file 2. Click here for file

Additional file 17 Median joining network of hg H*. please see Additional file 2. Click here for file

Additional file 18 Median joining network of hg R0a. please see Additional file 2. Click here for file

Additional file 19 Median joining network of hg HV*. please see Additional file 2. Click here for file
