# Supplementary material for: Timing and deciphering mitochondrial DNA macro-haplogroup R0 variability in Central Europe and Middle East
Source: BMC Evol Biol. 2008 Jul 4;8:191. doi: 10.1186/1471-2148-8-191 (PMC2491632; doi:10.1186/1471-2148-8-191)
Supplement: Additional file 1 — List of all 1350 samples with their hg affiliations, the state of origin, coding region SNPs and control region polymorphisms. The exact geographical origin was as follows: HUN: Ashkenazi Jews from Budapest; ROU: Hungarian speaking populations from Transylvania; ARE: a population sample from Dubai; AUT: a population sample from Innsbruck; MKD: a population sample from Macedonia; DEU: a population sample from the city of Ulm. [file 1471-2148-8-191-S1.pdf]

HG	State	Sample	Polymorphisms
H	HUN	As1C10	750G-4769G-16519C-263G-309.1C-315.1C
H	HUN	As1C3	750G-4769G-709A-15218G-16148T-16256T-16319A-189G-193G-249G-263G-309.1C-315.1C
H	HUN	As1D1	750G-4769G-16111A-16188T-16519C-263G-309.1C-315.1C-417A
H	HUN	As1E2	750G-4769G-16519C-263G-309.1C-309.2C-315.1C
H	HUN	As1E5	750G-4769G-262T-263G-309.1C-315.1C
H	HUN	As1H1	750G-4769G-16129A-16519C-146C-263G-315.1C
H	HUN	As2A10	750G-4769G-16129A-16519C-263G-309.1C-309.2C-315.1C
H	HUN	As2B10	750G-4769G-16390A-16519C-263G-309.1C-309.2C-315.1C
H	HUN	As2C4	750G-4769G-16213A-16216G-263G-315.1C-456T
H	HUN	As2C8	750G-4769G-16129A-16519C-146C-263G-315.1C
H	HUN	As2D11	750G-4769G-16213A-263G-315.1C-456T
H	HUN	As2D5	750G-4769G-262T-263G-309.1C-315.1C
H	ROU	CS2B7	750G-4769G-16519C-152C-263G-315.1C
H	ROU	CS2C1	750G-4769G-16184T-16519C-263G-309.1C-309.2C-315.1C
H	ROU	CS2E12	750G-4769G-16189C-16193.1C-16519C-153G-204C-263G-309.1C-309.2C-315.1C
H	ROU	CS2H4	750G-4769G-16293G-16519C-150T-263G-309.1C-315.1C
H	ARE	DB1E7	750G-4769G-16145A-263G-309.1C-315.1C
H	ARE	DB1F4	750G-4769G-16243C-16311C-16519C-204C-207R-263G-309.1C-315.1C
H	ARE	DB1H10	750G-4769G-16209C-152C-263G-309.1C-309.2C-315.1C-524.1A-524.2C
H	ARE	DB2B1	750G-4769G-16192T-16201T-263G-309.1C-315.1C
H	ARE	DB2E2	750G-4769G-709A-16519C-153G-200G-263G-309.1C-315.1C
H	ARE	DB2G4	750G-4769G-16192T-16201T-263G-309.1C-315.1C
H	ARE	DB3A6	750G-4769G-16192T-16201T-152C-263G-309.1C-315.1C
H	ARE	DB3C4	750G-4769G-709A-16290T-16519C-131C-263G-309.1C-309.2C-315.1C
H	ARE	DB3D4	750G-4769G-16192T-16201T-152C-263G-309.1C-315.1C
H	ARE	DB3G9	750G-4769G-16278T-143A-152Y-263G-308.1T-315.1C
H	AUT	f1A5	750G-4769G-16519C-235G-263G-309.1C-309.2C-315.1C
H	AUT	f1B6	750G-4769G-16248T-16519C-73G-187T-263G-315.1C-523DEL-524DEL
H	AUT	f1D1	750G-4769G-16183C-16189C-16519C-263G-309.1C-309.2C-315.1C
H	AUT	f1E2	750G-4769G-16291T-16519C-263G-315.1C
H	AUT	f1F1	750G-4769G-16234T-16293G-16519C-263G-315.1C-315.2C
H	AUT	f1H3	750G-4769G-16311C-16519C-263G-309.1C-315.1C
H	AUT	f1H5	750G-4769G-16266T-16311C-16399G-16519C-263G-315.1C-523DEL-524DEL
H	AUT	f2B1	750G-4769G-16189C-16519C-152C-263G-309.1C-315.1C-385G
H	AUT	f2D3	750G-4769G-16072T-16245T-16311C-16519C-263G-315.1C-523DEL-524DEL
H	AUT	f3A6	750G-4769G-16189C-16519C-152C-263G-309.1C-309.2C-315.1C-385G
H	AUT	f3C2	750G-4769G-16209C-16519C-263G-315.1C
H	AUT	f3C3	750G-4769G-16183C-16189C-16519C-146C-263G-309.1C-309.2C-315.1C

H	AUT	f3F3	750G-4769G-16093Y-16519C-263G-309.1C-315.1C-573.1C-573.2C-573.3C-573.4C-573.5C-573.6C
H	AUT	hf1A4	750G-4769G-16209C-16311C-73G-93G-152C-263G-309.1C-315.1C-523DEL-524DEL
H	AUT	hf1A6	750G-4769G-16266T-16311C-16399G-16519C-263G-315.1C-523DEL-524DEL
H	AUT	hf1A7	750G-4769G-16189C-16519C-152C-263G-309.1C-315.1C-385G
H	AUT	hf1B2	750G-4769G-16189C-16193.1C-16193.2C-16519C-263G-309.1C-309.2C-315.1C
H	AUT	hf1B3	750G-4769G-3915A-16256Y-16519C-263G-315.1C
H	AUT	hf1C3	750G-4769G-16129A-16316G-16519C-263G-315.1C
H	AUT	hf1C5	750G-4769G-16189C-16192T-16519C-263G-309.1C-309.2C-315.1C
H	AUT	hf1C7	750G-4769G-16183C-16189C-16193.1C-16519C-199C-263G-309.1C-309.2C-315.1C
H	AUT	hf1D1	750G-4769G-16519C-263G-309.1C-315.1C
H	AUT	hf1D5	750G-4769G-16519C-263G-315.1C
H	AUT	hf1D7	750G-4769G-16519C-263G-309.1C-315.1C
H	AUT	hf1E10	750G-4769G-16327T-263G-309.1C-315.1C
H	AUT	hf1E6	750G-4769G-16519C-263G-315.1C
H	AUT	hf1E8	750G-4769G-16183C-16189C-16193.1C-16519C-263G-309.1C-315.1C
H	AUT	hf1F1	750G-4769G-16183C-16189C-16193.1C-16519C-199C-263G-309.1C-309.2C-315.1C
H	AUT	hf1F9	750G-4769G-16519C-152C-263G-315.1C
H	AUT	hf1G11	750G-4769G-16266T-16311C-16399G-16519C-263G-315.1C-523DEL-524DEL
H	AUT	hf1G2	750G-4769G-16266T-16311C-16399G-16519C-263G-315.1C-523DEL-524DEL
H	AUT	hf1G5	750G-4769G-16223T-16519C-263G-315.1C
H	AUT	hf1H1	750G-4769G-16234T-16293G-16519C-263G-315.1C-315.2C-315.3C
H	AUT	hf1H11	750G-4769G-16189C-16192T-16234Y-16519C-263G-315.1C
H	AUT	hf1H12	750G-4769G-16299G-16519C-146C-263G-309.1C-309.2C-315.1C
H	AUT	hf1H2	750G-4769G-16519C-195C-263G-315.1C-523DEL-524DEL
H	AUT	hf1H6	750G-4769G-263G-309.1C-315.1C
H	AUT	hf1H9	750G-4769G-16266T-16311C-16399G-16519C-263G-315.1C-523DEL-524DEL
H	AUT	hf2A1	750G-4769G-16519C-263G-309.1C-315.1C
H	AUT	hf2A4	750G-4769G-16266T-16311C-16399G-16519C-263G-315.1C-523DEL-524DEL
H	AUT	hf2A8	750G-4769G-16192T-16519C-263G-315.1C-315.2C-373G
H	AUT	hf2B2	750G-4769G-16519C-263G-309.1C-315.1C
H	AUT	hf2B9	750G-4769G-16180G-16254R-16519C-143A-152C-263G-315.1C
H	AUT	hf2C11	750G-4769G-16519C-235G-263G-309.1C-309.2C-315.1C
H	AUT	hf2C3	750G-4769G-16266T-16311C-16354Y-16399G-16519C-263G-315.1C-523DEL-524DEL
H	AUT	hf2C6	750G-4769G-16111T-16218T-16519C-93G-235G-263G-309.1C-315.1C
H	AUT	hf2D10	750G-4769G-16170G-16390A-16519C-263G-309.1C-315.1C
H	AUT	hf2D3	750G-4769G-16519C-263G-315.1C
H	AUT	hf2D5	750G-4769G-16176T-16519C-263G-309.1C-315.1C
H	AUT	hf2D6	750G-4769G-16183C-16189C-16193.1C-16519C-263G-309.1C-309.2C-315.1C
H	AUT	hf2E10	750G-4769G-16519C-263G-315.1C

H	AUT	hf2E11	750G-4769G-16192T-16519C-263G-315.1C-315.2C-373G
H	AUT	hf2E7	750G-4769G-16261T-16286T-262T-263G-309.1C-315.1C
H	AUT	hf2E8	750G-4769G-16519C-72G-263G-315.1C
H	AUT	hf2E9	750G-4769G-16519C-152C-200G-263G-315.1C-523DEL-524DEL
H	AUT	hf2F3	750G-4769G-16519C-146C-152C-263G-315.1C
H	AUT	hf2F4	750G-4769G-16189C-16192T-16519C-263G-315.1C
H	AUT	hf2F5	750G-4769G-16519C-263G-309.1C-315.1C
H	AUT	hf2G1	750G-4769G-16189C-16192T-16519C-263G-315.1C
H	AUT	hf2G2	750G-4769G-16519C-263G-315.1C
H	AUT	hf2G3	750G-4769G-16519C-263G-309.1C-315.1C
H	AUT	hf2G9	750G-4769G-16192T-16519C-263G-315.1C-315.2C-373G
H	AUT	hf2H11	750G-4769G-16124C-16519C-146C-263G-315.1C
H	AUT	hf2H2	750G-4769G-16519C-152C-263G-315.1C
H	AUT	hf2H3	750G-4769G-16234T-16293G-16519C-263G-315.1C-315.2C-315.3C
H	AUT	hf2H4	750G-4769G-16264T-16519C-146C-263G-309.1C-315.1C
H	AUT	hf2H8	750G-4769G-16093C-16129A-16258C-16316G-16519C-263G-309.1C-315.1C
H	AUT	hf3A3	750G-4769G-16311C-263G-315.1C
H	AUT	hf3A6	750G-4769G-16093C-263G-309.1C-315.1C
H	AUT	hf3A7	750G-4769G-16519C-263G-309.1C-315.1C
H	AUT	hf3A8	750G-4769G-16519C-235G-263G-309.1C-309.2C-315.1C
H	AUT	hf3B1	750G-4769G-16093C-16129A-16258C-16316G-16519C-263G-315.1C
H	AUT	hf3B9	750G-4769G-16519C-93G-152C-263G-309.1C-315.1C
H	AUT	hf3C1	750G-4769G-16519C-263G-309.1C-315.1C
H	AUT	hf3C10	750G-4769G-16519C-263G-309.1C-315.1C
H	AUT	hf3C4	750G-4769G-16519C-263G-315.1C
H	AUT	hf3D7	750G-4769G-16153A-16519C-263G-309.1C-309.2C-315.1C
H	AUT	hf3E11	750G-4769G-16519C-263G-309.1C-309.2C-315.1C-385G
H	AUT	hf3E3	750G-4769G-16093C-16221T-16519C-263G-309.1C-315.1C
H	AUT	hf3E5	750G-4769G-16124C-16519C-143A-263G-315.1C
H	AUT	hf3E8	750G-4769G-16519C-235G-263G-309.1C-309.2C-315.1C
H	AUT	hf3F5	750G-4769G-16266T-16311C-16399G-16519C-263G-315.1C-523DEL-524DEL
H	AUT	hf3F9	750G-4769G-7337A-16093C-16189C-16193.1C-16193.2C-16519C-152C-263G-309.1C-309.2C-315.1C
H	AUT	hf3G4	750G-4769G-16183C-16189C-16193.1C-16519C-199C-263G-309.1C-309.2C-315.1C
H	AUT	hf3G6	750G-4769G-16311C-16519C-152C-195C-263G-315.1C
H	AUT	hf3G7	750G-4769G-709A-16172Y-263G-309.1C-315.1C
H	AUT	hf3G9	750G-4769G-16519C-263G-315.1C
H	AUT	hf3H1	750G-4769G-16519C-263G-315.1C
H	AUT	hf3H11	750G-4769G-16311C-263G-315.1C-523DEL-524DEL
H	AUT	hf3H12	750G-4769G-16519C-152C-263G-309.1C-309.2C-315.1C

H	AUT	hf3H6	750G-4769G-16261T-262T-263G-309.1C-315.1C
H	AUT	hf3H9	750G-4769G-16266T-16311C-16399G-16519C-263G-315.1C-523DEL-524DEL
H	AUT	hf4A3	750G-4769G-16189C-16519C-152C-263G-309.1C-309.2C-315.1C-385G
H	AUT	hf4A5	750G-4769G-263G-309.1C-315.1C
H	AUT	hf4B11	750G-4769G-16145A-16519C-263G-315.1C
H	AUT	hf4B7	750G-4769G-16266T-16311C-16399G-16519C-263G-309.1C-315.1C-523DEL-524DEL
H	AUT	hf4C11	750G-4769G-709A-16172C-16292T-16299G-16519C-263G-315.1C
H	AUT	hf4C4	750G-4769G-7337A-16114T-16519C-200G-263G-309.1C-315.1C
H	AUT	hf4C7	750G-4769G-16519C-152C-263G-309.1C-315.1C-523DEL-524DEL
H	AUT	hf4C9	750G-4769G-16093C-16129A-16258C-16316G-16519C-263G-315.1C
H	AUT	hf4D10	750G-4769G-16129A-207A-263G-309.1C-315.1C
H	AUT	hf4D7	750G-4769G-16170G-16390A-16519C-263G-315.1C
H	AUT	hf4D8	750G-4769G-16519C-146C-195C-263G-315.1C
H	AUT	hf4D9	750G-4769G-709A-16320T-16368C-16519C-263G-309.1C-315.1C
H	AUT	hf4E11	750G-4769G-16183C-16189C-16193.1C-16519C-199C-204C-263G-309.1C-309.2C-315.1C
H	AUT	hf4E3	750G-4769G-16519C-263G-315.1C-499A
H	AUT	hf4E7	750G-4769G-16234T-16293G-16519C-263G-315.1C-315.2C
H	AUT	hf4F4	750G-4769G-16261T-16286T-262T-263G-309.1C-315.1C
H	AUT	hf4F8	750G-4769G-16519C-263G-309.1C-315.1C
H	AUT	hf4G1	750G-4769G-16261T-16519C-200G-263G-309.1C-309.2C-315.1C
H	AUT	hf4G11	750G-4769G-16355T-16519C-16524C-152C-263G-315.1C
H	AUT	hf4G2	750G-4769G-16519C-263G-309.1C-315.1C
H	AUT	hf4G3	750G-4769G-16519C-263G-309.1C-315.1C
H	AUT	hf4G4	750G-4769G-16261T-16291T-16355T-16519C-200G-263G-309.1C-315.1C
H	AUT	hf4G7	750G-4769G-16093C-16278T-16519C-263G-309.1C-315.1C
H	AUT	hf4H10	750G-4769G-16519C-263G-315.1C
H	AUT	hf4H4	750G-4769G-16124C-16189C-16193.1C-16193.2C-16240G-16519C-146C-263G-309.1C-309.2C-315.1C
H	AUT	hf4H8	750G-4769G-16189C-16192T-16519C-263G-315.1C
H	AUT	hm1A11	750G-4769G-16519C-263G-309.1C-315.1C
H	AUT	hm1A4	750G-4769G-16519C-146C-195C-263G-309.1C-315.1C
H	AUT	hm1A7	750G-4769G-16261T-16291T-16311C-16519C-200G-263G-309.1C-315.1C
H	AUT	hm1B8	750G-4769G-16519C-263G-315.1C
H	AUT	hm1C5	750G-4769G-16519C-263G-309.1C-309.2C-315.1C
H	AUT	hm1C7	750G-4769G-16519C-263G-309.1C-309.2C-315.1C
H	AUT	hm1D10	750G-4769G-16519C-263G-315.1C
H	AUT	hm1D8	750G-4769G-16261T-16291T-16311C-16519C-200G-263G-309.1C-315.1C
H	AUT	hm1E11	750G-4769G-16266T-16311C-16399G-16519C-263G-315.1C-523DEL-524DEL
H	AUT	hm1E6	750G-4769G-16189C-16192T-16519C-263G-309.1C-315.1C
H	AUT	hm1F5	750G-4769G-16519C-152C-200G-263G-315.1C-523DEL-524DEL

H	AUT	hm1F8	750G-4769G-16519C-263G-315.1C
H	AUT	hm1G10	750G-4769G-16266T-16311C-16399G-16519C-263G-315.1C-523DEL-524DEL
H	AUT	hm1G5	750G-4769G-16519C-73G-263G-299DEL-309.1C-315.1C-533G
H	AUT	hm1H10	750G-4769G-16102C-16291T-16519C-263G-315.1C
H	AUT	hm1H12	750G-4769G-16519C-263G-315.1C
H	AUT	hm1H7	750G-4769G-16129A-207A-263G-309.1C-315.1C
H	AUT	hm2A2	750G-4769G-16183DEL-263G-315.1C-523DEL-524DEL
H	AUT	hm2A7	750G-4769G-16189C-16192T-16519C-263G-315.1C
H	AUT	hm2B1	750G-4769G-16519C-263G-309.1C-309.2C-315.1C
H	AUT	hm2B10	750G-4769G-16519C-152C-263G-315.1C
H	AUT	hm2B6	750G-4769G-16189C-16192T-16519C-263G-315.1C
H	AUT	hm2B8	750G-4769G-16189C-16192T-16519C-263G-315.1C
H	AUT	hm2B9	750G-4769G-16519C-146Y-263G-309.1C-309.2C-315.1C
H	AUT	hm2C10	750G-4769G-16093C-16519C-246C-263G-309.1C-315.1C
H	AUT	hm2C7	750G-4769G-16189C-16192T-16519C-263G-315.1C
H	AUT	hm2C8	750G-4769G-16519C-214R-263G-309.1C-309.2C-315.1C
H	AUT	hm2D2	750G-4769G-16266T-16311C-16399G-16519C-263G-315.1C-523DEL-524DEL
H	AUT	hm2D6	750G-4769G-16519C-263G-309.1C-315.1C
H	AUT	hm2E3	750G-4769G-16169T-16519C-263G-315.1C
H	AUT	hm2E4	750G-4769G-16519C-263G-309.1C-315.1C
H	AUT	hm2E7	750G-4769G-16266T-16311C-16399G-16519C-263G-315.1C-523DEL-524DEL
H	AUT	hm2E8	750G-4769G-16266T-16311C-16399G-16519C-263G-315.1C-523DEL-524DEL
H	AUT	hm2F10	750G-4769G-16189C-16261T-16319A-16519C-73G-263G-315.1C
H	AUT	hm2F2	750G-4769G-16183C-16189C-16193.1C-16265G-16519C-150T-199C-263G-309.1C-309.2C-315.1C
H	AUT	hm2F8	750G-4769G-16093C-16129A-16316G-16519C-263G-315.1C
H	AUT	hm2F9	750G-4769G-16189C-16250Y-16261T-16319A-16519C-73G-263G-315.1C
H	AUT	hm2G10	750G-4769G-16519C-263G-315.1C
H	AUT	hm2G7	750G-4769G-16189C-16192T-16519C-263G-315.1C
H	AUT	hm2H10	750G-4769G-16519C-16527T-146C-195C-263G-309.1C-315.1C
H	AUT	hm2H11	750G-4769G-16183C-16189C-16193.1C-16519C-199C-263G-309.1C-309.2C-315.1C
H	AUT	hm2H12	750G-4769G-16183C-16189C-16193.1C-16519C-263G-309.1C-309.2C-315.1C
H	AUT	hm2H2	750G-4769G-16189C-16519C-152C-263G-309.1C-315.1C-385G
H	AUT	hm2H8	750G-4769G-16261T-16291T-16311C-16519C-200G-263G-309.1C-315.1C
H	AUT	hm3A5	750G-4769G-709A-15218G-16148T-16170G-16256T-16319A-189G-193G-249G-263G-309.1C-315.1C
H	AUT	hm3A6	750G-4769G-16189C-16519C-152C-263G-309.1C-309.2C-315.1C-385G
H	AUT	hm3A7	750G-4769G-16519C-152C-263G-315.1C
H	AUT	hm3B1	750G-4769G-16189C-16192T-16519C-263G-315.1C
H	AUT	hm3B7	750G-4769G-16183C-16189C-16193.1C-16519C-199C-263G-309.1C-309.2C-309.3C-315.1C
H	AUT	hm3B9	750G-4769G-4727G-4793G-16519C-263G-315.1C

H	AUT	hm3C11	750G-4769G-16258M-16519C-263G-309.1C-315.1C
H	AUT	hm3C3	750G-4769G-16519C-263G-315.1C
H	AUT	hm3C5	750G-4769G-16183C-16189C-16193.1C-16519C-199C-263G-309.1C-309.2C-315.1C
H	AUT	hm3C7	750G-4769G-16111T-16218T-16519C-93G-235G-263G-315.1C
H	AUT	hm3D3	750G-4769G-16519C-146Y-152Y-263G-315.1C
H	AUT	hm3D5	750G-4769G-16519C-235G-263G-309.1C-315.1C
H	AUT	hm3E2	750G-4769G-16311C-16519C-152C-263G-309.1C-309.2C-315.1C
H	AUT	hm3E3	750G-4769G-16192T-16519C-263G-315.1C-315.2C-373G
H	AUT	hm3E7	750G-4769G-16183C-16189C-16519C-199C-263G-309.1C-309.2C-315.1C
H	AUT	hm3E8	750G-4769G-16519C-263G-315.1C
H	AUT	hm3F11	750G-4769G-263G-315.1C
H	AUT	hm3F7	750G-4769G-16519C-235G-263G-309.1C-309.2C-315.1C
H	AUT	hm3G3	750G-4769G-16266T-16311C-16399G-16519C-263G-315.1C-523DEL-524DEL
H	AUT	hm3G4	750G-4769G-16189C-16192T-16519C-263G-315.1C
H	AUT	hm3H1	750G-4769G-16189C-16519C-153G-204C-263G-309.1C-315.1C-523DEL-524DEL
H	AUT	hm3H10	750G-4769G-16169T-16519C-263G-315.1C-451R
H	AUT	hm3H5	750G-4769G-16261T-16291T-16311C-16519C-200G-263G-309.1C-315.1C
H	AUT	hm3H8	750G-4769G-16192T-16519C-263G-315.1C-315.2C-373G
H	AUT	hm4A2	750G-4769G-16519C-93G-146C-151T-263G-309.1C-315.1C
H	AUT	hm4A6	750G-4769G-16519C-263G-315.1C
H	AUT	hm4C10	750G-4769G-709A-16519C-152C-263G-315.1C
H	AUT	hm4C11	750G-4769G-16519C-263G-309.1C-315.1C-573.1C-573.2C
H	AUT	hm4C3	750G-4769G-16189C-16192T-16519C-263G-315.1C
H	AUT	hm4C9	750G-4769G-16301Y-16519C-263G-315.1C
H	AUT	hm4D10	750G-4769G-16519C-263G-309.1C-315.1C
H	AUT	hm4D11	750G-4769G-16111A-16222T-16519C-263G-309.1C-315.1C-319C
H	AUT	hm4D2	750G-4769G-16320T-262T-263G-309.1C-315.1C
H	AUT	hm4D3	750G-4769G-16222T-16519C-93G-263G-309.1C-315.1C-319C
H	AUT	hm4D5	750G-4769G-709A-16278T-263G-309.1C-315.1C
H	AUT	hm4D7	750G-4769G-16519C-263G-315.1C
H	AUT	hm4E11	750G-4769G-16129A-16311C-152C-263G-315.1C
H	AUT	hm4E5	750G-4769G-16192T-16519C-263G-315.1C-315.2C-373G-524.1A-524.2C
H	AUT	hm4F2	750G-4769G-16519C-263G-309.1C-315.1C
H	AUT	hm4F4	750G-4769G-16066G-16093C-16129A-16311Y-16519C-263G-309.1C-309.2C-315.1C
H	AUT	hm4F7	750G-4769G-16266T-16311C-16399G-16519C-263G-315.1C-523DEL-524DEL
H	AUT	hm4F9	750G-4769G-16519C-263G-315.1C
H	AUT	hm4G9	750G-4769G-16519C-235G-263G-309.1C-315.1C
H	AUT	hm4H7	750G-4769G-16093C-16129A-16291T-16316G-16519C-263G-309.1C-315.1C
H	AUT	hm5A2	750G-4769G-16129A-16519C-263G-315.1C

H	AUT	hm5A5	750G-4769G-16519C-152C-263G-315.1C
H	AUT	hm5A7	750G-4769G-16390A-16519C-263G-315.1C
H	AUT	hm5A9	750G-4769G-16266T-16311C-16399G-16519C-263G-315.1C-523DEL-524DEL
H	AUT	hm5B1	750G-4769G-16291T-16519C-263G-315.1C-315.2C
H	AUT	hm5B11	750G-4769G-16519C-263G-309.1C-315.1C-573.1C-573.2C-573.3C-573.4C
H	AUT	hm5B3	750G-4769G-3915A-16093C-16519C-263G-315.1C
H	AUT	hm5B9	750G-4769G-16311C-152C-249G-263G-309.1C-315.1C
H	AUT	hm5C5	750G-4769G-16207G-16213A-16216G-263G-315.1C-456T
H	AUT	hm5C8	750G-4769G-16293R-16519C-146C-263G-315.1C
H	AUT	hm5D3	750G-4769G-16240G-16519C-152C-263G-315.1C
H	AUT	hm5D5	750G-4769G-16189C-16192T-16519C-189R-263G-315.1C
H	AUT	hm5D7	750G-4769G-16519C-152C-263G-315.1C
H	AUT	hm5E1	750G-4769G-16519C-263G-309.1C-315.1C-573.1C-573.2C
H	AUT	hm5E5	750G-4769G-16311C-263G-309.1C-315.1C-523DEL-524DEL
H	AUT	hm5F4	750G-4769G-16519C-263G-315.1C
H	AUT	hm5F7	750G-4769G-16185Y-16519C-263G-315.1C
H	AUT	hm5G11	750G-4769G-16519C-146C-195C-263G-315.1C
H	AUT	hm5G2	750G-4769G-16234T-16293G-16519C-263G-315.1C-315.2C
H	AUT	hm5G4	750G-4769G-16519C-263G-315.1C
H	AUT	hm5G7	750G-4769G-16519C-146C-195C-263G-315.1C
H	AUT	hm5H1	750G-4769G-16092C-16519C-263G-315.1C
H	AUT	hm5H10	750G-4769G-16134T-16519C-152C-263G-315.1C
H	AUT	hm5H11	750G-4769G-16209C-16261T-152C-195C-263G-309.1C-315.1C
H	AUT	hm5H12	750G-4769G-709A-16519C-152C-263G-309.1C-315.1C
H	AUT	hm5H2	750G-4769G-16519C-263G-315.1C
H	AUT	hm5H3	750G-4769G-16519C-263G-315.1C
H	AUT	hm5H5	750G-4769G-16193T-16293G-16353T-16519C-263G-315.1C
H	AUT	hm5H6	750G-4769G-16183C-16189C-16519C-146C-263G-315.1C
H	AUT	hm5H8	750G-4769G-16519C-263G-315.1C
H	AUT	hm6A3	750G-4769G-16519C-263G-315.1C
H	AUT	hm6A4	750G-4769G-16519C-263G-309.1C-309.2C-315.1C
H	AUT	hm6A6	750G-4769G-16189C-16519C-153G-204C-263G-309.1C-315.1C-523DEL-524DEL
H	AUT	hm6B5	750G-4769G-16519C-263G-315.1C
H	AUT	hm6B6	750G-4769G-16519C-263G-309.1C-315.1C
H	AUT	hm6B8	750G-4769G-16093C-16221T-16519C-263G-309.1C-315.1C
H	AUT	hm6C7	750G-4769G-16519C-263G-315.1C
H	AUT	hm6D4	750G-4769G-16519C-263G-309.1C-315.1C
H	AUT	hm6E1	750G-4769G-16519C-263G-309.1C-315.1C
H	AUT	hm6E7	750G-4769G-16111T-16218T-16519C-93G-235G-263G-315.1C

H	AUT	hm6F1	750G-4769G-16519C-263G-315.1C-499A
H	AUT	hm6F5	750G-4769G-16519C-263G-309.1C-315.1C
H	AUT	hm6F7	750G-4769G-16261T-16291T-16311C-16519C-200G-263G-309.1C-315.1C-573.1C
H	AUT	hm6F8	750G-4769G-16129A-16311C-152C-263G-315.1C
H	AUT	hm6G3	750G-4769G-150T-263G-309.1C-309.2C-315.1C
H	AUT	hm6G7	750G-4769G-16266T-16311C-16399G-16519C-263G-315.1C-523DEL-524DEL
H	AUT	hm6H1	750G-4769G-16298C-16352C-16519C-73G-263G-315.1C
H	AUT	hm6H5	750G-4769G-16129A-16311C-152C-263G-315.1C
H	AUT	hm6H6	750G-4769G-16325C-16519C-263G-309.1C-315.1C
H	AUT	hm6H7	750G-4769G-16519C-263G-309.1C-315.1C-524.1A-524.2C
H	AUT	LM01	750G-4769G-16266T-16311C-16399G-16519C-263G-315.1C-523DEL-524DEL
H	AUT	LM02	750G-4769G-16243C-16519C-263G-315.1C
H	AUT	m1B4	750G-4769G-16519C-16527T-146C-195C-263G-315.1C
H	AUT	m1D2	750G-4769G-16234T-16293G-16519C-263G-315.1C-315.2C
H	AUT	m1F2	750G-4769G-16519C-152C-263G-315.1C
H	AUT	m1F3	750G-4769G-16170G-16390A-16519C-263G-309.1C-315.1C
H	AUT	m2B3	750G-4769G-16261T-16291T-16311C-16519C-200G-263G-309.1C-315.1C
H	AUT	m2C2	750G-4769G-16124C-16519C-263G-309.1C-315.1C-573.1C-573.2C-573.3C
H	AUT	m2C6	750G-4769G-16519C-263G-315.1C
H	AUT	m2D3	750G-4769G-16129A-16519C-263G-309.1C-309.2C-315.1C
H	AUT	m2E4	750G-4769G-16519C-263G-309.1C-315.1C
H	AUT	m2F4	750G-4769G-16519C-16527T-146C-195C-263G-315.1C
H	AUT	m2G1	750G-4769G-16170G-16355T-16390A-16519C-263G-315.1C-478G-523DEL-524DEL
H	AUT	m3A4	750G-4769G-16183C-16189C-16519C-263G-309.1C-309.2C-315.1C
H	AUT	m3A5	750G-4769G-16183C-16189C-16519C-199C-263G-309.1C-309.2C-315.1C
H	AUT	m3F6	750G-4769G-16183C-16189C-16519C-199C-263G-309.1C-309.2C-315.1C
H	AUT	m3G1	750G-4769G-16519C-72G-263G-315.1C
H	AUT	m3G5	750G-4769G-16093C-16519C-246C-263G-309.1C-309.2C-315.1C
H	AUT	m3G6	750G-4769G-16216R-16311C-16519C-152C-263G-309.1C-309.2C-315.1C
H	AUT	m3H5	750G-4769G-262T-263G-309.1C-315.1C
H	MKD	MC1A5	750G-4769G-16189C-16519C-146C-153G-195Y-204C-263G-309.1C-309.2C-315.1C
H	MKD	MC1B6	750G-4769G-16519C-73G-263G-315.1C-573.1C-573.2C
H	MKD	MC1C12	750G-4769G-16519C-263G-315.1C
H	MKD	MC1C2	750G-4769G-16519C-263G-309.1C-315.1C
H	MKD	MC1C8	750G-4769G-16519C-186A-263G-309.1C-315.1C
H	MKD	MC1D10	750G-4769G-16293C-262T-263G-309.1C-309.2C-315.1C
H	MKD	MC1D12	750G-4769G-16147T-16519C-195C-263G-315.1C-523DEL-524DEL
H	MKD	MC1E11	750G-4769G-709A-15218G-16148T-16256T-16319A-152C-189G-193G-249G-309.1C-315.1C
H	MKD	MC1F12	750G-4769G-16189C-16519C-146C-153G-263G-309.1C-315.1C

H	MKD	MC1G4	750G-4769G-16189C-16519C-153G-263G-309.1C-309.2C-315.1C
H	MKD	MC1G9	750G-4769G-16519C-152C-215G-263G-309.1C-309.2C-315.1C
H	MKD	MC1H12	750G-4769G-16189C-16519C-204C-263G-309.1C-309.2C-315.1C
H	MKD	MC1H4	750G-4769G-16189C-16193.1C-16193.2C-16519C-146C-153G-195Y-204C-263G-309.1C-309.2C-315.1C
H	MKD	MC1H5	750G-4769G-16311C-263G-315.1C
H	MKD	MC2A10	750G-4769G-16519C-146C-263G-315.1C
H	MKD	MC2D11	750G-4769G-16093Y-16311C-16519C-152C-263G-315.1C
H	MKD	MC2D4	750G-4769G-16189C-16519C-146C-153R-263G-309.1C-309.2C-315.1C
H	MKD	MC2E12	750G-4769G-16311C-263G-315.1C
H	MKD	MC2F3	750G-4769G-16129A-16291T-16519C-16527T-152C-263G-309.1C-309.2C-315.1C-437T
H	MKD	MC2G2	750G-4769G-16519C-152C-263G-295T-309.1C-309.2C-315.1C
H	MKD	MC2G9	750G-4769G-16519C-146C-263G-315.1C
H	MKD	MC2H1	750G-4769G-16189C-16519C-153G-263G-309.1C-315.1C
H	MKD	MC2H12	750G-4769G-4745G-8473C-263G-315.1C
H	MKD	MC2H5	750G-4769G-16093C-16311C-16519C-152C-263G-315.1C
H	ROU	SZ1A2	750G-4769G-16519C-93G-249DEL-263G-309.1C-315.1C
H	ROU	SZ1A9	750G-4769G-16223T-16519C-263G-315.1C
H	ROU	SZ1B12	750G-4769G-16093C-16169T-16311C-16519C-152C-257G-263G-315.1C
H	ROU	SZ1B3	750G-4769G-16278T-143A-204Y-263G-308.1T-315.1C
H	ROU	SZ1B4	750G-4769G-16188.1C-16311C-152C-263G-315.1C
H	ROU	SZ1D10	750G-4769G-16189Y-16519C-263G-315.1C
H	ROU	SZ1D6	750G-4769G-16293G-16519C-263G-309.1C-315.1C
H	ROU	SZ1F2	750G-4769G-16189C-16193.1C-16519C-152C-263G-315.1C-523DEL-524DEL
H	ROU	SZ1G8	750G-4769G-16218T-16278T-93G-263G-315.1C
H	ROU	SZ2A9	750G-4769G-16129A-16316G-16519C-63C-64T-66A-150T-263G-309.1C-309.2C-315.1C
H	ROU	SZ2E11	750G-4769G-16519C-263G-309.1C-315.1C
H	ROU	SZ2F7	750G-4769G-16037G-263G-309.1C-315.1C
H	DEU	UL1B8	750G-4769G-263G-309.1C-315.1C
H	DEU	UL1E5	750G-4769G-16519C-146C-195C-263G-315.1C
H	DEU	UL1E7	750G-4769G-16519C-263G-315.1C-523DEL-524DEL
H	DEU	UL1F10	750G-4769G-709A-16319A-16325C-16519C-263G-309.1C-315.1C
H	DEU	UL1F12	750G-4769G-16086C-16261G-16519C-263G-315.1C
H	DEU	UL1F7	750G-4769G-16189C-16291T-16519C-152C-263G-309.1C-315.1C
H	DEU	UL1F8	750G-4769G-16129A-16316G-16519C-263G-315.1C
H	DEU	UL1H7	750G-4769G-16129A-16316G-16519C-263G-315.1C
H1	HUN	As1B5	750G-4769G-3010A-16234Y-16519C-263G-309.1C-315.1C
H1	HUN	As1C12	750G-4769G-3010A-16519C-263G-309.1C-309.2C-315.1C
H1	HUN	As1E8	750G-4769G-3010A-16172C-16192T-16327Y-16456A-16519C-207A-263G-315.1C
H1	HUN	As1F10	750G-4769G-3010A-16519C-263G-267C-309.1C-315.1C-485C

H1	HUN	As2B4	750G-4769G-3010A-16519C-263G-309.1C-315.1C
H1	HUN	As2C3	750G-4769G-3010A-16172C-16192T-16456A-16519C-152C-207A-263G-315.1C
H1	HUN	As2D12	750G-4769G-3010A-16519C-263G-309.1C-315.1C
H1	HUN	As2D4	750G-4769G-3010A-16519C-228A-263G-309.1C-309.2C-315.1C
H1	HUN	As2D8	750G-4769G-3010A-16519C-263G-309.1C-315.1C
H1	HUN	As2E1	750G-4769G-3010A-16519C-263G-309.1C-315.1C
H1	HUN	As2E12	750G-4769G-3010A-16519C-263G-309.1C-315.1C
H1	HUN	As2H11	750G-4769G-3010A-16519C-263G-309.1C-315.1C
H1	ROU	CS1A11	750G-4769G-3010A-16319A-16519C-263G-315.1C
H1	ROU	CS1F4	750G-4769G-3010A-16319A-16519C-263G-309.1C-315.1C
H1	ROU	CS2B10	750G-4769G-3010A-16319A-16519C-263G-315.1C
H1	ROU	CS2C2	750G-4769G-3010A-16319A-16519C-263G-315.1C
H1	ARE	DB1D6	750G-4769G-3010A-16519C-263G-315.1C
H1	AUT	f1A6	750G-4769G-3010A-16037G-16188A-16519C-263G-309.1C-309.2C-315.1C-524.1A-524.2C
H1	AUT	f1G3	750G-4769G-3010A-16325C-16519C-263G-309.1C-315.1C
H1	AUT	f1H2	750G-4769G-3010A-7337A-16519C-153G-263G-309.1C-315.1C
H1	AUT	f2A4	750G-4769G-3010A-8602C-16189C-16519C-263G-315.1C
H1	AUT	f2B5	750G-4769G-3010A-16189C-16519C-263G-315.1C
H1	AUT	f2D1	750G-4769G-3010A-16519C-146C-263G-309.1C-315.1C
H1	AUT	f2E5	750G-4769G-3010A-16519C-263G-309.1C-315.1C
H1	AUT	f3A5	750G-4769G-3010A-16519C-146C-263G-309.1C-315.1C
H1	AUT	f3B4	750G-4769G-3010A-709A-16519C-259G-263G-315.1C
H1	AUT	hf1A9	750G-4769G-3010A-16295T-16519C-195C-263G-309.1C-315.1C-516T
H1	AUT	hf1C9	750G-4769G-3010A-16519C-247A-263G-315.1C
H1	AUT	hf1D3	750G-4769G-3010A-16188G-16357C-16519C-93G-235G-263G-309.1C-315.1C
H1	AUT	hf1D4	750G-4769G-3010A-16519C-73G-150T-198T-263G-315.1C
H1	AUT	hf1D6	750G-4769G-3010A-16519C-263G-315.1C
H1	AUT	hf1D9	750G-4769G-3010A-16519C-247A-263G-315.1C
H1	AUT	hf1E4	750G-4769G-3010A-16519C-263G-315.1C
H1	AUT	hf1F10	750G-4769G-3010A-16519C-263G-315.1C
H1	AUT	hf1G9	750G-4769G-3010A-16184T-16519C-146C-263G-315.1C
H1	AUT	hf2A2	750G-4769G-3010A-16076T-16172C-16519C-263G-309.1C-315.1C-524.1A-524.2C-524.3A-524.4C
H1	AUT	hf2B8	750G-4769G-3010A-6253C-16093C-16519C-263G-315.1C
H1	AUT	hf2D11	750G-4769G-3010A-16114G-16519C-152C-263G-309.1C-315.1C-453C
H1	AUT	hf2D4	750G-4769G-3010A-16519C-263G-315.1C
H1	AUT	hf2E4	750G-4769G-3010A-16299G-16519C-146C-263G-309.1C-309.2C-315.1C
H1	AUT	hf2F8	750G-4769G-3010A-7337A-16519C-195C-263G-315.1C
H1	AUT	hf2G12	750G-4769G-3010A-16189C-16519C-263G-315.1C
H1	AUT	hf3B2	750G-4769G-3010A-16519C-263G-315.1C

H1	AUT	hf3B6	750G-4769G-3010A-16519C-263G-315.1C
H1	AUT	hf3D1	750G-4769G-3010A-16519C-263G-309.1C-315.1C
H1	AUT	hf3D10	750G-4769G-3010A-16519C-263G-315.1C
H1	AUT	hf3E1	750G-4769G-3010A-16294T-16519C-263G-309.1C-315.1C
H1	AUT	hf3E2	750G-4769G-3010A-16519C-263G-309.1C-315.1C
H1	AUT	hf3F1	750G-4769G-3010A-16129A-16298C-16519C-214G-263G-309.1C-315.1C
H1	AUT	hf3H2	750G-4769G-3010A-16114G-16519C-152C-263G-309.1C-315.1C-453C
H1	AUT	hf4A4	750G-4769G-3010A-16189C-16233T-16519C-152C-263G-315.1C
H1	AUT	hf4C6	750G-4769G-3010A-16193T-16519C-263G-309.1C-315.1C
H1	AUT	hf4D1	750G-4769G-3010A-16519C-263G-315.1C
H1	AUT	hf4E9	750G-4769G-3010A-16519C-263G-309.1C-315.1C
H1	AUT	hf4F1	750G-4769G-3010A-16239T-16519C-263G-315.1C
H1	AUT	hf4F11	750G-4769G-3010A-16213A-16291T-16519C-263G-309.1C-309.2C-315.1C-316A
H1	AUT	hf4F12	750G-4769G-3010A-16519C-263G-309.1C-315.1C
H1	AUT	hf4F5	750G-4769G-3010A-16519C-263G-309.1C-315.1C
H1	AUT	hf4F7	750G-4769G-3010A-16519C-263G-309.1C-315.1C
H1	AUT	hf4G6	750G-4769G-3010A-16183C-16189C-16193.1C-16290T-16519C-263G-315.1C
H1	AUT	hf4H1	750G-4769G-3010A-16209C-16519C-263G-315.1C
H1	AUT	hm1A6	750G-4769G-3010A-16519C-263G-315.1C
H1	AUT	hm1A9	750G-4769G-3010A-16189C-16311C-16519C-263G-309.1C-315.1C-327T
H1	AUT	hm1B4	750G-4769G-3010A-16129A-16256T-16519C-263G-309.1C-315.1C
H1	AUT	hm1C6	750G-4769G-3010A-16076T-16172C-16519C-263G-309.1C-315.1C-524.1A-524.2C-524.3A-524.4C
H1	AUT	hm1D2	750G-4769G-3010A-16519C-146C-263G-309.1C-315.1C
H1	AUT	hm1E1	750G-4769G-3010A-16519C-263G-309.1C-309.2C-315.1C
H1	AUT	hm1E4	750G-4769G-3010A-16519C-263G-315.1C
H1	AUT	hm1F6	750G-4769G-3010A-16189C-16193.1C-16193.2C-16311C-16519C-263G-309.1C-315.1C-327T
H1	AUT	hm1G11	750G-4769G-3010A-16519C-263G-315.1C
H1	AUT	hm1G2	750G-4769G-3010A-16189C-16519C-152C-263G-315.1C
H1	AUT	hm1G3	750G-4769G-3010A-16182C-16183C-16189C-16519C-263G-315.1C
H1	AUT	hm1H11	750G-4769G-3010A-16519C-146C-263G-315.1C
H1	AUT	hm1H5	750G-4769G-3010A-16519C-146C-263G-315.1C
H1	AUT	hm2A1	750G-4769G-3010A-16519C-263G-315.1C
H1	AUT	hm2A3	750G-4769G-3010A-16170T-16519C-263G-309.1C-315.1C
H1	AUT	hm2A5	750G-4769G-3010A-7337A-16519C-195C-263G-315.1C
H1	AUT	hm2A8	750G-4769G-3010A-16519C-146C-263G-309.1C-315.1C
H1	AUT	hm2B11	750G-4769G-3010A-16519C-146C-263G-309.1C-309.2C-315.1C
H1	AUT	hm2C6	750G-4769G-3010A-16519C-146C-263G-309.1C-315.1C
H1	AUT	hm2C9	750G-4769G-3010A-16519C-146C-263G-309.1C-315.1C
H1	AUT	hm2D11	750G-4769G-3010A-16519C-263G-309.1C-315.1C

H1	AUT	hm2F3	750G-4769G-3010A-16360T-16519C-152C-263G-309.1C-315.1C
H1	AUT	hm2F4	750G-4769G-3010A-16519C-263G-309.1C-309.2C-315.1C
H1	AUT	hm2H1	750G-4769G-3010A-16519C-146C-263G-309.1C-309.2C-315.1C
H1	AUT	hm3B10	750G-4769G-3010A-16519C-263G-309.1C-315.1C
H1	AUT	hm3B11	750G-4769G-3010A-16299G-16519C-146C-263G-309.1C-309.2C-315.1C
H1	AUT	hm3B3	750G-4769G-3010A-16519C-146C-263G-309.1C-315.1C
H1	AUT	hm3B8	750G-4769G-3010A-6253C-16093C-16519C-263G-315.1C
H1	AUT	hm3C1	750G-4769G-3010A-16519C-146C-263G-309.1C-315.1C
H1	AUT	hm3C2	750G-4769G-3010A-16519C-263G-315.1C
H1	AUT	hm3C4	750G-4769G-3010A-16519C-263G-315.1C
H1	AUT	hm3C9	750G-4769G-3010A-16519C-263G-309.1C-315.1C-453C
H1	AUT	hm3D1	750G-4769G-3010A-16519C-146C-263G-309.1C-315.1C
H1	AUT	hm3D11	750G-4769G-3010A-16183C-16189C-16209C-16519C-199Y-263G-315.1C
H1	AUT	hm3D2	750G-4769G-3010A-16519C-146C-263G-309.1C-309.2C-315.1C
H1	AUT	hm3D4	750G-4769G-3010A-16189C-16519C-152C-263G-315.1C
H1	AUT	hm3E5	750G-4769G-3010A-16355Y-16519C-263G-315.1C
H1	AUT	hm3E6	750G-4769G-3010A-16519C-263G-315.1C
H1	AUT	hm3F8	750G-4769G-3010A-16519C-263G-315.1C
H1	AUT	hm3G11	750G-4769G-3010A-8602C-16189C-16519C-263G-315.1C
H1	AUT	hm4A1	750G-4769G-3010A-16519C-152C-263G-309.1C-315.1C
H1	AUT	hm4B11	750G-4769G-3010A-16519C-146C-263G-315.1C
H1	AUT	hm4B2	750G-4769G-3010A-16235G-16317G-16519C-263G-309.1C-315.1C-523DEL-524DEL
H1	AUT	hm4B5	750G-4769G-3010A-16230G-16249C-16519C-263G-309.1C-315.1C
H1	AUT	hm4B6	750G-4769G-3010A-16175G-16519C-263G-309.1C-309.2C-315.1C
H1	AUT	hm4E1	750G-4769G-3010A-16519C-263G-315.1C
H1	AUT	hm4E10	750G-4769G-3010A-16239T-16519C-263G-315.1C
H1	AUT	hm4E3	750G-4769G-3010A-16188T-16311C-16519C-239C-263G-309.1C-309.2C-315.1C
H1	AUT	hm4E4	750G-4769G-3010A-16519C-263G-315.1C
H1	AUT	hm4F10	750G-4769G-3010A-16519C-263G-309.1C-315.1C
H1	AUT	hm4G12	750G-4769G-3010A-16519C-146C-263G-309.1C-315.1C
H1	AUT	hm4G7	750G-4769G-3010A-16189C-16193.1C-16519C-152C-263G-309.1C-309.2C-315.1C
H1	AUT	hm4H4	750G-4769G-3010A-16076T-16172C-16519C-263G-309.1C-315.1C-524.1A-524.2C-524.3A-524.4C
H1	AUT	hm4H6	750G-4769G-3010A-16519C-263G-315.1C
H1	AUT	hm5A3	750G-4769G-3010A-709A-16519C-259G-263G-315.1C
H1	AUT	hm5A4	750G-4769G-3010A-16189C-16519C-263G-309.1C-315.1C
H1	AUT	hm5A6	750G-4769G-3010A-16519C-146C-263G-309.1C-315.1C
H1	AUT	hm5B2	750G-4769G-3010A-16519C-263G-315.1C
H1	AUT	hm5C10	750G-4769G-3010A-16209G-16519C-263G-315.1C
H1	AUT	hm5C11	750G-4769G-3010A-6253C-16093C-16519C-263G-315.1C

H1	AUT	hm5C3	750G-4769G-3010A-16519C-263G-309.1C-315.1C-453C
H1	AUT	hm5C6	750G-4769G-3010A-16189C-16519C-263G-315.1C
H1	AUT	hm5D11	750G-4769G-3010A-6253C-16093C-16519C-263G-315.1C
H1	AUT	hm5E10	750G-4769G-3010A-7337A-16519C-195C-263G-315.1C
H1	AUT	hm5E3	750G-4769G-3010A-16037G-16304C-16519C-263G-315.1C
H1	AUT	hm5F9	750G-4769G-3010A-16519C-263G-315.1C
H1	AUT	hm6A2	750G-4769G-3010A-16325C-16519C-263G-309.1C-315.1C
H1	AUT	hm6A7	750G-4769G-3010A-16519C-263G-315.1C
H1	AUT	hm6B4	750G-4769G-3010A-6253C-16093C-16519C-263G-315.1C
H1	AUT	hm6C4	750G-4769G-3010A-16189C-16264T-16519C-146C-263G-309.1C-315.1C
H1	AUT	hm6D3	750G-4769G-3010A-16192T-16270T-16291T-16519C-263G-315.1C
H1	AUT	hm6E2	750G-4769G-3010A-16172Y-16519C-263G-309.1C-309.2C-315.1C
H1	AUT	m1B3	750G-4769G-3010A-16239T-16355Y-16519C-263G-309.1C-315.1C
H1	AUT	m1F1	750G-4769G-3010A-16519C-146C-263G-309.1C-309.2C-315.1C
H1	AUT	m2B6	750G-4769G-3010A-16519C-263G-309.1C-315.1C
H1	AUT	m2F1	750G-4769G-3010A-16239T-16311C-16519C-263G-315.1C
H1	AUT	m2F3	750G-4769G-3010A-16249C-16519C-263G-315.1C
H1	AUT	m2H6	750G-4769G-3010A-6296T-16519C-263G-309.1C-315.1C
H1	AUT	m3D3	750G-4769G-3010A-16519C-263G-315.1C
H1	AUT	m3E5	750G-4769G-3010A-16207G-16519C-152C-263G-315.1C
H1	AUT	m3H1	750G-4769G-3010A-16256T-16311C-16519C-263G-315.1C
H1	AUT	m3H4	750G-4769G-3010A-16249C-16519C-263G-309.1C-315.1C
H1	MKD	MC1B11	750G-4769G-3010A-16519C-152C-263G-315.1C
H1	MKD	MC1B2	750G-4769G-3010A-16519C-146C-263G-309.1C-309.2C-315.1C
H1	MKD	MC1B3	750G-4769G-3010A-16519C-263G-309.1C-309.2C-315.1C
H1	MKD	MC1C5	750G-4769G-3010A-16311C-16519C-263G-309.1C-309.2C-315.1C
H1	MKD	MC1D3	750G-4769G-3010A-16129A-16270T-16519C-263G-315.1C-481T
H1	MKD	MC1E5	750G-4769G-3010A-16176T-16182C-16183C-16189C-16519C-263G-309.1C-309.2C-309.3C-315.1C
H1	MKD	MC1H11	750G-4769G-3010A-16259T-16519C-263G-309.1C-309.2C-315.1C-316A
H1	MKD	MC2B4	750G-4769G-3010A-16519C-143A-263G-315.1C-453C
H1	MKD	MC2G7	750G-4769G-3010A-16182C-16183C-16189C-16519C-263G-309.1C-309.2C-315.1C
H1	MKD	MC3B1	750G-4769G-3010A-16217Y-16519C-263G-309.1C-309.2C-315.1C-453C
H1	ROU	SZ1A10	750G-4769G-3010A-16319A-16519C-263G-315.1C
H1	ROU	SZ1A3	750G-4769G-3010A-16519C-263G-309.1C-315.1C
H1	ROU	SZ1B8	750G-4769G-3010A-16086Y-16150T-16183C-16189C-16519C-73G-263G-315.1C
H1	ROU	SZ1C6	750G-4769G-3010A-16189C-16193.1C-16243C-16519C-73G-152C-263G-315.1C
H1	ROU	SZ1D4	750G-4769G-3010A-16066G-16209C-16519C-263G-309.1C-309.2C-315.1C
H1	ROU	SZ1E10	750G-4769G-3010A-16239T-16519C-263G-309.1C-315.1C
H1	ROU	SZ1F1	750G-4769G-3010A-16239T-16519C-263G-309.1C-315.1C

H1	ROU	SZ1F9	750G-4769G-3010A-16126C-16519C-263G-309.1C-315.1C
H1	ROU	SZ1G10	750G-4769G-3010A-16319A-16519C-263G-315.1C
H1	ROU	SZ2A3	750G-4769G-3010A-16519C-263G-315.1C
H1	ROU	SZ2G3	750G-4769G-3010A-16519C-263G-315.1C
H1	ROU	SZ2G7	750G-4769G-3010A-16519C-263G-315.1C
H1	DEU	UL1A6	750G-4769G-3010A-8602C-16183C-16189C-16519C-187T-263G-315.1C
H1	DEU	UL1B11	750G-4769G-3010A-16519C-263G-309.1C-315.1C
H1	DEU	UL1F9	750G-4769G-3010A-16189C-16519C-73G-263G-279C-315.1C-523DEL-524DEL
H10	HUN	As1E3	750G-4769G-14470A-16114T-16344T-16519C-263G-309.1C-309.2C-315.1C-513A
H10	HUN	As1G4	750G-4769G-14470A-16344T-16519C-263G-309.1C-309.2C-315.1C-513A
H10	HUN	As2F11	750G-4769G-14470A-16114T-16344T-16519C-263G-309.1C-309.2C-315.1C-513A
H10	AUT	f2F5	750G-4769G-14470A-16519C-152C-263G-309.1C-315.1C-334C-524.1A-524.2C
H10	AUT	f3E4	750G-4769G-14470A-16519C-263G-309.1C-315.1C
H10	AUT	hf1A8	750G-4769G-14470A-16114T-16291T-16519C-263G-315.1C
H10	AUT	hf1F11	750G-4769G-14470A-16519C-263G-309.1C-315.1C
H10	AUT	hf1G3	750G-4769G-14470A-16519C-263G-309.1C-309.2C-315.1C
H10	AUT	hf2A6	750G-4769G-14470A-16093C-16221T-16519C-263G-309.1C-315.1C
H10	AUT	hf2D2	750G-4769G-14470A-16519C-263G-309.1C-309.2C-315.1C
H10	AUT	hf2G4	750G-4769G-14470A-16519C-263G-309.1C-315.1C
H10	AUT	hf3B10	750G-4769G-14470A-16519C-263G-309.1C-309.2C-315.1C
H10	AUT	hf3C3	750G-4769G-14470A-16221T-16519C-263G-315.1C
H10	AUT	hf3C7	750G-4769G-14470A-16519C-263G-309.1C-315.1C
H10	AUT	hf4B9	750G-4769G-14470A-16519C-263G-309.1C-315.1C
H10	AUT	hf4C2	750G-4769G-14470A-16519C-263G-309.1C-315.1C
H10	AUT	hf4E1	750G-4769G-14470A-16519C-263G-309.1C-315.1C
H10	AUT	hf4E5	750G-4769G-14470A-16093C-16221T-16519C-263G-309.1C-315.1C
H10	AUT	hm1C9	750G-4769G-14470A-16114T-16291T-16519C-182Y-263G-315.1C
H10	AUT	hm1G9	750G-4769G-14470A-16093C-16519C-263G-309.1C-315.1C
H10	AUT	hm1H9	750G-4769G-14470A-16093C-16221T-16519C-263G-309.1C-315.1C
H10	AUT	hm2D3	750G-4769G-14470A-16519C-263G-309.1C-315.1C
H10	AUT	hm2D9	750G-4769G-14470A-16114T-16187Y-16291T-16519C-263G-315.1C
H10	AUT	hm2G11	750G-4769G-14470A-16519C-263G-315.1C
H10	AUT	hm2G6	750G-4769G-14470A-16519C-263G-309.1C-315.1C
H10	AUT	hm3A8	750G-4769G-14470A-16519C-263G-309.1C-315.1C
H10	AUT	hm3D10	750G-4769G-14470A-16519C-263G-309.1C-309.2C-315.1C
H10	AUT	hm4B4	750G-4769G-14470A-16221T-16519C-263G-309.1C-309.2C-315.1C
H10	AUT	hm4D8	750G-4769G-14470A-16519C-263G-309.1C-309.2C-315.1C
H10	AUT	hm4F1	750G-4769G-14470A-16519C-263G-309.1C-315.1C
H10	AUT	hm4F11	750G-4769G-14470A-16519C-263G-309.1C-315.1C

H10	AUT	hm4G5	750G-4769G-14470A-16519C-263G-309.1C-315.1C
H10	AUT	hm4H11	750G-4769G-14470A-16221T-16519C-263G-309.1C-315.1C
H10	AUT	hm5C4	750G-4769G-14470A-16519C-263G-309.1C-315.1C
H10	AUT	hm6B1	750G-4769G-14470A-16093C-16221T-16519C-263G-309.1C-315.1C
H10	AUT	hm6G1	750G-4769G-14470A-16093C-16221T-16519C-263G-309.1C-315.1C
H10	AUT	m1E2	750G-4769G-14470A-16519C-263G-309.1C-315.1C
H10	AUT	m1E5	750G-4769G-14470A-16519C-263G-309.1C-315.1C
H10	AUT	m3B6	750G-4769G-14470A-16114T-16519C-263G-309.1C-315.1C
H10	AUT	m3G3	750G-4769G-14470A-16519C-152C-263G-309.1C-315.1C
H10	MKD	MC1B7	750G-4769G-14470A-16114Y-16344T-16519C-263G-309.1C-315.1C-513A
H10	MKD	MC1E3	750G-4769G-14470A-16114T-16344T-16519C-263G-309.1C-309.2C-315.1C-513A
H10	DEU	UL1A8	750G-4769G-14470A-16093C-16221T-16519C-263G-309.1C-315.1C
H10	DEU	UL1B3	750G-4769G-14470A-16093C-16221T-16519C-263G-315.1C
H11	ROU	CS2E1	750G-4769G-13759A-16058T-16192T-16242T-16261T-152C-263G-309.1C-315.1C
H11	ROU	CS2E6	750G-4769G-13759A-16192T-16242T-16261T-152C-263G-309.1C-315.1C
H11	ARE	DB3C9	750G-4769G-13759A-16219G-16261T-16519C-146C-263G-309.1C-315.1C-523DEL-524DEL
H11	ARE	DB3F10	750G-4769G-13759A-16219G-16261T-16519C-146C-263G-309.1C-315.1C-523DEL-524DEL
H11	AUT	hf3D11	750G-4769G-13759A-16519C-152C-263G-315.1C-523DEL-524DEL
H11	AUT	hm1F11	750G-4769G-13759A-16278T-16311C-195C-263G-309.1C-315.1C
H11	AUT	hm4E8	750G-4769G-13759A-16311DEL-93G-152C-195C-263G-309.1C-315.1C
H11	AUT	m2E6	750G-4769G-13759A-16519C-152C-263G-315.1C-523DEL-524DEL
H11	MKD	MC1F1	750G-4769G-13759A-16362C-16519C-263G-315.1C
H11a	ROU	CS1C10	750G-4769G-13759A-9150G-16092C-16293G-16311C-146C-195C-263G-315.1C
H11a	ROU	CS1G1	750G-4769G-13759A-16278T-16293G-16311C-42.1G-195C-263G-309.1C-315.1C
H11a	ROU	CS2B2	750G-4769G-13759A-9150G-16092C-16293G-16311C-146C-195C-263G-315.1C
H11a	ROU	CS2C5	750G-4769G-13759A-9150G-16092C-16293G-16311C-146C-195C-263G-315.1C
H11a	ROU	CS2G9	750G-4769G-13759A-16278T-16293G-16311C-195C-263G-315.1C
H11a	AUT	f1E1	750G-4769G-13759A-16293G-16311C-143A-195C-263G-309.1C-315.1C
H11a	AUT	f2F3	750G-4769G-13759A-16293G-16294T-16311C-143A-195C-263G-309.1C-315.1C
H11a	AUT	hf1B4	750G-4769G-13759A-16293G-16311C-195C-263G-309.1C-315.1C
H11a	AUT	hf1E3	750G-4769G-13759A-16129A-16192T-16293G-16311C-195C-263G-309.1C-309.2C-315.1C
H11a	AUT	hf1F2	750G-4769G-13759A-16093C-16293G-16311C-152C-195C-263G-309.1C-315.1C
H11a	AUT	hf2D8	750G-4769G-13759A-16293G-16311C-195C-263G-309.1C-309.2C-315.1C
H11a	AUT	hf2H9	750G-4769G-13759A-16293G-16311C-152C-195C-263G-309.1C-315.1C
H11a	AUT	hf3C8	750G-4769G-13759A-16092C-16140C-16265G-16293G-16311C-195C-263G-309.1C-309.2C-315.1C
H11a	AUT	hf4B5	750G-4769G-13759A-16293G-16311C-146C-195C-263G-315.1C
H11a	AUT	hm2C5	750G-4769G-13759A-16293G-16311C-195C-263G-309.1C-315.1C
H11a	AUT	hm4H10	750G-4769G-13759A-16293G-16311C-146Y-195C-263G-309.1C-315.1C
H11a	AUT	hm5A11	750G-4769G-13759A-2581G-16172C-16293G-16311C-195C-263G-309.1C-315.1C

H11a	AUT	hm5D1	750G-4769G-13759A-16293G-16311C-195C-263G-309.1C-309.2C-315.1C
H11a	MKD	MC1C9	750G-4769G-13759A-16092C-16293G-16311C-152C-195C-263G-315.1C
H11a	MKD	MC1D11	750G-4769G-13759A-16092C-16293G-16311C-152C-195C-263G-315.1C
H11a	MKD	MC2A11	750G-4769G-13759A-16293G-16311C-195C-263G-309.1C-315.1C
H11a	MKD	MC2G1	750G-4769G-13759A-9150G-16092C-16293G-16311C-146C-195C-204Y-263G-315.1C
H11a	MKD	MC2G11	750G-4769G-13759A-16092C-16293G-16311C-152C-195C-263G-315.1C
H11a	MKD	MC2G6	750G-4769G-13759A-16278T-16293G-16311C-195C-263G-315.1C
H11a	DEU	UL1A11	750G-4769G-13759A-16293G-16311C-195C-263G-315.1C
H11a	DEU	UL1C3	750G-4769G-13759A-16193T-16293G-16311C-195C-263G-309.1C-315.1C
H12	MKD	MC1D9	750G-4769G-14552G-16287T-16519C-195C-263G-315.1C
H12	MKD	MC1F2	750G-4769G-14552G-16287T-16519C-195C-263G-315.1C
H12	MKD	MC1G11	750G-4769G-14552G-16287T-16519C-195C-263G-315.1C
H12	MKD	MC2E1	750G-4769G-14552G-16287T-16519C-195C-263G-315.1C
H12	MKD	MC2E11	750G-4769G-14552G-16287T-16519C-195C-263G-315.1C
H12	MKD	MC2G4	750G-4769G-14552G-16287T-16519C-195C-263G-315.1C
H13a1	AUT	f3D5	750G-4769G-4745G-263G-309.1C-309.2C-315.1C
H13a1	AUT	hf4B4	750G-4769G-4745G-16519C-263G-309.1C-309.2C-315.1C
H13a1	AUT	hf4H6	750G-4769G-4745G-263G-309.1C-309.2C-315.1C
H13a1	AUT	hm1G12	750G-4769G-4745G-263G-315.1C
H13a1	AUT	hm1H1	750G-4769G-4745G-263G-315.1C
H13a1	AUT	hm3A4	750G-4769G-4745G-263G-309.1C-309.2C-315.1C
H13a1	AUT	hm3F9	750G-4769G-4745G-16260T-263G-309.1C-309.2C-315.1C
H13a1	AUT	hm4E6	750G-4769G-4745G-263G-309.1C-309.2C-315.1C
H13a1	AUT	hm5E6	750G-4769G-4745G-263G-309.1C-315.1C
H13a1	AUT	hm6H3	750G-4769G-4745G-263G-309.1C-315.1C
H13a1	AUT	m1E3	750G-4769G-4745G-16519C-263G-309.1C-315.1C
H13a1	MKD	MC1B10	750G-4769G-4745G-16244A-16399G-207A-263G-315.1C
H13a1	MKD	MC1H2	750G-4769G-4745G-263G-309.1C-309.2C-315.1C
H13a1	MKD	MC2B12	750G-4769G-4745G-16244A-16399G-207A-263G-315.1C
H13a1	DEU	UL1D6	750G-4769G-4745G-16207G-228A-263G-309.1C-315.1C
H13a1a	ROU	CS2A6	750G-4769G-4745G-7337A-16266Y-152C-263G-309.1C-315.1C
H13a1a	AUT	f2H4	750G-4769G-4745G-7337A-16172Y-16327T-263G-309.1C-315.1C-438T
H13a1a	AUT	hf1D10	750G-4769G-4745G-7337A-16327T-263G-309.1C-315.1C
H13a1a	AUT	hf2E3	750G-4769G-4745G-7337A-16327T-263G-309.1C-315.1C
H13a1a	AUT	hf2F6	750G-4769G-4745G-7337A-263G-315.1C-567T
H13a1a	AUT	hf3A1	750G-4769G-4745G-7337A-263G-315.1C-567T
H13a1a	AUT	hf3E10	750G-4769G-4745G-7337A-16327T-263G-309.1C-309.2C-315.1C
H13a1a	AUT	hf3H8	750G-4769G-4745G-7337A-16327T-263G-309.1C-315.1C
H13a1a	AUT	hf4E4	750G-4769G-4745G-7337A-16278T-152C-263G-309.1C-315.1C

H13a1a	AUT	hm1B5	750G-4769G-4745G-7337A-263G-315.1C-567T
H13a1a	AUT	hm1E10	750G-4769G-4745G-7337A-16327T-263G-309.1C-315.1C-438T
H13a1a	AUT	hm1F10	750G-4769G-4745G-7337A-152C-263G-309.1C-309.2C-315.1C
H13a1a	AUT	hm1H3	750G-4769G-4745G-7337A-152C-263G-309.1C-315.1C
H13a1a	AUT	hm2B3	750G-4769G-4745G-7337A-263G-315.1C-567T
H13a1a	AUT	hm4A10	750G-4769G-4745G-7337A-16327T-263G-309.1C-315.1C
H13a1a	AUT	hm4A3	750G-4769G-4745G-7337A-16114T-16327T-263G-309.1C-309.2C-315.1C
H13a1a	AUT	hm5F2	750G-4769G-4745G-7337A-16183C-16189C-263G-309.1C-309.2C-315.1C
H13a1a	AUT	m1F5	750G-4769G-4745G-7337A-16327T-263G-309.1C-315.1C-438T
H13a1a	AUT	m2C4	750G-4769G-4745G-7337A-16327T-263G-309.1C-315.1C-438T
H13a1a	MKD	MC1B12	750G-4769G-4745G-7337A-16261T-16311C-16519C-263G-315.1C-523DEL-524DEL
H13a1a	MKD	MC3C2	750G-4769G-4745G-7337A-263G-309.1C-315.1C
H13a1a	ROU	SZ1C9	750G-4769G-4745G-7337A-152C-263G-309.1C-315.1C
H14	HUN	As1H11	750G-4769G-7645C-16261T-16311C-16519C-152C-195C-263G-309.1C-315.1C
H14	ARE	DB1A3	750G-4769G-7645C-16319A-263G-309.1C-309.2C-315.1C
H14	ARE	DB1D2	750G-4769G-7645C-16093C-16519C-263G-309.1C-309.2C-315.1C-524.1A-524.2C
H14	ARE	DB1D3	750G-4769G-7645C-16111T-16256T-16257T-146C-152C-263G-309.1C-309.2C-315.1C
H14	ARE	DB1G6	750G-4769G-7645C-16093C-16519C-263G-309.1C-309.2C-315.1C-524.1A-524.2C
H14	ARE	DB3A4	750G-4769G-7645C-16111T-16256T-16257T-146C-152C-263G-315.1C
H14	ARE	DB3B6	750G-4769G-7645C-16324C-146C-152C-263G-315.1C
H14	AUT	hm1F1	750G-4769G-7645C-16265T-263G-309.1C-315.1C-523DEL-524DEL
H14	AUT	hm2D8	750G-4769G-7645C-16265T-263G-309.1C-315.1C-523DEL-524DEL
H14	AUT	hm2F1	750G-4769G-7645C-16265T-263G-309.1C-315.1C-523DEL-524DEL
H14	AUT	hm3C6	750G-4769G-7645C-16265T-263G-309.1C-315.1C-523DEL-524DEL
H14	AUT	hm5B4	750G-4769G-7645C-16129R-16311C-16519C-195C-263G-309.1C-315.1C
H14	AUT	m1D5	750G-4769G-7645C-16129A-16311C-16519C-195C-263G-309.1C-315.1C
H14	MKD	MC2G12	750G-4769G-7645C-16311C-16519C-195C-263G-309.1C-315.1C
H14	MKD	MC3G2	750G-4769G-7645C-16311C-16390A-16519C-195C-263G-309.1C-315.1C
H14	ROU	SZ1D5	750G-4769G-7645C-16051G-16311C-16519C-195C-263G-309.1C-315.1C
H14a	AUT	hf4C3	750G-4769G-7645C-16189C-16256T-16278T-16352C-263G-315.1C-523DEL-524DEL
H14a	AUT	hm5E2	750G-4769G-7645C-16256T-16352C-263G-315.1C
H15	ARE	DB1C10	750G-4769G-6253C-16366T-55C-56T-57C-263G-309.1C-309.2C-315.1C-524.1A-524.2C
H15	ARE	DB3C11	750G-4769G-6253C-3847C-16189C-55C-57C-60.1T-146C-152C-263G-309.1C-309.2C-315.1C
H15	AUT	hf1B8	750G-4769G-6253C-16255A-55C-57C-263G-309.1C-309.2C-315.1C
H15	AUT	hf1C8	750G-4769G-6253C-55C-57C-65G-263G-309.1C-309.2C-315.1C
H15	AUT	hf1G10	750G-4769G-6253C-16311C-55C-57C-263G-309.1C-309.2C-315.1C
H15	AUT	hf2C10	750G-4769G-6253C-709A-57C-60.1T-263G-309.1C-309.2C-315.1C
H15	AUT	hf2C4	750G-4769G-6253C-16311C-55C-57C-263G-309.1C-309.2C-315.1C
H15	AUT	hf2E6	750G-4769G-6253C-16214T-16519C-55C-56G-56.1G-263G-309.1C-315.1C

H15	AUT	hf3D8	750G-4769G-6253C-16519C-55C-56G-56.1G-263G-309.1C-315.1C
H15	AUT	hf3G8	750G-4769G-6253C-55C-57C-65G-263G-309.1C-309.2C-315.1C
H15	AUT	hf3H10	750G-4769G-6253C-55C-57C-263G-309.1C-315.1C
H15	AUT	hf4E2	750G-4769G-6253C-55C-57C-263G-309.1C-309.2C-315.1C
H15	AUT	hm1B7	750G-4769G-6253C-55C-57C-263G-309.1C-309.2C-315.1C
H15	AUT	hm1E7	750G-4769G-6253C-16519C-55C-56G-56.1G-64T-263G-309.1C-315.1C-523DEL-524DEL
H15	AUT	hm1H8	750G-4769G-6253C-16519C-55C-56G-56.1G-64T-263G-309.1C-315.1C-523DEL-524DEL
H15	AUT	hm2B5	750G-4769G-6253C-55C-57C-263G-309.1C-309.2C-315.1C
H15	AUT	hm2C4	750G-4769G-6253C-55C-57C-263G-310C-314DEL-315DEL
H15	AUT	hm3G6	750G-4769G-6253C-16519C-55C-56G-56.1G-64T-263G-309.1C-315.1C-523DEL-524DEL
H15	AUT	hm6H8	750G-4769G-6253C-55C-57C-60.1T-152C-263G-309.1C-315.1C
H15	MKD	MC1E4	750G-4769G-6253C-3847C-4580A-55C-56.1A-263G-309.1C-309.2C-315.1C
H16	AUT	hf1B7	750G-4769G-10394T-16519C-152C-263G-309.1C-309.2C-315.1C
H16	AUT	hf1C1	750G-4769G-10394T-16291Y-16519C-152C-263G-315.1C
H16	AUT	hf3D3	750G-4769G-10394T-16189C-16519C-263G-315.1C
H16	AUT	hf3H3	750G-4769G-10394T-16189C-16519C-263G-315.1C
H16	AUT	hm1F7	750G-4769G-10394T-16183C-16189C-16519C-263G-315.1C
H16	AUT	hm2A10	750G-4769G-10394T-16519C-195Y-263G-309.1C-309.2C-315.1C
H16	AUT	hm2A11	750G-4769G-10394T-16519C-152C-263G-315.1C
H16	AUT	hm2H3	750G-4769G-10394T-16519C-152C-263G-309.1C-315.1C
H16	AUT	hm2H4	750G-4769G-10394T-16189C-16519C-263G-315.1C
H16	AUT	hm3A11	750G-4769G-10394T-16189C-16519C-263G-315.1C
H16	AUT	hm3B5	750G-4769G-10394T-16519C-152C-263G-309.1C-315.1C
H16	AUT	hm3F1	750G-4769G-10394T-16126C-16278T-16519C-150T-263G-315.1C
H16	AUT	hm3F4	750G-4769G-10394T-16126C-16278T-16519C-200R-263G-315.1C
H16	AUT	hm4G11	750G-4769G-10394T-16519C-152C-263G-309.1C-315.1C
H16	AUT	hm4H5	750G-4769G-10394T-16189C-16519C-263G-315.1C
H16	AUT	hm5F1	750G-4769G-10394T-16519C-152C-263G-309.1C-315.1C
H16	AUT	hm5F6	750G-4769G-10394T-16126C-16278T-16519C-263G-315.1C
H16	AUT	hm6G5	750G-4769G-10394T-16519C-152C-263G-315.1C
H17	ROU	CS1F7	750G-4769G-3915A-6296T-16129A-16189C-16193.1C-16193.2C-16519C-263G-309.1C-315.1C
H17	ROU	CS1H1	750G-4769G-3915A-6296T-16129A-16189C-16519C-263G-315.1C
H17	AUT	hf3H5	750G-4769G-3915A-16129A-16519C-263G-315.1C
H17	AUT	hm1D5	750G-4769G-3915A-16129A-16519C-263G-315.1C
H17	AUT	hm3G1	750G-4769G-3915A-16129A-16519C-263G-315.1C
H17	AUT	hm5E8	750G-4769G-3915A-6296T-16093C-16129A-16311C-16519C-150T-263G-315.1C
H17	ROU	SZ2D6	750G-4769G-3915A-6296T-16129R-16519C-263G-309.1C-315.1C
H1a	AUT	f3G3	750G-4769G-3010A-16051G-16162G-16291T-16304C-16519C-73G-263G-315.1C-515G
H1a	AUT	hf1C4	750G-4769G-3010A-16051G-16162G-16519C-73G-263G-315.1C

H1a	AUT	hf3A2	750G-4769G-3010A-16051G-16162G-16213A-16266T-16519C-73G-263G-309.1C-315.1C
H1a	AUT	hf3D6	750G-4769G-3010A-16051G-16162G-16380T-16519C-73G-263G-315.1C
H1a	AUT	hf3G2	750G-4769G-3010A-16162G-16519C-73G-263G-309.1C-315.1C
H1a	AUT	hf4F10	750G-4769G-3010A-16051G-16162G-16213A-16266Y-16311C-16519C-73G-263G-315.1C
H1a	AUT	hf4G5	750G-4769G-3010A-16051G-16162G-16519C-73G-263G-315.1C
H1a	AUT	hm1D11	750G-4769G-3010A-16051G-16162G-16213A-16266T-16519C-73G-263G-315.1C
H1a	AUT	hm1D6	750G-4769G-3010A-16162G-16519C-73G-263G-309.1C-315.1C
H1a	AUT	hm1H2	750G-4769G-3010A-16051G-16162G-16213A-16266T-16519C-73G-146C-263G-315.1C
H1a	AUT	hm2B4	750G-4769G-3010A-16051G-16162G-16291T-16304C-16519C-73G-263G-315.1C
H1a	AUT	hm2D10	750G-4769G-3010A-16051G-16162G-16266T-16362C-16519C-73G-263G-315.1C
H1a	AUT	hm2G5	750G-4769G-3010A-16162G-16325C-16519C-73G-263G-315.1C
H1a	AUT	hm3E10	750G-4769G-3010A-16162G-16519C-73G-152Y-263G-309.1C-315.1C
H1a	AUT	hm3F3	750G-4769G-3010A-16092C-16162G-16519C-73G-263G-309.1C-315.1C
H1a	AUT	hm3G8	750G-4769G-3010A-16051G-16162G-57G-73G-263G-315.1C
H1a	AUT	hm4E9	750G-4769G-3010A-16092C-16162G-16519C-73G-263G-309.1C-315.1C
H1a	AUT	hm4H12	750G-4769G-3010A-16051G-16162G-16291T-16304C-16519C-73G-263G-315.1C
H1a	AUT	hm5A8	750G-4769G-3010A-16162G-16320T-16519C-73G-263G-309.1C-315.1C
H1a	AUT	hm5B7	750G-4769G-3010A-16162G-16519C-73G-263G-309.1C-309.2C-315.1C
H1a	AUT	hm5D4	750G-4769G-3010A-16051G-16162G-16213A-16266T-16519C-73G-152Y-263G-309.1C-315.1C
H1a	AUT	hm5F10	750G-4769G-3010A-16051G-16162G-16291T-16304C-16519C-73G-263G-315.1C
H1a	AUT	hm5G12	750G-4769G-3010A-16051G-16162G-16519C-73G-217Y-263G-315.1C
H1a	AUT	hm5G6	750G-4769G-3010A-16051G-16162G-16278T-16519C-73G-263G-309.1C-315.1C
H1a	AUT	hm6C8	750G-4769G-3010A-16051G-16126C-16162G-16519C-73G-263G-315.1C
H1a	AUT	m1C2	750G-4769G-3010A-16162G-16519C-73G-263G-315.1C
H1a	AUT	m2D2	750G-4769G-3010A-16051G-16162G-16291T-16304C-16519C-73G-263G-315.1C
H1a	DEU	UL1D3	750G-4769G-3010A-16162G-16519C-73G-263G-315.1C
H1a1	ROU	CS1D1	750G-4769G-3010A-6365C-16162G-16172C-16209C-16266T-16519C-73G-200R-263G-315.1C
H1a1	ROU	CS1G3	750G-4769G-3010A-6365C-16162G-16172C-16209C-16266T-16519C-73G-263G-315.1C
H1a1	ROU	CS1H10	750G-4769G-3010A-6365C-16162G-16172C-16209C-16266T-16519C-73G-263G-315.1C
H1a1	ROU	CS2B5	750G-4769G-3010A-6365C-16162G-16172C-16209C-16266T-16519C-73G-263G-315.1C
H1a1	ROU	CS2D9	750G-4769G-3010A-6365C-16162G-16172C-16209C-16266T-16519C-73G-263G-315.1C
H1a1	ROU	CS2G4	750G-4769G-3010A-6365C-16162G-16172C-16209C-16266T-16519C-73G-263G-315.1C
H1a1	ROU	CS2H7	750G-4769G-3010A-6365C-16162G-16172C-16209C-16266T-16519C-73G-263G-315.1C
H1a1	AUT	f2C4	750G-4769G-3010A-6365C-16162G-16209C-16519C-73G-263G-315.1C
H1a1	AUT	f3G4	750G-4769G-3010A-6365C-16162G-16519C-73G-263G-315.1C
H1a1	AUT	hf3D5	750G-4769G-3010A-6365C-16162G-16209C-16519C-73G-263G-315.1C
H1a1	AUT	hf3G11	750G-4769G-3010A-6365C-16162G-16209C-16519C-73G-263G-309.1C-315.1C
H1a1	AUT	hf3H4	750G-4769G-3010A-6365C-16162G-16209C-16519C-73G-263G-309.1C-315.1C
H1b	HUN	As1A3	750G-4769G-3010A-16080G-16183C-16189C-16193.1C-16356C-16360T-183G-263G-309.1C-309.2C-315.1C

H1b	HUN	As1F4	750G-4769G-3010A-16080G-16183C-16189C-16193.1C-16356C-16360T-183G-263G-309.1C-315.1C
H1b	HUN	As2C1	750G-4769G-3010A-16080G-16183C-16189C-16193.1C-16356C-16360T-183G-263G-309.1C-309.2C-315.1C
H1b	HUN	As2D1	750G-4769G-3010A-16189C-16356C-16519C-152C-263G-309.1C-315.1C
H1b	HUN	As2E6	750G-4769G-3010A-16189C-16356C-16519C-152C-263G-309.1C-309.2C-315.1C
H1b	HUN	As2E7	750G-4769G-3010A-16189C-16356C-16519C-152C-263G-309.1C-315.1C
H1b	HUN	As2H10	750G-4769G-3010A-16080G-16129R-16183C-16189C-16193.1C-16356C-16360T-183G-263G-309.1C-309.2C-309.3C-315.1C
H1b	AUT	f1D4	750G-4769G-3010A-3796G-16189C-16356C-16362C-16519C-263G-309.1C-315.1C-523DEL-524DEL
H1b	AUT	f1E4	750G-4769G-3010A-3796G-16189C-16356C-16362C-16519C-263G-309.1C-315.1C-523DEL-524DEL
H1b	AUT	f2C6	750G-4769G-3010A-3796G-16189C-16356C-16362C-16519C-263G-315.1C-523DEL-524DEL
H1b	AUT	f2E1	750G-4769G-3010A-3796G-16189C-16356C-16362C-16519C-263G-309.1C-309.2C-315.1C-523DEL-524DEL
H1b	AUT	hf1H8	750G-4769G-3010A-3796G-16189C-16356C-16362C-16519C-263G-315.1C-523DEL-524DEL
H1b	AUT	hf2A9	750G-4769G-3010A-3796G-16148T-16189C-16356C-16519C-151T-152Y-263G-315.1C-523DEL-524DEL
H1b	AUT	hf2E1	750G-4769G-3010A-16183C-16189C-16193.1C-16356C-16519C-152C-263G-309.1C-309.2C-315.1C
H1b	AUT	hf2G5	750G-4769G-3010A-3796G-16189C-16356C-16362C-16519C-263G-309.1C-309.2C-315.1C-523DEL-524DEL
H1b	AUT	hf2G7	750G-4769G-3010A-3796G-16189C-16356C-16362C-16519C-263G-315.1C-523DEL-524DEL
H1b	AUT	hf3F3	750G-4769G-3010A-3796G-16189C-16356C-16362C-16519C-263G-315.1C-374G-523DEL-524DEL
H1b	AUT	hf4A6	750G-4769G-3010A-3796G-16183C-16189C-16193.1C-16356C-16519C-56.1T-57C-263G-309.1C-309.2C-315.1C-523DEL-524DEL
H1b	AUT	hf4C10	750G-4769G-3010A-16189C-16356C-16519C-263G-315.1C
H1b	AUT	hf4C8	750G-4769G-3010A-3796G-16189C-16356C-16362C-16519C-263G-315.1C-523DEL-524DEL-573.1C-573.2C
H1b	AUT	hf4E8	750G-4769G-3010A-3796G-16129A-16189C-16193.1C-16193.2C-16355T-16356C-16362C-16519C-263G-309.1C-309.2C-315.1C-523DEL-524DEL
H1b	AUT	hf4F9	750G-4769G-3010A-3796G-16189C-16193.1C-16193.2C-16356C-16362C-16519C-263G-315.1C-374G-523DEL-524DEL
H1b	AUT	hf4G12	750G-4769G-3010A-16189C-16356C-16519C-263G-315.1C
H1b	AUT	hf4H7	750G-4769G-3010A-3796G-16189C-16215G-16356C-16362C-16519C-263G-309.1C-309.2C-315.1C-523DEL-524DEL
H1b	AUT	hm1C11	750G-4769G-3010A-3796G-16189C-16356C-16362C-16519C-263G-315.1C-523DEL-524DEL
H1b	AUT	hm1C8	750G-4769G-3010A-3796G-16172C-16189C-16356C-16362C-16519C-263G-315.1C-523DEL-524DEL
H1b	AUT	hm1E8	750G-4769G-3010A-3796G-16189C-16356C-16362C-16519C-263G-309.1C-315.1C-523DEL-524DEL
H1b	AUT	hm1E9	750G-4769G-3010A-3796G-16189C-16356C-16362C-16519C-263G-315.1C-523DEL-524DEL-573.1C-573.2C
H1b	AUT	hm2A4	750G-4769G-3010A-3796G-16189C-16356C-16362C-16519C-263G-315.1C-523DEL-524DEL
H1b	AUT	hm2C1	750G-4769G-3010A-3796G-16189C-16356C-16519C-152C-263G-315.1C-523DEL-524DEL
H1b	AUT	hm2D1	750G-4769G-3010A-3796G-16189C-16356C-16362C-16519C-263G-315.1C-523DEL-524DEL
H1b	AUT	hm3E11	750G-4769G-3010A-3796G-16189C-16356C-16362C-16519C-263G-315.1C-523DEL-524DEL
H1b	AUT	hm4A11	750G-4769G-3010A-3796G-16189C-16356C-16362C-16519C-263G-309.1C-309.2C-315.1C-523DEL-524DEL
H1b	AUT	hm4A4	750G-4769G-3010A-16080G-16189C-16223T-16356C-151T-263G-309.1C-309.2C-315.1C
H1b	AUT	hm4D4	750G-4769G-3010A-16183C-16189C-16193.1C-16356C-16519C-16527T-263G-309.1C-309.2C-315.1C
H1b	AUT	hm5D9	750G-4769G-3010A-3796G-16189C-16356C-16362C-16519C-263G-309.1C-315.1C-523DEL-524DEL
H1b	AUT	hm5E11	750G-4769G-3010A-3796G-16179T-16189C-16356C-16362C-16519C-263G-315.1C-523DEL-524DEL
H1b	AUT	hm5F8	750G-4769G-3010A-3796G-16169T-16189C-16260T-16356C-16362C-16519C-263G-309.1C-309.2C-315.1C-523DEL-524DEL
H1b	AUT	hm5G10	750G-4769G-3010A-3796G-16189C-16356C-16362C-16519C-263G-315.1C-523DEL-524DEL

H1b	AUT	hm6D8	750G-4769G-3010A-3796G-16129A-16189C-16193.1C-16193.2C-16355T-16356C-16362C-16519C-263G-309.1C-309.2C-315.1C-523DEL-524DEL
H1b	AUT	hm6F3	750G-4769G-3010A-16189C-16193.1C-16356C-16519C-152C-263G-309.1C-309.2C-315.1C
H1b	AUT	hm6H2	750G-4769G-3010A-3796G-16189C-16356C-16362C-16519C-263G-315.1C-374G-523DEL-524DEL
H1b	AUT	m2D4	750G-4769G-3010A-3796G-16183C-16189C-16356C-16362C-16519C-263G-315.1C-523DEL-524DEL
H1b	MKD	MC1D5	750G-4769G-3010A-16189C-16356C-16519C-263G-309.1C-309.2C-315.1C
H1b	MKD	MC2B10	750G-4769G-3010A-16189C-16324C-16356C-16519C-152C-263G-315.1C
H1b	MKD	MC2C7	750G-4769G-3010A-3796G-16183C-16189C-16193.1C-16356C-16362C-16519C-93G-263G-309.1C-309.2C-315.1C-523DEL-524DEL
H1b	ROU	SZ1C1	750G-4769G-3010A-3796G-16189C-16193.1C-16356C-16362C-16519C-263G-315.1C-523DEL-524DEL
H1b	ROU	SZ1F10	750G-4769G-3010A-3796G-16129A-16189C-16193.1C-16355T-16356C-16362C-16519C-263G-309.1C-309.2C-315.1C-523DEL-524DEL
H1b	ROU	SZ1G5	750G-4769G-3010A-3796G-16129A-16189C-16193.1C-16355T-16356C-16362C-16519C-263G-309.1C-309.2C-315.1C-523DEL-524DEL
H1b	ROU	SZ2A12	750G-4769G-3010A-3796G-16129A-16189C-16193.1C-16355T-16356C-16362C-16519C-263G-309.1C-315.1C-523DEL-524DEL
H1b	ROU	SZ2C7	750G-4769G-3010A-3796G-16189C-16356C-16362C-16519C-263G-315.1C-523DEL-524DEL
H1b	DEU	UL1G11	750G-4769G-3010A-3796G-16189C-16356C-16362C-16519C-263G-315.1C-523DEL-524DEL
H1b	DEU	UL2H1	750G-4769G-3010A-3796G-16183C-16189C-16193.1C-16356C-16519C-146C-263G-309.1C-309.2C-315.1C
H1c	AUT	f1C5	750G-4769G-3010A-263G-309.1C-315.1C-477C
H1c	AUT	f1C6	750G-4769G-3010A-16278Y-16519C-263G-315.1C-477C
H1c	AUT	hf1C11	750G-4769G-3010A-16519C-263G-315.1C-477C
H1c	AUT	hf1H4	750G-4769G-3010A-16519C-152Y-263G-315.1C-477C
H1c	AUT	hf2B7	750G-4769G-3010A-263G-309.1C-315.1C-477C
H1c	AUT	hf2F10	750G-4769G-3010A-263G-309.1C-315.1C-477C
H1c	AUT	hf2G10	750G-4769G-3010A-16519C-263G-315.1C-477C
H1c	AUT	hf2H12	750G-4769G-3010A-16129A-16519C-263G-315.1C-477C
H1c	AUT	hf2H6	750G-4769G-3010A-263G-309.1C-315.1C-477C
H1c	AUT	hf3A4	750G-4769G-3010A-263G-309.1C-315.1C-477C
H1c	AUT	hf3E7	750G-4769G-3010A-263G-309.1C-315.1C-477C
H1c	AUT	hf3F4	750G-4769G-3010A-16519C-263G-315.1C-477C
H1c	AUT	hf3G1	750G-4769G-3010A-263G-309.1C-315.1C-477C
H1c	AUT	hf3G5	750G-4769G-3010A-263G-309.1C-315.1C-477C
H1c	AUT	hf4A1	750G-4769G-3010A-16218T-263G-309.1C-315.1C-477C
H1c	AUT	hf4A10	750G-4769G-3010A-263G-309.1C-315.1C-477C
H1c	AUT	hf4D4	750G-4769G-3010A-263G-309.1C-309.2C-315.1C-477C
H1c	AUT	hf4F3	750G-4769G-3010A-16129A-16519C-263G-309.1C-315.1C-477C
H1c	AUT	hf4F6	750G-4769G-3010A-16519C-263G-315.1C-477C
H1c	AUT	hf4H9	750G-4769G-3010A-16240R-263G-309.1C-315.1C-477C
H1c	AUT	hm1A8	750G-4769G-3010A-16240R-263G-309.1C-315.1C-477C
H1c	AUT	hm1B11	750G-4769G-3010A-16129A-16519C-93G-263G-315.1C-477C
H1c	AUT	hm1C1	750G-4769G-3010A-263G-309.1C-315.1C-477C
H1c	AUT	hm1G8	750G-4769G-3010A-8473C-16519C-195C-257G-263G-309.1C-315.1C-477C

H1c	AUT	hm2B7	750G-4769G-3010A-16042A-16288C-16519C-263G-315.1C-477C
H1c	AUT	hm2C2	750G-4769G-3010A-16042A-16288C-16519C-263G-315.1C-477C
H1c	AUT	hm2D7	750G-4769G-3010A-16042A-16288C-16519C-263G-315.1C-477C
H1c	AUT	hm2E5	750G-4769G-3010A-72C-263G-315.1C-477C
H1c	AUT	hm2G1	750G-4769G-263G-315.1C-477C
H1c	AUT	hm2G9	750G-4769G-3010A-263G-309.1C-315.1C-477C
H1c	AUT	hm3A10	750G-4769G-3010A-16519C-263G-309.1C-309.2C-315.1C-477C
H1c	AUT	hm3B2	750G-4769G-3010A-16042A-16288C-16519C-263G-315.1C-477C
H1c	AUT	hm3E4	750G-4769G-3010A-16189C-16519C-263G-309.1C-315.1C-477C
H1c	AUT	hm3H3	750G-4769G-3010A-16519C-263G-309.1C-315.1C-477C-523DEL-524DEL
H1c	AUT	hm4A7	750G-4769G-3010A-263G-315.1C-477C
H1c	AUT	hm4A8	750G-4769G-3010A-263G-315.1C-477C
H1c	AUT	hm4G2	750G-4769G-3010A-263G-309.1C-315.1C-477C
H1c	AUT	hm5C7	750G-4769G-3010A-16519C-263G-315.1C-477C
H1c	AUT	hm6A5	750G-4769G-3010A-263G-309.1C-315.1C-477C
H1c	AUT	hm6D2	750G-4769G-3010A-263G-309.1C-315.1C-477C
H1c	AUT	hm6G8	750G-4769G-3010A-16042A-16288C-16519C-263G-315.1C-477C
H1c	AUT	m2B2	750G-4769G-3010A-16042A-16288C-16519C-263G-315.1C-477C
H1c	ROU	SZ1G1	750G-4769G-3010A-16519C-263G-315.1C-477C
H1c	ROU	SZ2B10	750G-4769G-3010A-16311C-16519C-263G-309.1C-309.2C-315.1C-477C
H1c	ROU	SZ2E9	750G-4769G-3010A-16519C-263G-315.1C-477C
H1c	ROU	SZ2G9	750G-4769G-3010A-16519C-263G-315.1C-477C
H1c1	AUT	f1C2	750G-4769G-3010A-9150G-16093C-16263C-16311C-16390A-16519C-263G-315.1C-477C
H1c1	AUT	f3C6	750G-4769G-3010A-9150G-16263C-16519C-263G-315.1C-477C
H1c1	AUT	hf1D8	750G-4769G-3010A-9150G-16093C-16263C-16311C-16519C-263G-309.1C-315.1C-477C
H1c1	AUT	hf2B4	750G-4769G-3010A-9150G-16093C-16263C-16311C-16519C-263G-315.1C-477C
H1c1	AUT	hf3E9	750G-4769G-3010A-9150G-16093C-16263C-16519C-263G-315.1C-477C
H1c1	AUT	hf3H7	750G-4769G-3010A-9150G-16093C-16263C-16311C-16519C-263G-315.1C-477C-573.1C
H1c1	AUT	hf4C1	750G-4769G-3010A-9150G-16093C-16263C-16311C-16519C-263G-315.1C-477C
H1c1	AUT	hm1B2	750G-4769G-3010A-9150G-16093C-16263C-16519C-263G-309.1C-315.1C-477C
H1c1	AUT	hm1D1	750G-4769G-3010A-9150G-16093C-16263C-16311C-16519C-263G-309.1C-315.1C-477C
H1c1	AUT	hm2D5	750G-4769G-3010A-9150G-16263C-16316G-16519C-263G-315.1C-477C
H1c1	AUT	hm2G4	750G-4769G-3010A-9150G-16093C-16263C-16311C-16390A-16519C-263G-309.1C-315.1C-477C
H1c1	AUT	hm3B4	750G-4769G-3010A-9150G-16093C-16263C-16519C-263G-315.1C-477C
H1c1	AUT	hm3F6	750G-4769G-3010A-9150G-16263C-16519C-263G-309.1C-315.1C-477C
H1c1	AUT	hm3G7	750G-4769G-3010A-9150G-16093C-16263C-16519C-263G-309.1C-309.2C-315.1C-477C
H1c1	AUT	hm3H7	750G-4769G-3010A-9150G-16093C-16263C-16519C-263G-309.1C-315.1C-477C
H1c1	AUT	hm5D10	750G-4769G-3010A-9150G-16093C-16263C-16311C-16519C-263G-315.1C-477C
H1c1	AUT	hm6E3	750G-4769G-3010A-9150G-16093Y-16263C-16519C-263G-315.1C-477C

H1c1	AUT	m3E6	750G-4769G-3010A-9150G-16093C-16263C-16519C-263G-315.1C-477C
H1c2	AUT	hf4H3	750G-4769G-3010A-12858T-16519C-263G-315.1C-477C
H1c2	AUT	hm3H4	750G-4769G-3010A-12858T-16519C-263G-315.1C-477C
H1c2	AUT	hm4G4	750G-4769G-3010A-12858T-16519C-263G-315.1C-477C
H1c2	AUT	hm5C2	750G-4769G-3010A-12858T-16519C-263G-315.1C-477C
H1c2	AUT	hm5E4	750G-4769G-3010A-12858T-16519C-263G-315.1C-477C
H1c2	AUT	hm6C5	750G-4769G-3010A-12858T-16519C-263G-315.1C-477C
H1c2	AUT	m3C5	750G-4769G-3010A-12858T-16519C-263G-315.1C-477C
H1f	AUT	hm4G1	750G-4769G-3010A-9066G-709A-16189C-16519C-263G-315.1C
H1f	AUT	hm5H4	750G-4769G-3010A-9066G-16093C-16189C-16519C-93G-263G-309.1C-315.1C
H1f	AUT	hm6H4	750G-4769G-3010A-9066G-16093C-16189C-16519C-263G-309.1C-315.1C-523DEL-524DEL
H21	AUT	hm3G10	750G-4769G-16192T-186A-263G-315.1C
H2a	AUT	hf2H5	750G-315.1C-456T
H2a	AUT	hf3C9	750G-16266T-16311C-16399G-16519C-263G-315.1C-523DEL-524DEL
H2a	AUT	hf4B2	750G-16291T-193G-263G-309.1C-315.1C
H2a	AUT	hm1H6	750G-263G-315.1C-518T
H2a	AUT	hm2F11	750G-263G-315.1C-518T
H2a	AUT	hm4A5	750G-73G-263G-315.1C
H2a	AUT	hm4G6	750G-16291T-193G-263G-309.1C-309.2C-315.1C
H2a	AUT	hm6C3	750G-73G-263G-315.1C
H2a	AUT	m1H1	750G-16291T-193G-263G-315.1C
H2a1	AUT	f2D5	750G-951A-16354T-152C-194T-263G-309.1C-315.1C
H2a1	AUT	hf1G1	750G-951A-16354T-263G-315.1C-575T
H2a1	AUT	hf1G12	750G-951A-16354T-152C-263G-315.1C-523DEL-524DEL
H2a1	AUT	hf1G4	750G-951A-16093C-16354T-263G-293C-315.1C
H2a1	AUT	hf1G7	750G-951A-16181G-16354T-16519C-263G-309.1C-315.1C
H2a1	AUT	hf2C1	750G-951A-16354T-16519C-263G-309.1C-309.2C-315.1C
H2a1	AUT	hf2F7	750G-951A-16354T-152C-194T-263G-309.1C-315.1C
H2a1	AUT	hf2H7	750G-951A-16354T-16519C-263G-309.1C-309.2C-315.1C
H2a1	AUT	hf3B3	750G-951A-16354T-152C-263G-309.1C-315.1C
H2a1	AUT	hf3C5	750G-951A-16354T-263G-309.1C-315.1C
H2a1	AUT	hf3F10	750G-951A-16519C-73G-195C-263G-309.1C-315.1C
H2a1	AUT	hf4D5	750G-951A-16354T-263G-309.1C-315.1C
H2a1	AUT	hm1A1	750G-951A-16354T-263G-309.1C-309.2C-315.1C
H2a1	AUT	hm2E1	750G-951A-16181G-16354T-16519C-263G-309.1C-315.1C
H2a1	AUT	hm3A3	750G-951A-16093C-16354T-263G-293C-315.1C
H2a1	AUT	hm3C8	750G-951A-263G-309.1C-315.1C-518T
H2a1	AUT	hm3G12	750G-951A-16354T-263G-293C-315.1C-523DEL-524DEL
H2a1	AUT	hm4C6	750G-951A-16354T-16519C-263G-309.1C-309.2C-315.1C

H2a1	AUT	hm5A10	750G-951A-16354T-263G-293C-315.1C-523DEL-524DEL
H2a1	AUT	hm5B8	750G-951A-16354T-146C-263G-315.1C
H2a1	AUT	hm5F5	750G-951A-16354T-263G-309.1C-315.1C-444G
H2a1	AUT	hm5G5	750G-951A-16354T-263G-309.1C-315.1C-444G
H2a1	AUT	hm5H7	750G-951A-16354T-152C-194T-263G-309.1C-315.1C
H2a1	MKD	MC1A3	750G-951A-16354T-146C-263G-315.1C
H2a1	MKD	MC1E2	750G-951A-16354T-16519C-152C-263G-309.1C-315.1C
H2a2	HUN	As2D9	93G-309.1C-315.1C-523DEL-524DEL
H2a2	AUT	f3E1	309.1C-315.1C
H2a2	AUT	hf1F3	16154Y-309.1C-315.1C
H2a2	AUT	hf1F6	16293G-309.1C-315.1C-456T-459.1C
H2a2	AUT	hf2B10	315.1C
H2a2	AUT	hf2H10	
H2a2	AUT	hf3D2	16126Y
H2a2	AUT	hf4A11	315.1C
H2a2	AUT	hf4D11	195C-309.1C-315.1C
H2a2	AUT	hf4G9	315.1C
H2a2	AUT	hm1A2	93G
H2a2	AUT	hm1E2	
H2a2	AUT	hm1F2	
H2a2	AUT	hm2C3	263G-309.1C-315.1C
H2a2	AUT	hm2G12	16316G-143A-315.1C
H2a2	AUT	hm3G9	315.1C-456T
H2a2	AUT	hm3H9	16270T-315.1C
H2a2	AUT	hm4B8	309.1C-315.1C
H2a2	AUT	hm4C5	16235G-16291T-16293G-16400T-263G-309.1C-309.2C-315.1C
H2a2	AUT	hm4F5	152Y-309.1C-315.1C
H2a2	AUT	hm5C1	315.1C-456T
H2a2	AUT	hm5G3	16235G-16291T-16293G-16400T-263G-309.1C-315.1C
H2a2	AUT	hm6F6	
H2a2	AUT	hm6G4	309.1C-315.1C
H2a3	AUT	hf1A3	750G-10810C-16274A-16519C-263G-315.1C
H2a3	AUT	hm4G8	750G-10810C-16227G-16274A-16519C-146C-263G-309.1C-315.1C
H2a3	AUT	hm6A8	750G-10810C-16274A-16519C-263G-315.1C
H3	HUN	As1H4	750G-4769G-6776C-16222T-16299G-16519C-146C-263G-309.1C-309.2C-315.1C
H3	HUN	As1H6	750G-4769G-6776C-16248T-16519C-263G-309.1C-315.1C
H3	HUN	As2B6	750G-4769G-6776C-16248T-16519C-263G-309.1C-309.2C-315.1C
H3	HUN	As2G12	750G-4769G-6776C-16248T-16519C-263G-309.1C-309.2C-315.1C
H3	AUT	f2E4	750G-4769G-6776C-16519C-263G-309.1C-315.1C

H3	AUT	f2G1	750G-4769G-6776C-16311C-16519C-152C-263G-315.1C
H3	AUT	f3B1	750G-4769G-6776C-16519C-152C-263G-315.1C-524.1A-524.2C
H3	AUT	f3B6	750G-4769G-6776C-16519C-263G-309.1C-309.2C-315.1C
H3	AUT	hf1A1	750G-4769G-6776C-16311C-16519C-263G-315.1C-523DEL-524DEL
H3	AUT	hf1A10	750G-4769G-6776C-16519C-263G-309.1C-309.2C-315.1C
H3	AUT	hf1C2	750G-4769G-6776C-16080G-16519C-263G-309.1C-309.2C-315.1C
H3	AUT	hf1D2	750G-4769G-6776C-16298C-16352C-16519C-73G-263G-315.1C
H3	AUT	hf1E11	750G-4769G-6776C-16129A-16519C-263G-315.1C
H3	AUT	hf1E2	750G-4769G-6776C-16298C-16352C-16519C-73G-263G-315.1C
H3	AUT	hf1E9	750G-4769G-6776C-16129A-16519C-263G-315.1C
H3	AUT	hf1F8	750G-4769G-6776C-16264T-16311C-16519C-263G-315.1C
H3	AUT	hf1H5	750G-4769G-6776C-16129A-16519C-263G-315.1C
H3	AUT	hf2A3	750G-4769G-6776C-16192T-16311C-16519C-93G-263G-315.1C
H3	AUT	hf2B5	750G-4769G-6776C-16519C-263G-309.1C-309.2C-315.1C
H3	AUT	hf2C8	750G-4769G-6776C-16129A-16519C-263G-315.1C
H3	AUT	hf2D1	750G-4769G-6776C-16519C-152C-263G-315.1C
H3	AUT	hf2F1	750G-4769G-6776C-16519C-263G-315.1C
H3	AUT	hf2G11	750G-4769G-6776C-16263C-16266T-16311C-16519C-152Y-263G-315.1C
H3	AUT	hf3A10	750G-4769G-6776C-16519C-263G-293C-315.1C
H3	AUT	hf3A5	750G-4769G-6776C-16093C-16263C-16266T-16311C-16519C-263G-315.1C
H3	AUT	hf3B11	750G-4769G-6776C-14470A-16093C-16519C-195C-263G-309.1C-309.2C-315.1C-408A
H3	AUT	hf3F7	750G-4769G-6776C-16129A-16519C-263G-315.1C
H3	AUT	hf4A9	750G-4769G-6776C-16093C-16263C-16266T-16311C-16519C-263G-315.1C
H3	AUT	hf4G10	750G-4769G-6776C-16519C-152C-217Y-263G-315.1C
H3	AUT	hm1A5	750G-4769G-6776C-16519C-263G-315.1C
H3	AUT	hm1B9	750G-4769G-6776C-16519C-263G-309.1C-309.2C-315.1C
H3	AUT	hm1C10	750G-4769G-6776C-16129A-16519C-263G-315.1C
H3	AUT	hm1D9	750G-4769G-6776C-16129A-16519C-263G-315.1C
H3	AUT	hm1F4	750G-4769G-6776C-16519C-152C-263G-315.1C
H3	AUT	hm1G7	750G-4769G-6776C-16093C-16519C-263G-315.1C-408A
H3	AUT	hm1H4	750G-4769G-6776C-16519C-263G-309.1C-315.1C
H3	AUT	hm2B2	750G-4769G-6776C-16298C-16352C-16519C-73G-263G-315.1C
H3	AUT	hm2E11	750G-4769G-6776C-16311C-16519C-152C-263G-315.1C
H3	AUT	hm2E9	750G-4769G-6776C-16172C-16362C-16519C-73G-143A-263G-309.1C-315.1C
H3	AUT	hm2F5	750G-4769G-6776C-16519C-152C-263G-315.1C
H3	AUT	hm2F6	750G-4769G-6776C-16129A-16519C-263G-315.1C
H3	AUT	hm2F7	750G-4769G-6776C-16129A-16519C-263G-315.1C
H3	AUT	hm2G2	750G-4769G-6776C-16129A-16519C-263G-315.1C
H3	AUT	hm3B6	750G-4769G-6776C-16311C-16519C-152C-263G-315.1C

H3	AUT	hm3D8	750G-4769G-6776C-16519C-263G-309.1C-315.1C
H3	AUT	hm3D9	750G-4769G-6776C-16519C-93G-263G-309.1C-315.1C
H3	AUT	hm3E9	750G-4769G-6776C-16298C-16352C-16519C-73G-263G-315.1C
H3	AUT	hm3F10	750G-4769G-6776C-16519C-152C-263G-315.1C
H3	AUT	hm3F2	750G-4769G-6776C-16298C-16352C-16519C-73G-263G-315.1C
H3	AUT	hm3F5	750G-4769G-6776C-16129A-16519C-263G-315.1C
H3	AUT	hm4A9	750G-4769G-6776C-16519C-263G-309.1C-309.2C-315.1C
H3	AUT	hm4B10	750G-4769G-6776C-16129A-16519C-263G-315.1C
H3	AUT	hm4C8	750G-4769G-6776C-16129A-16519C-263G-315.1C
H3	AUT	hm4D9	750G-4769G-6776C-16311C-16519C-263G-309.1C-315.1C
H3	AUT	hm4F3	750G-4769G-6776C-16519C-200G-263G-309.1C-315.1C
H3	AUT	hm4G10	750G-4769G-6776C-16519C-263G-315.1C
H3	AUT	hm4H8	750G-4769G-6776C-16311C-16519C-152C-263G-309.1C-315.1C
H3	AUT	hm5D2	750G-4769G-6776C-16042A-16519C-152C-263G-315.1C
H3	AUT	hm5D8	750G-4769G-6776C-16129A-16519C-263G-315.1C
H3	AUT	hm5G8	750G-4769G-6776C-16519C-263G-309.1C-309.2C-315.1C
H3	AUT	hm6A1	750G-4769G-6776C-16519C-152C-263G-315.1C
H3	AUT	hm6C2	750G-4769G-6776C-16519C-152C-263G-309.1C-315.1C
H3	AUT	hm6C6	750G-4769G-6776C-16519C-152C-263G-315.1C
H3	AUT	hm6G2	750G-4769G-6776C-16299G-16519C-150T-263G-315.1C
H3	AUT	m1A4	750G-4769G-6776C-16299G-16519C-150T-263G-315.1C
H3	AUT	m1A5	750G-4769G-6776C-16519C-263G-309.1C-309.2C-315.1C
H3	MKD	MC2D7	750G-4769G-6776C-16311C-16519C-263G-315.1C-523DEL-524DEL
H3	DEU	UL1A3	750G-4769G-6776C-16093C-16519C-242T-263G-315.1C-408A
H3	DEU	UL1A5	750G-4769G-6776C-16519C-185A-263G-315.1C
H3b	AUT	hf1B1	750G-4769G-6776C-2581G-16129A-16519C-263G-315.1C
H3b	AUT	hf2B11	750G-4769G-6776C-2581G-16086C-16129A-16362Y-16519C-263G-315.1C
H3b	AUT	hf2F9	750G-4769G-6776C-2581G-16111T-16129A-16256T-16519C-93G-153G-263G-309.1C-309.2C-315.1C
H3b	AUT	hf4F2	750G-4769G-6776C-2581G-16129A-16266T-16293G-16519C-263G-315.1C
H3b	AUT	hm1E3	750G-4769G-6776C-2581G-16111T-16129A-16256T-16519C-93G-153G-263G-309.1C-309.2C-315.1C
H3b	AUT	hm4D6	750G-4769G-6776C-2581G-16086C-16129A-16519C-263G-315.1C
H3c	AUT	hm6B3	750G-4769G-6776C-12957C-16278T-16519C-260A-263G-309.1C-315.1C
H4	AUT	f1G2	750G-4769G-3992T-16311C-263G-309.1C-315.1C-523DEL-524DEL
H4	AUT	hf1G8	750G-4769G-3992T-2581G-263G-315.1C-523DEL-524DEL
H4	AUT	hf2D7	750G-4769G-3992T-16311C-263G-309.1C-315.1C-523DEL-524DEL
H4	AUT	hf2F2	750G-4769G-3992T-16248T-16519C-73G-187T-263G-315.1C-523DEL-524DEL
H4	AUT	hf3B8	750G-4769G-3992T-16145A-185A-263G-315.1C-523DEL-524DEL-573.1C
H4	AUT	hm4E7	750G-4769G-3992T-263G-315.1C-523DEL-524DEL
H4	AUT	hm6D6	750G-4769G-3992T-16265W-263G-315.1C-523DEL-524DEL

H4	MKD	MC2C3	750G-4769G-3992T-263G-315.1C-523DEL-524DEL
H4	MKD	MC2G5	750G-4769G-3992T-16519C-263G-315.1C-523DEL-524DEL
H4	MKD	MC3G1	750G-4769G-3992T-263G-315.1C-523DEL-524DEL
H4a	AUT	f1B3	750G-4769G-3992T-14365T-263G-315.1C-340T-523DEL-524DEL
H4a	AUT	f2G2	750G-4769G-3992T-14365T-263G-309.1C-309.2C-315.1C-523DEL-524DEL
H4a	AUT	f3C4	750G-4769G-3992T-14365T-263G-315.1C-523DEL-524DEL
H4a	AUT	hf1A5	750G-4769G-3992T-14365T-263G-315.1C-523DEL-524DEL
H4a	AUT	hf1E1	750G-4769G-3992T-14365T-263G-315.1C-340T-523DEL-524DEL
H4a	AUT	hf2C7	750G-4769G-3992T-14365T-263G-315.1C-340T-523DEL-524DEL
H4a	AUT	hf2E5	750G-4769G-3992T-14365T-263G-309.1C-315.1C-523DEL-524DEL
H4a	AUT	hm1C3	750G-4769G-3992T-14365T-16311C-263G-309.1C-315.1C-523DEL-524DEL
H4a	AUT	hm1F9	750G-4769G-3992T-14365T-263G-315.1C-340T-523DEL-524DEL
H4a	AUT	m3F1	750G-4769G-3992T-14365T-263G-315.1C-523DEL-524DEL
H4a	MKD	MC2B8	750G-4769G-3992T-14365T-16319A-263G-315.1C-523DEL-524DEL
H4a	ROU	SZ1E9	750G-4769G-3992T-14365T-263G-315.1C-523DEL-524DEL
H4a1	AUT	hm2E2	750G-4769G-3992T-14365T-8269A-263G-309.1C-309.2C-315.1C-523DEL-524DEL
H4a1a	HUN	As1D11	750G-4769G-3992T-14365T-8269A-10044G-73G-152C-263G-309.1C-309.2C-315.1C-523DEL-524DEL
H4a1a	HUN	As1D2	750G-4769G-3992T-14365T-8269A-10044G-73G-152C-263G-309.1C-315.1C-523DEL-524DEL
H4a1a	HUN	As1F2	750G-4769G-3992T-14365T-8269A-10044G-73G-152C-263G-309.1C-315.1C-523DEL-524DEL
H4a1a	AUT	hf3F6	750G-4769G-3992T-14365T-8269A-10044G-73G-263G-315.1C-523DEL-524DEL
H4a1a	AUT	hm1A10	750G-4769G-3992T-14365T-8269A-10044G-16274A-73G-263G-315.1C-523DEL-524DEL
H4a1a	AUT	hm1B10	750G-4769G-3992T-14365T-8269A-10044G-16274A-73G-263G-315.1C-523DEL-524DEL
H4a1a	AUT	hm3D7	750G-4769G-3992T-14365T-8269A-10044G-16274A-73G-263G-315.1C-523DEL-524DEL
H4a1a	AUT	hm6B2	750G-4769G-3992T-14365T-8269A-10044G-73G-182T-263G-309.1C-309.2C-315.1C-523DEL-524DEL
H4a1a	AUT	m3E4	750G-4769G-3992T-14365T-8269A-10044G-73G-182T-263G-309.1C-315.1C-523DEL-524DEL
H4a1a	MKD	MC2B9	750G-4769G-3992T-14365T-8269A-10044G-73G-263G-315.1C-523DEL-524DEL
H5	ROU	CS1C8	750G-4769G-16304C-189G-200G-263G-309.1C-315.1C-456T
H5	ROU	CS1D11	750G-4769G-16304C-189G-200G-263G-309.1C-315.1C-456T
H5	ROU	CS1G11	750G-4769G-16304C-16318G-263G-309.1C-315.1C-456T
H5	ROU	CS1G8	750G-4769G-16304C-16318G-263G-309.1C-315.1C-456T
H5	ROU	CS2B1	750G-4769G-16189C-16193.1C-16294T-16304C-16527T-263G-315.1C-456T
H5	ROU	CS2C11	750G-4769G-16304C-189G-200G-263G-309.1C-315.1C-456T
H5	ROU	CS2C6	750G-4769G-16304C-16318G-263G-309.1C-315.1C-456T
H5	ARE	DB2H7	750G-4769G-16294T-16304C-152C-263G-315.1C-456T
H5	AUT	f1C3	750G-4769G-16304C-146C-195C-263G-309.1C-315.1C-456T
H5	AUT	f3D1	750G-4769G-16304C-16319A-263G-309.1C-309.2C-315.1C-456T-524.1A-524.2C
H5	AUT	hf1B10	750G-4769G-16304C-263G-309.1C-309.2C-315.1C-456T
H5	AUT	hf1C6	750G-4769G-16304C-16311C-199Y-207A-263G-315.1C-456T
H5	AUT	hf1H10	750G-4769G-16304C-263G-309.1C-309.2C-315.1C-456T

H5	AUT	hf1H7	750G-4769G-16241C-16304C-263G-309.1C-315.1C-456T
H5	AUT	hf2A10	750G-4769G-709A-16189C-16193.1C-16193.2C-16304C-200G-263G-309.1C-309.2C-315.1C-456T
H5	AUT	hf2A5	750G-4769G-16129A-16227G-16304C-263G-309.1C-309.2C-315.1C-456T
H5	AUT	hf2B1	750G-4769G-16304C-16311C-146C-207A-263G-315.1C-456T
H5	AUT	hf2E2	750G-4769G-16145A-16304C-263G-309.1C-309.2C-315.1C-456T-478G
H5	AUT	hf2F11	750G-4769G-16304C-16319A-263G-309.1C-309.2C-315.1C-456T-524.1A-524.2C
H5	AUT	hf2H1	750G-4769G-16304C-263G-309.1C-315.1C-456T
H5	AUT	hf3F2	750G-4769G-709A-16189C-16193.1C-16193.2C-16304C-200G-263G-309.1C-309.2C-315.1C-456T
H5	AUT	hf3G12	750G-4769G-16240G-16304C-263G-296T-309.1C-315.1C-456T-524.1A-524.2C
H5	AUT	hf3G3	750G-4769G-16093Y-16304C-263G-315.1C-456T
H5	AUT	hf4B10	750G-4769G-709A-16129A-16304C-263G-309.1C-315.1C-456T
H5	AUT	hf4D2	750G-4769G-709A-16189C-16304C-263G-309.1C-309.2C-315.1C-456T
H5	AUT	hf4D6	750G-4769G-709A-16182C-16183C-16189C-16304C-263G-309.1C-315.1C-456T
H5	AUT	hm1A3	750G-4769G-16304C-16311C-207A-263G-315.1C-456T
H5	AUT	hm1C2	750G-4769G-16304C-263G-309.1C-309.2C-315.1C-456T
H5	AUT	hm1F3	750G-4769G-16048R-16304C-16311C-207A-263G-315.1C-456T
H5	AUT	hm2A9	750G-4769G-16260T-16304C-263G-309.1C-315.1C-456T
H5	AUT	hm2G3	750G-4769G-16124C-16258M-16304C-16311C-263G-315.1C-456T
H5	AUT	hm2H5	750G-4769G-16304C-16319A-189R-263G-309.1C-309.2C-315.1C-456T-524.1A-524.2C
H5	AUT	hm3A1	750G-4769G-16145A-16254G-16304C-16311C-263G-315.1C-456T
H5	AUT	hm3A9	750G-4769G-16093C-16304C-263G-315.1C-456T-496T
H5	AUT	hm3G2	750G-4769G-16048R-16304C-16311C-207A-263G-315.1C-456T
H5	AUT	hm3H12	750G-4769G-709A-16068C-16304C-263G-309.1C-309.2C-315.1C-456T
H5	AUT	hm3H2	750G-4769G-16304C-16319A-189R-263G-309.1C-309.2C-315.1C-456T-524.1A-524.2C
H5	AUT	hm4C1	750G-4769G-16304C-16311C-207A-263G-315.1C-456T
H5	AUT	hm5A1	750G-4769G-16192T-16304C-16311C-16335G-263G-315.1C-456T
H5	AUT	hm5B5	750G-4769G-16304C-16311C-207A-263G-315.1C-456T
H5	AUT	hm5F3	750G-4769G-709A-16189C-16265C-16304C-263G-309.1C-315.1C-456T
H5	AUT	hm5G1	750G-4769G-16304C-16311C-207A-263G-315.1C-456T
H5	AUT	hm6C1	750G-4769G-16304C-16311C-152C-207A-263G-315.1C-456T
H5	AUT	hm6D1	750G-4769G-709A-16189C-16304C-16362Y-263G-309.1C-309.2C-315.1C-456T
H5	AUT	hm6E4	750G-4769G-16070G-152C-263G-315.1C-456T
H5	AUT	hm6E5	750G-4769G-709A-16189C-16304C-263G-309.1C-309.2C-315.1C-456T
H5	AUT	m1H5	750G-4769G-709A-16189C-16304C-263G-309.1C-309.2C-315.1C-456T
H5	AUT	m2B1	750G-4769G-16304C-16311C-199C-207A-263G-315.1C-456T
H5	AUT	m3B1	750G-4769G-16304C-263G-315.1C-456T
H5	MKD	MC1B1	750G-4769G-16291T-16304C-146C-263G-315.1C-456T-573.1C-573.2C-573.3C-573.4C
H5	MKD	MC1E10	750G-4769G-16291T-16304C-146C-263G-315.1C-456T-573.1C-573.2C-573.3C-573.4C
H5	MKD	MC1E8	750G-4769G-16304C-189G-263G-315.1C-456T

H5	MKD	MC1F4	750G-4769G-16070G-152C-263G-309.1C-315.1C-456T
H5	MKD	MC1F6	750G-4769G-16304C-263G-315.1C-373G-456T
H5	MKD	MC1H6	750G-4769G-16304C-152C-263G-315.1C-456T
H5	MKD	MC2C2	750G-4769G-16294T-16304C-263G-315.1C-456T
H5	MKD	MC2D1	750G-4769G-16070G-152C-263G-315.1C-456T-513A
H5	MKD	MC2G3	750G-4769G-16294T-16304C-16519C-263G-315.1C-456T-524.1A-524.2C-524.3A-524.4C
H5	MKD	MC2H6	750G-4769G-16294T-16304C-200G-263G-315.1C-456T-524.1A-524.2C-524.3A-524.4C
H5	ROU	SZ1F4	750G-4769G-16261Y-16304C-16318G-263G-315.1C-456T
H5	ROU	SZ1F5	750G-4769G-16294T-16304C-263G-315.1C-456T
H5	ROU	SZ2C11	750G-4769G-16189C-16294T-16304C-16527T-263G-315.1C-456T
H5	ROU	SZ2E7	750G-4769G-16304C-189G-263G-309.1C-315.1C-456T
H5	ROU	SZ2F6	750G-4769G-709A-16189C-16294T-16304C-16527T-263G-315.1C-456T
H5	ROU	SZ2H2	750G-4769G-16304C-263G-315.1C-315.2C-456T
H5	DEU	UL1A2	750G-4769G-16070G-152C-263G-315.1C-456T-524.1A-524.2C
H5	DEU	UL1B2	750G-4769G-16304C-146C-263G-309.1C-315.1C-456T
H5a	HUN	As2H8	750G-4769G-4336C-16304C-204C-263G-309.1C-315.1C-456T
H5a	AUT	f1F5	750G-4769G-4336C-16260T-16304C-263G-309.1C-315.1C-456T
H5a	AUT	f1G4	750G-4769G-4336C-16304C-263G-309.1C-309.2C-315.1C-456T
H5a	AUT	f2E3	750G-4769G-4336C-16304C-152C-263G-315.1C-456T
H5a	AUT	hf1B11	750G-4769G-4336C-16260T-16304C-263G-309.1C-315.1C-456T
H5a	AUT	hf1B5	750G-4769G-4336C-16304C-146C-263G-309.1C-309.2C-315.1C-456T
H5a	AUT	hf1B6	750G-4769G-4336C-16278T-16304C-263G-309.1C-315.1C-456T-523DEL-524DEL
H5a	AUT	hf1D11	750G-4769G-4336C-16260T-16304C-263G-309.1C-315.1C-456T
H5a	AUT	hf1F5	750G-4769G-4336C-16189C-16193.1C-16193.2C-16304C-263G-309.1C-309.2C-315.1C-456T
H5a	AUT	hf1H3	750G-4769G-4336C-16260T-16304C-263G-309.1C-315.1C-456T
H5a	AUT	hf2C5	750G-4769G-4336C-16260T-16304C-146C-263G-309.1C-315.1C-456T
H5a	AUT	hf2D9	750G-4769G-4336C-16304C-263G-309.1C-315.1C-456T-459T
H5a	AUT	hf3D9	750G-4769G-4336C-16304C-263G-309.1C-309.2C-315.1C-456T
H5a	AUT	hf3F8	750G-4769G-4336C-16304C-263G-309.1C-309.2C-315.1C-456T
H5a	AUT	hf4A7	750G-4769G-4336C-16249C-16304C-263G-309.1C-309.2C-315.1C-456T
H5a	AUT	hf4E6	750G-4769G-4336C-16249C-16304C-152C-263G-309.1C-315.1C-456T
H5a	AUT	hm1B1	750G-4769G-4336C-16304C-263G-309.1C-315.1C-456T-459T
H5a	AUT	hm1C4	750G-4769G-4336C-16304C-152C-263G-315.1C-456T
H5a	AUT	hm1E5	750G-4769G-4336C-16260T-16304C-263G-309.1C-315.1C-456T
H5a	AUT	hm2D4	750G-4769G-4336C-16304C-263G-309.1C-309.2C-315.1C-456T
H5a	AUT	hm2E6	750G-4769G-4336C-16304C-146C-263G-309.1C-309.2C-315.1C-456T
H5a	AUT	hm2G8	750G-4769G-4336C-16304C-152C-263G-315.1C-456T
H5a	AUT	hm3A2	750G-4769G-4336C-16304C-152C-263G-315.1C-456T
H5a	AUT	hm3C10	750G-4769G-4336C-16304C-263G-315.1C-456T-523DEL-524DEL

H5a	AUT	hm4H1	750G-4769G-4336C-16304C-263G-309.1C-315.1C-456T-459T
H5a	AUT	hm4H3	750G-4769G-4336C-16294T-16304C-16320T-152C-263G-315.1C-456T
H5a	AUT	hm5B10	750G-4769G-4336C-16260T-16304C-16311C-263G-309.1C-315.1C-456T
H5a	AUT	hm5C9	750G-4769G-4336C-16304C-146C-263G-309.1C-309.2C-315.1C-456T
H5a	AUT	hm5E7	750G-4769G-4336C-16260T-16304C-263G-309.1C-315.1C-456T
H5a	AUT	hm6F2	750G-4769G-4336C-16093C-16304C-174.1C-263G-315.1C-315.2C-456T
H5a	AUT	m2A4	750G-4769G-4336C-16260T-16304C-263G-309.1C-315.1C-456T
H5a	MKD	MC2E4	750G-4769G-4336C-16304C-263G-309.1C-315.1C-456T-523DEL-524DEL
H5a	ROU	SZ2G5	750G-4769G-4336C-16304C-263G-309.1C-309.2C-315.1C-456T
H5a1	HUN	As2C11	750G-4769G-4336C-15833T-16304C-263G-309.1C-309.2C-315.1C-456T-523DEL-524DEL
H5a1	ROU	CS2H10	750G-4769G-4336C-15833T-16192T-16239T-16304C-263G-309.1C-315.1C-456T-523DEL-524DEL
H5a1	AUT	hf1G6	750G-4769G-4336C-15833T-16048A-16192T-16304C-263G-315.1C-456T-485C-523DEL-524DEL
H5a1	AUT	hf2B3	750G-4769G-4336C-15833T-16278T-16304C-263G-309.1C-315.1C-456T-523DEL-524DEL
H5a1	AUT	hf3B5	750G-4769G-4336C-15833T-16304C-263G-315.1C-456T-523DEL-524DEL
H5a1	AUT	hf3C11	750G-4769G-4336C-15833T-16304C-263G-315.1C-456T-513A
H5a1	AUT	hf4B1	750G-4769G-4336C-15833T-16304C-263G-309.1C-315.1C-456T-523DEL-524DEL
H5a1	AUT	hf4E10	750G-4769G-4336C-15833T-16304C-263G-315.1C-456T-523DEL-524DEL
H5a1	AUT	hm1B3	750G-4769G-4336C-15833T-16304C-151T-263G-309.1C-315.1C-456T-523DEL-524DEL
H5a1	AUT	hm3G5	750G-4769G-4336C-15833T-16274A-16304C-16320T-263G-309.1C-315.1C-456T-523DEL-524DEL
H5a1	AUT	hm4B3	750G-4769G-4336C-15833T-16304C-16519C-227G-263G-315.1C-456T-523DEL-524DEL
H5a1	AUT	hm4C2	750G-4769G-4336C-15833T-16304C-151T-263G-309.1C-315.1C-456T-523DEL-524DEL
H5a1	AUT	hm4E2	750G-4769G-4336C-15833T-16274A-16304C-263G-309.1C-315.1C-456T-523DEL-524DEL
H5a1	AUT	hm4F8	750G-4769G-4336C-15833T-16304C-152C-263G-315.1C-456T-523DEL-524DEL
H5a1	AUT	hm5E9	750G-4769G-4336C-15833T-16304C-151T-263G-309.1C-315.1C-456T-523DEL-524DEL
H5a1	AUT	hm5F11	750G-4769G-4336C-15833T-16192T-16304C-263G-315.1C-456T-523DEL-524DEL
H5a1	AUT	hm5H9	750G-4769G-4336C-15833T-16172C-16304C-16311C-263G-315.1C-444G-456T-523DEL-524DEL
H5a1	AUT	hm6E6	750G-4769G-4336C-15833T-16304C-263G-315.1C-456T-523DEL-524DEL
H5a1	MKD	MC1G12	750G-4769G-4336C-15833T-16304C-263G-309.1C-315.1C-456T-523DEL-524DEL
H5a1	ROU	SZ1D2	750G-4769G-4336C-15833T-16304C-263G-315.1C-456T-523DEL-524DEL
H5a1	ROU	SZ1F6	750G-4769G-4336C-15833T-16304C-263G-315.1C-456T-523DEL-524DEL
H5a1	ROU	SZ1H11	750G-4769G-4336C-15833T-16192Y-16239T-16304C-263G-309.1C-315.1C-456T-523DEL-524DEL
H5a1	DEU	UL1H2	750G-4769G-4336C-15833T-16304C-263G-315.1C-316A-456T-523DEL-524DEL
H5a1	DEU	UL1H8	750G-4769G-4336C-15833T-16209C-16304C-16519C-263G-315.1C-456T-523DEL-524DEL
H6	AUT	hf3E4	750G-4769G-709A-16292T-16362C-16482G-239C-263G-309.1C-315.1C
H6	AUT	hf4H11	750G-4769G-16362C-16482G-239C-263G-315.1C
H6	AUT	hm4F6	750G-4769G-16355Y-16362C-16400T-16482G-239C-263G-315.1C
H6a	MKD	MC2A3	750G-4769G-3915A-16362C-16482G-239C-263G-309.1C-315.1C-522T
H6a1	HUN	As2E10	750G-4769G-3915A-4727G-709A-16362C-16482G-239C-263G-288G-309.1C-309.2C-315.1C
H6a1	ROU	CS1F5	750G-4769G-3915A-4727G-16362C-16482G-239C-263G-309.1C-309.2C-315.1C

H6a1	ARE	DB3G7	750G-4769G-3915A-4727G-16362C-16482G-239C-263G-309.1C-309.2C-315.1C-573.1C
H6a1	AUT	f3D6	750G-4769G-3915A-4727G-16362C-16482G-42.1T-239C-263G-309.1C-315.1C
H6a1	AUT	f3F1	750G-4769G-3915A-4727G-16311C-16362C-16482G-239C-263G-309.1C-309.2C-315.1C
H6a1	AUT	f3H1	750G-4769G-3915A-4727G-16362C-239C-263G-309.1C-309.2C-315.1C
H6a1	AUT	hf1E5	750G-4769G-3915A-4727G-16177G-16213A-16362C-16482G-152C-195C-239C-263G-315.1C
H6a1	AUT	hf1E7	750G-4769G-3915A-4727G-16362C-16482G-239C-263G-315.1C
H6a1	AUT	hf2A11	750G-4769G-3915A-4727G-16362C-16482G-239C-263G-309.1C-315.1C
H6a1	AUT	hf3A11	750G-4769G-3915A-4727G-16362C-16482G-16526A-239C-263G-309.1C-315.1C
H6a1	AUT	hf3B7	750G-4769G-3915A-4727G-16362C-16482G-146C-239C-263G-309.1C-315.1C
H6a1	AUT	hf3C6	750G-4769G-3915A-4727G-16209C-16362C-16482G-16526A-239C-263G-309.1C-309.2C-315.1C
H6a1	AUT	hf3G10	750G-4769G-3915A-4727G-16311C-16362C-16482G-239C-263G-309.1C-315.1C
H6a1	AUT	hf4B8	750G-4769G-3915A-4727G-16362C-16482G-16526A-239C-263G-309.1C-309.2C-315.1C
H6a1	AUT	hf4C5	750G-4769G-3915A-4727G-16209C-16362C-239C-263G-309.1C-315.1C
H6a1	AUT	hm1D3	750G-4769G-3915A-4727G-16362C-16526A-239C-263G-309.1C-315.1C
H6a1	AUT	hm1G1	750G-4769G-3915A-4727G-16362C-16482G-183G-239C-263G-315.1C
H6a1	AUT	hm2A6	750G-4769G-3915A-4727G-16184T-16362C-16482G-239C-263G-315.1C
H6a1	AUT	hm2E10	750G-4769G-3915A-4727G-16184T-16362C-16482G-239C-263G-315.1C
H6a1	AUT	hm2H7	750G-4769G-3915A-4727G-16362C-16526A-239C-263G-309.1C-315.1C
H6a1	AUT	hm3H6	750G-4769G-3915A-4727G-16193T-16219G-16362C-16482G-152C-204C-239C-263G-309.1C-309.2C-315.1C
H6a1	AUT	hm4B7	750G-4769G-3915A-4727G-16249C-16362C-16482G-152C-239C-263G-315.1C
H6a1	AUT	hm4G3	750G-4769G-3915A-4727G-16184T-16362C-16482G-239C-263G-315.1C
H6a1	AUT	hm4H9	750G-4769G-3915A-4727G-16193T-16219G-16362C-16482G-204C-239C-263G-309.1C-309.2C-315.1C-573.1C
H6a1	AUT	hm5B6	750G-4769G-3915A-4727G-16177R-16213A-16362C-16482G-152C-195C-239C-263G-315.1C
H6a1	AUT	hm6D5	750G-4769G-3915A-4727G-16169T-16362C-239C-263G-309.1C-309.2C-315.1C
H6a1	AUT	hm6G6	750G-4769G-3915A-4727G-16362C-16482G-239C-263G-309.1C-309.2C-315.1C
H6a1	AUT	m1F4	750G-4769G-3915A-4727G-16362C-16482G-152C-239C-263G-309.1C-309.2C-315.1C
H6a1	AUT	m1G5	750G-4769G-3915A-4727G-16362C-16482G-183G-239C-263G-315.1C
H6a1	AUT	m2D1	750G-4769G-3915A-4727G-16298C-16362C-16482G-239C-263G-309.1C-315.1C
H6a1	AUT	m3E1	750G-4769G-3915A-4727G-16362C-16482G-16526A-239C-263G-309.1C-315.1C
H6a1	MKD	MC1F5	750G-4769G-3915A-4727G-16362C-16482G-153G-239C-263G-309.1C-315.1C
H6a1	MKD	MC2A4	750G-4769G-3915A-4727G-16298C-16362C-16482G-239C-263G-309.1C-315.1C
H6a1	ROU	SZ1E7	750G-4769G-3915A-4727G-16362C-16482G-239C-263G-315.1C
H6a1	ROU	SZ2B9	750G-4769G-3915A-4727G-16362C-16482G-239C-263G-309.1C-315.1C
H6a1	DEU	UL1H11	750G-4769G-3915A-4727G-16362C-16482G-239C-263G-309.1C-315.1C
H6a1	DEU	UL1F2	750G-4769G-3915A-4727G-16183C-16189C-16193.1C-16362C-16482G-41T-146C-152C-239C-263G-309.1C-309.2C-315.1C
H6b	ARE	DB2D5	750G-4769G-16300G-16325C-16362C-239C-263G-315.1C
H7	HUN	As1B10	750G-4769G-4793G-16519C-263G-309.1C-315.1C
H7	HUN	As1C1	750G-4769G-4793G-16519C-263G-309.1C-315.1C
H7	HUN	As1F12	750G-4769G-4793G-16519C-263G-309.1C-309.2C-315.1C

H7	HUN	As1G9	750G-4769G-4793G-16519C-263G-309.1C-315.1C
H7	HUN	As2A4	750G-4769G-4793G-16519C-263G-309.1C-315.1C
H7	HUN	As2F2	750G-4769G-4793G-16519C-263G-309.1C-315.1C
H7	HUN	As2G11	750G-4769G-4793G-16519C-152C-263G-309.1C-315.1C-573.1C
H7	HUN	As2G5	750G-4769G-4793G-16519C-263G-309.1C-309.2C-315.1C
H7	HUN	As2H4	750G-4769G-4793G-16519C-263G-309.1C-315.1C
H7	ROU	CS1H3	750G-4769G-4793G-16172C-16173T-16519C-263G-309.1C-309.2C-315.1C
H7	ROU	CS2B6	750G-4769G-4793G-16519C-263G-309.1C-315.1C
H7	ROU	CS2C8	750G-4769G-4793G-16172C-16173T-16519C-263G-309.1C-309.2C-315.1C
H7	ROU	CS2D2	750G-4769G-4793G-16261T-16344T-16519C-263G-309.1C-309.2C-315.1C
H7	ROU	CS2E5	750G-4769G-4793G-16172C-16173T-16519C-263G-309.1C-315.1C
H7	ROU	CS2H3	750G-4769G-4793G-16172C-16173T-16519C-263G-309.1C-315.1C
H7	AUT	f1C1	750G-4769G-4793G-16519C-189G-263G-315.1C
H7	AUT	f1E3	750G-4769G-4793G-16093C-16271C-16519C-263G-309.1C-315.1C
H7	AUT	hf1A11	750G-4769G-4793G-16192T-16519C-189G-263G-315.1C
H7	AUT	hf1B9	750G-4769G-4793G-16172C-16519C-152C-263G-309.1C-315.1C
H7	AUT	hf1F4	750G-4769G-4793G-16297C-16365T-16519C-263G-309.1C-315.1C-374G
H7	AUT	hf1F7	750G-4769G-4793G-16297C-16519C-73G-263G-309.1C-315.1C-374G
H7	AUT	hf2C2	750G-4769G-4793G-16519C-263G-309.1C-309.2C-315.1C
H7	AUT	hf3B4	750G-4769G-4793G-16519C-263G-315.1C
H7	AUT	hf3E6	750G-4769G-4793G-16265G-16482G-16519C-151T-263G-309.1C-315.1C
H7	AUT	hf4A2	750G-4769G-4793G-16519C-263G-309.1C-315.1C
H7	AUT	hf4A8	750G-4769G-4793G-16519C-189G-263G-315.1C
H7	AUT	hf4B3	750G-4769G-4793G-16519C-263G-315.1C-524.1A-524.2C-524.3A-524.4C
H7	AUT	hf4B6	750G-4769G-4793G-16519C-189G-263G-315.1C
H7	AUT	hf4D3	750G-4769G-4793G-16297C-16519C-73G-263G-309.1C-315.1C-374G
H7	AUT	hf4G8	750G-4769G-4793G-16519C-189G-263G-315.1C
H7	AUT	hf4H5	750G-4769G-4793G-16519C-263G-315.1C
H7	AUT	hm1G4	750G-4769G-4793G-16172C-16519C-152C-263G-309.1C-315.1C
H7	AUT	hm2C11	750G-4769G-4793G-16357C-16519C-152C-263G-309.1C-315.1C-573.1C
H7	AUT	hm3H11	750G-4769G-4793G-16261T-16519C-263G-309.1C-315.1C
H7	AUT	hm4B1	750G-4769G-4793G-16519C-263G-309.1C-315.1C
H7	AUT	hm4C4	750G-4769G-4793G-16093C-16169T-16265G-16519C-263G-309.1C-315.1C
H7	AUT	hm5G9	750G-4769G-4793G-16519C-263G-315.1C
H7	AUT	hm6F4	750G-4769G-4793G-16519C-189G-263G-315.1C
H7	MKD	MC1A9	750G-4769G-4793G-16519C-263G-309.1C-315.1C
H7	MKD	MC1C4	750G-4769G-4793G-16519C-263G-309.1C-315.1C
H7	MKD	MC1E1	750G-4769G-4793G-16298C-16519C-185A-235G-263G-309.1C-315.1C
H7	MKD	MC3F1	750G-4769G-4793G-16519C-263G-309.1C-309.2C-315.1C

H7	ROU	SZ1D11	750G-4769G-4793G-16261T-16304C-16519C-93G-263G-315.1C-523DEL-524DEL
H7	ROU	SZ1G12	750G-4769G-4793G-16519C-263G-309.1C-315.1C
H7	ROU	SZ1H4	750G-4769G-4793G-16519C-263G-309.1C-315.1C
H7	ROU	SZ2A11	750G-4769G-4793G-709A-16172C-16173T-16519C-263G-309.1C-309.2C-315.1C
H7	ROU	SZ2B2	750G-4769G-4793G-709A-16172C-16173T-16519C-263G-309.1C-309.2C-315.1C
H7	ROU	SZ2B5	750G-4769G-4793G-16172C-16173T-16272G-16519C-263G-309.1C-309.2C-315.1C
H7	ROU	SZ2D10	750G-4769G-4793G-709A-16172C-16173T-16519C-263G-309.1C-309.2C-315.1C
H7	ROU	SZ2F1	750G-4769G-4793G-709A-16172C-16173T-16519C-263G-309.1C-309.2C-315.1C
H7	ROU	SZ2F8	750G-4769G-4793G-709A-16172C-16173T-16519C-263G-309.1C-309.2C-315.1C
H7	ROU	SZ2G2	750G-4769G-4793G-709A-16172C-16173T-16519C-263G-309.1C-309.2C-315.1C
H7	ROU	SZ2G8	750G-4769G-4793G-16172C-16173T-16519C-263G-309.1C-309.2C-315.1C
H8	AUT	hf2G8	750G-4769G-13101C-709A-16111T-16167T-16288C-16362C-16519C-146C-195C-263G-309.1C-315.1C
H8	DEU	UL1F6	750G-4769G-13101C-709A-16288C-16311C-16362C-146C-195C-263G-309.1C-309.2C-315.1C-486A
H9	MKD	MC1G1	750G-4769G-4310G-16168T-16519C-152C-263G-315.1C
HV	HUN	As1C7	750G-4769G-2706G-7028T-263G-315.1C
HV	ARE	DB1A11	750G-4769G-2706G-7028T-16129A-16176T-16178C-16311C-263G-309.1C-309.2C-315.1C-480C
HV	ARE	DB1A2	750G-4769G-2706G-7028T-16286Y-263G-315.1C-460A
HV	ARE	DB1B6	750G-4769G-2706G-7028T-16223T-16240G-146C-235G-263G-315.1C-523DEL-524DEL
HV	ARE	DB1C1	750G-4769G-2706G-7028T-16223T-235G-263G-315.1C-523DEL-524DEL
HV	ARE	DB1D4	750G-4769G-2706G-7028T-16223T-235G-263G-315.1C-523DEL-524DEL
HV	ARE	DB2A5	750G-4769G-2706G-7028T-16223T-235G-263G-315.1C-523DEL-524DEL
HV	ARE	DB2B11	750G-4769G-2706G-7028T-16183C-16189C-16192T-263G-309.1C-315.1C
HV	ARE	DB2G10	750G-4769G-2706G-7028T-16183M-16189C-16192T-263G-309.1C-315.1C
HV	ARE	DB2H12	750G-4769G-2706G-7028T-16223T-235G-263G-315.1C-523DEL-524DEL
HV	ARE	DB3A8	750G-4769G-2706G-7028T-16189C-16192T-263G-309.1C-315.1C
HV	ARE	DB3C10	750G-4769G-2706G-7028T-16519C-150T-263G-309.1C-309.2C-315.1C-524.1A-524.2C
HV	ARE	DB3C2	750G-4769G-2706G-7028T-16286T-263G-315.1C-460A
HV	ARE	DB3G5	750G-4769G-2706G-7028T-93G-146C-234G-263G-315.1C
HV	AUT	f3B5	750G-4769G-2706G-7028T-16248T-146C-263G-309.1C-309.2C-315.1C
HV	AUT	m3C2	750G-4769G-2706G-7028T-16311C-263G-309.1C-315.1C
HV	MKD	MC2A8	750G-4769G-2706G-7028T-16311C-152Y-263G-309.1C-309.2C-315.1C
HV	MKD	MC2D12	750G-4769G-2706G-7028T-16172C-16311C-143A-199C-263G-309.1C-315.1C
HV	ROU	SZ1B2	750G-4769G-2706G-7028T-16278T-16311C-16519C-263G-315.1C
HV	ROU	SZ1F8	750G-4769G-2706G-7028T-16311C-263G-315.1C
HV	ROU	SZ2D9	750G-4769G-2706G-7028T-16183C-16189C-16193.1C-16311C-197G-263G-315.1C-523DEL-524DEL
HV0	HUN	As1B4	750G-4769G-2706G-7028T-16294T-16298C-72C-195C-263G-309.1C-315.1C
HV0	ROU	CS1F1	750G-4769G-2706G-7028T-16298C-16311C-72C-263G-309.1C-309.2C-315.1C
HV0	ROU	CS2E11	750G-4769G-2706G-7028T-16298C-72C-195C-215R-263G-315.1C
HV0	ROU	CS2H2	750G-4769G-2706G-7028T-16298C-72C-195C-263G-315.1C

HV0	AUT	f3G1	750G-4769G-2706G-7028T-16298C-16311Y-64T-72C-195C-263G-309.1C-315.1C
HV0	AUT	m2G4	750G-4769G-2706G-7028T-16183C-16189C-16298C-72C-195C-263G-309.1C-309.2C-315.1C
HV0	AUT	m2H2	750G-4769G-2706G-7028T-16298C-72C-152C-195C-263G-315.1C
HV0	MKD	MC2C12	750G-4769G-2706G-7028T-16298C-16311C-72C-263G-309.1C-315.1C
HV0	MKD	MC2D5	750G-4769G-2706G-7028T-16291T-16298C-16353T-72C-195C-263G-315.1C
HV0	MKD	MC2F9	750G-4769G-2706G-7028T-16298C-16311C-72C-263G-309.1C-315.1C
HV0	MKD	MC2H2	750G-4769G-2706G-7028T-16298C-16311C-72C-263G-309.1C-315.1C
HV0	ROU	SZ1E4	750G-4769G-2706G-7028T-16298C-16311C-72C-263G-309.1C-309.2C-315.1C
HV0	ROU	SZ1H2	750G-4769G-2706G-7028T-16298C-72C-195C-263G-315.1C
HV0	DEU	UL1C7	750G-4769G-2706G-7028T-16298C-72C-195C-198T-263G-309.1C-315.1C
HV0a	ROU	CS1G2	750G-4769G-2706G-7028T-15904T-16298C-72C-263G-295T-309.1C-315.1C
HV0a	MKD	MC2C1	750G-4769G-2706G-7028T-15904T-16298C-16390A-72C-263G-295T-309.1C-309.2C-315.1C
HV0a	MKD	MC2C6	750G-4769G-2706G-7028T-15904T-16093Y-16126C-16298C-72C-263G-309.1C-315.1C-523DEL-524DEL
HV0a	ROU	SZ1G9	750G-4769G-2706G-7028T-15904T-16298C-72C-263G-295T-309.1C-309.2C-315.1C
HV1	HUN	As1H3	750G-4769G-2706G-7028T-15218G-16067T-16183C-16189C-16193.1C-16519C-152C-263G-309.1C-309.2C-315.1C
HV1	HUN	As2G7	750G-4769G-2706G-7028T-15218G-16067T-16182C-16183C-16189C-16519C-152C-263G-309.1C-309.2C-309.3C-315.1C
HV1	HUN	As2H7	750G-4769G-2706G-7028T-15218G-16067T-16182C-16183C-16189C-16519C-152C-263G-309.1C-309.2C-309.3C-315.1C
HV1	ARE	DB2G7	750G-4769G-2706G-7028T-16067T-16362C-249DEL-263G-315.1C
HV1	ROU	SZ2H1	750G-4769G-2706G-7028T-15218G-16067T-16129A-16242T-152C-263G-309.1C-309.2C-315.1C-560T
R0a	ARE	DB1G3	750G-4769G-14766T-2706G-7028T-3847C-16126C-16311C-16355T-16362C-64T-73G-146C-263G-309.1C-315.1C
R0a	ARE	DB3F5	750G-4769G-14766T-2706G-7028T-3847C-16126C-16183C-16189C-16232A-16362C-64T-263G-309.1C-309.2C-315.1C
R0a1a	ARE	DB1C11	750G-4769G-14766T-2706G-7028T-3847C-16126C-16355T-16362C-58C-64T-146C-152C-263G-315.1C
R0a1a	ARE	DB1D11	750G-4769G-14766T-2706G-7028T-3847C-16126C-16355T-16362C-58C-64T-146C-152C-263G-315.1C
R0a1a	ARE	DB2B12	750G-4769G-14766T-2706G-7028T-3847C-16126C-16355T-16362C-58C-64T-73G-146C-152C-195C-263G-309.1C-315.1C
R0a1a	ARE	DB2F12	750G-4769G-14766T-2706G-7028T-3847C-16126C-16355T-16362C-58C-64T-146C-263G-309.1C-315.1C-374G
R0a1a	ARE	DB3E9	750G-4769G-14766T-2706G-7028T-3847C-16126C-16355T-16362C-16519C-58C-64T-146C-152C-263G-315.1C
R0a1a	ROU	SZ1E12	750G-4769G-14766T-2706G-7028T-3847C-16126C-16355T-16362C-58C-64T-146C-152C-263G-309.1C-309.2C-315.1C
R0a1a	ROU	SZ1H3	750G-4769G-14766T-2706G-7028T-3847C-16126C-16355T-16362C-58C-64T-146C-152C-263G-309.1C-309.2C-315.1C
R0a2	ARE	DB1C12	750G-4769G-14766T-2706G-7028T-3847C-16037G-16092C-16126C-16362C-58C-60.1T-64T-263G-315.1C
R0a2	ARE	DB1F1	750G-4769G-14766T-2706G-7028T-3847C-16126C-16172C-16184A-16362C-58C-60.1T-64T-263G-309.1C-315.1C
R0a2	ARE	DB2B5	750G-4769G-14766T-2706G-7028T-3847C-16126C-16172C-16184A-16362C-58C-60.1T-64T-263G-309.1C-309.2C-315.1C-320T
R0a2	ARE	DB2C5	750G-4769G-14766T-2706G-7028T-3847C-16126C-16362C-16519C-58C-60.1T-64T-263G-309.1C-315.1C
R0a2	ARE	DB2E8	750G-4769G-14766T-2706G-7028T-3847C-16126C-16264T-16362C-58C-60.1T-64T-263G-309.1C-309.2C-315.1C-568T-573.1C-573.2C-573.3C-573.4C-573.5C-573.6C-573.7C
R0a2	ARE	DB3E11	750G-4769G-14766T-2706G-7028T-3847C-16126C-16264T-16362C-58C-60.1T-64T-263G-309.1C-315.1C-568T-573.1C-573.2C-573.3C-573.4C-573.5C
R0a2	ROU	CS1B3	750G-4769G-14766T-2706G-7028T-3847C-16114T-16126C-16258C-16319A-16362C-16519C-60.1T-64T-152C-263G-309.1C-315.1C
R0a2	ROU	CS1D6	750G-4769G-14766T-2706G-7028T-3847C-16114Y-16126C-16258C-16319A-16362C-16519C-58C-60.1T-64T-152C-263G-309.1C-315.1C
R0a2	ROU	CS2D4	750G-4769G-14766T-2706G-7028T-3847C-16114T-16126C-16258C-16319A-16362C-16519C-58C-60.1T-64T-152C-263G-309.1C-315.1C-

			502T
R0a2	ROU	SZ1A5	750G-4769G-14766T-2706G-7028T-3847C-16114T-16126C-16258C-16319A-16362C-16519C-58C-60.1T-64T-152C-263G-309.1C-315.1C
R0a2	ROU	SZ1F3	750G-4769G-14766T-2706G-7028T-3847C-16114T-16126C-16258C-16319A-16362C-16519C-58C-60.1T-64T-152C-263G-309.1C-315.1C
V	HUN	As1B1	750G-4769G-2706G-7028T-15904T-4580A-16298C-72C-263G-309.1C-315.1C
V	HUN	As1C9	750G-4769G-2706G-7028T-15904T-4580A-16298C-16519C-72C-263G-315.1C
V	HUN	As1E9	750G-4769G-2706G-7028T-15904T-4580A-16153A-16298C-72C-89C-93G-195C-263G-309.1C-315.1C
V	HUN	As1G11	750G-4769G-2706G-7028T-15904T-4580A-16126C-16298C-72C-263G-309.1C-315.1C
V	HUN	As1G8	750G-4769G-2706G-7028T-15904T-4580A-16153A-16298C-72C-89C-93G-195C-263G-309.1C-315.1C
V	HUN	As2A2	750G-4769G-2706G-7028T-15904T-4580A-16298C-72C-263G-309.1C-309.2C-315.1C
V	HUN	As2A9	750G-4769G-2706G-7028T-15904T-4580A-16298C-16301T-16311C-16362C-72C-263G-309.1C-315.1C
V	HUN	As2C6	750G-4769G-2706G-7028T-15904T-4580A-16153A-16298C-16327Y-72C-89C-93G-195C-263G-309.1C-315.1C
V	HUN	As2D2	750G-4769G-2706G-7028T-15904T-4580A-16298C-72C-263G-309.1C-309.2C-315.1C
V	HUN	As2G3	750G-4769G-2706G-7028T-15904T-4580A-16298C-72C-263G-309.1C-309.2C-315.1C-508G
V	HUN	As2G9	750G-4769G-2706G-7028T-15904T-4580A-16153A-16298C-72C-89C-93G-195C-263G-309.1C-309.2C-315.1C
V	AUT	f1G1	750G-4769G-2706G-7028T-15904T-4580A-16153A-16298C-72C-93G-195C-263G-315.1C
V	AUT	m3C4	750G-4769G-2706G-7028T-15904T-4580A-16216G-16261T-16298C-16519C-72C-263G-309.1C-315.1C
V	AUT	m3D1	750G-4769G-2706G-7028T-15904T-4580A-16145A-16298C-16519C-72C-263G-309.1C-315.1C
V	AUT	m3G4	750G-4769G-2706G-7028T-15904T-4580A-16162G-16298C-263G-309.1C-315.1C
V	MKD	MC1B8	750G-4769G-2706G-7028T-15904T-4580A-16298C-16519C-72C-263G-309.1C-315.1C
V	MKD	MC1H7	750G-4769G-2706G-7028T-15904T-4580A-16298C-16519C-263G-309.1C-315.1C
V	MKD	MC2E7	750G-4769G-2706G-7028T-15904T-4580A-16274A-16298C-16311C-16519C-72C-263G-309.1C-309.2C-315.1C
V	MKD	MC3H1	750G-4769G-2706G-7028T-15904T-4580A-16298C-72C-263G-309.1C-315.1C
V	ROU	SZ1B6	750G-4769G-2706G-7028T-15904T-4580A-16180G-16298C-72C-263G-309.1C-315.1C
V	ROU	SZ1C5	750G-4769G-2706G-7028T-15904T-4580A-16298C-152C-263G-309.1C-315.1C
V	ROU	SZ1G3	750G-4769G-2706G-7028T-15904T-4580A-16298C-72C-188G-204C-263G-315.1C
V	ROU	SZ2C5	750G-4769G-2706G-7028T-15904T-4580A-16298C-72C-204C-263G-309.1C-315.1C
V	ROU	SZ2H10	750G-4769G-2706G-7028T-15904T-4580A-16298C-152C-263G-309.1C-315.1C
V	ROU	SZ2H4	750G-4769G-2706G-7028T-15904T-4580A-16298C-72C-188G-204C-263G-309.1C-315.1C
V	DEU	UL1A9	750G-4769G-2706G-7028T-15904T-4580A-16153A-16298C-72C-93G-263G-309.1C-309.2C-315.1C
V	DEU	UL1H4	750G-4769G-2706G-7028T-15904T-4580A-16216G-16298C-16519C-263G-309.1C-315.1C
