# Supplementary figures and images for: Timing and deciphering mitochondrial DNA macro-haplogroup R0 variability in Central Europe and Middle East
Source: BMC Evol Biol. 2008 Jul 4;8:191. doi: 10.1186/1471-2148-8-191 (PMC2491632; doi:10.1186/1471-2148-8-191)

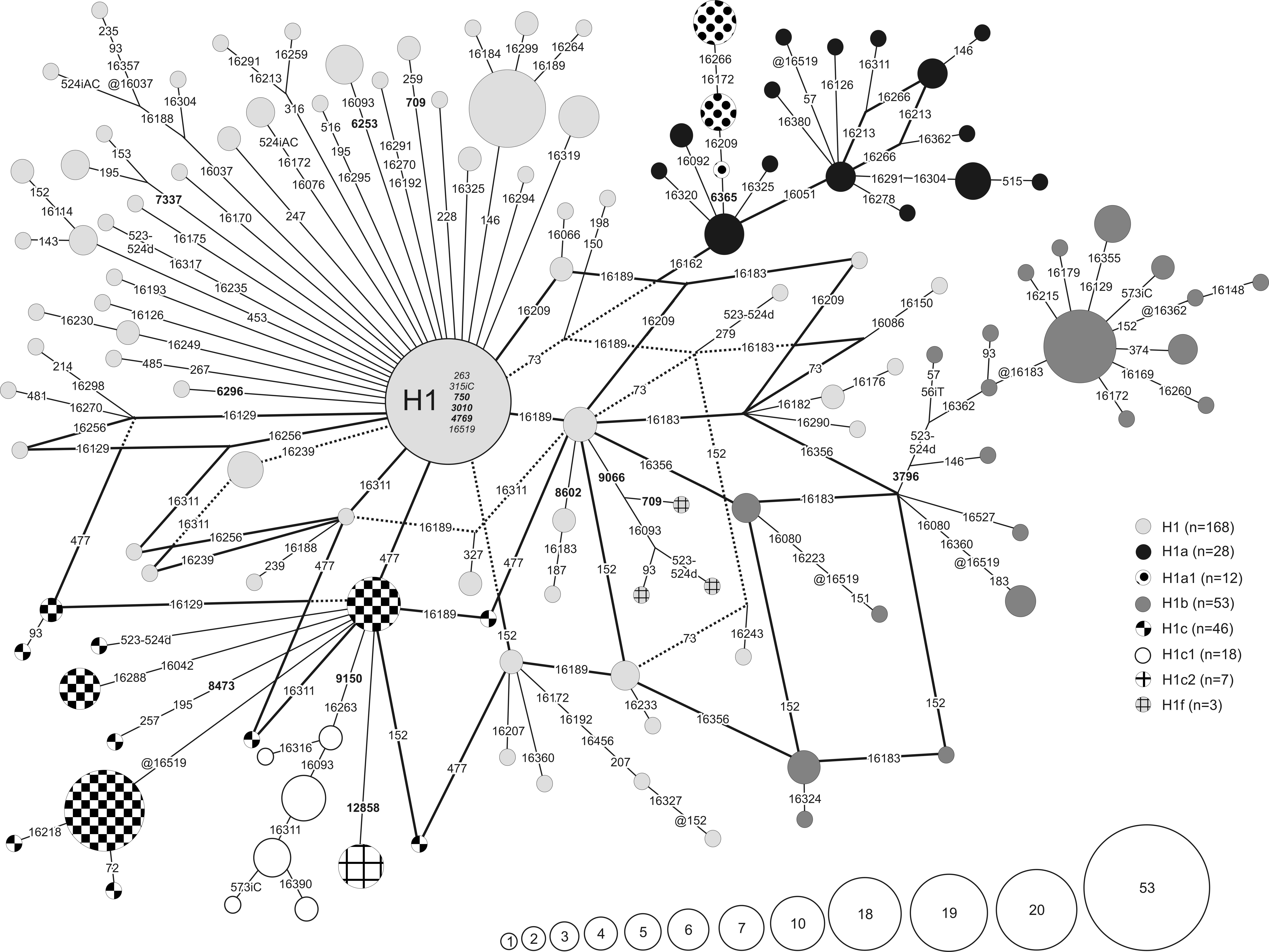

Supplement: Additional file 2 — Median joining network of hg H1. Node sizes are proportional to haplotype frequencies. Variable positions are indicated along links that connect haplotypes. Nucleotide changes are specified only in the case of transversions. Polymorphisms in bold correspond to coding region sites. Italic polymorphisms are those of the central haplotype and are found in all haplotypes of the respective network if not indicated otherwise. [file 1471-2148-8-191-S2.jpeg]

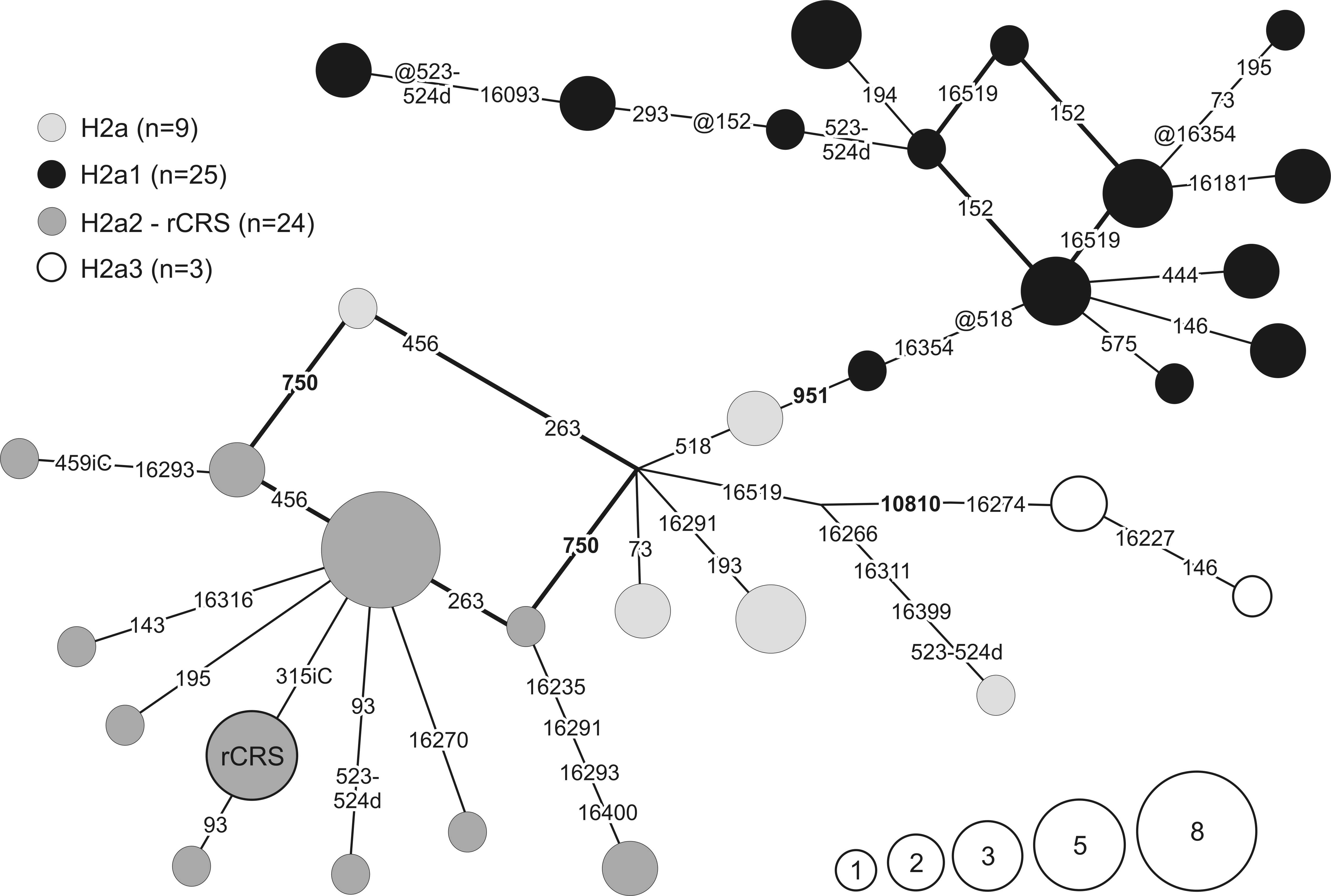

Supplement: Additional file 3 — Median joining network of hg H2a. please see Additional file 2. [file 1471-2148-8-191-S3.jpeg]

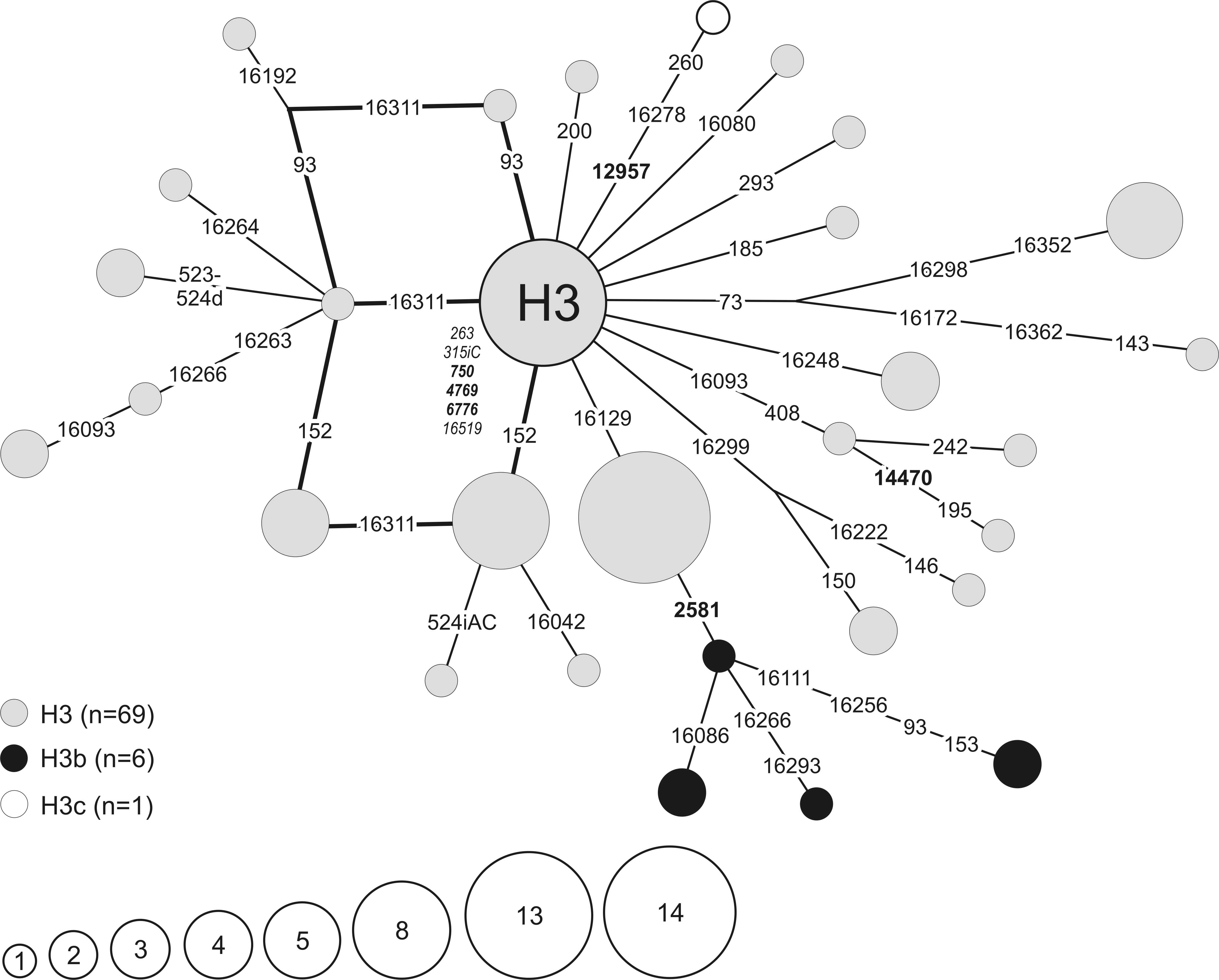

Supplement: Additional file 4 — Median joining network of hg H3. please see Additional file 2. [file 1471-2148-8-191-S4.jpeg]

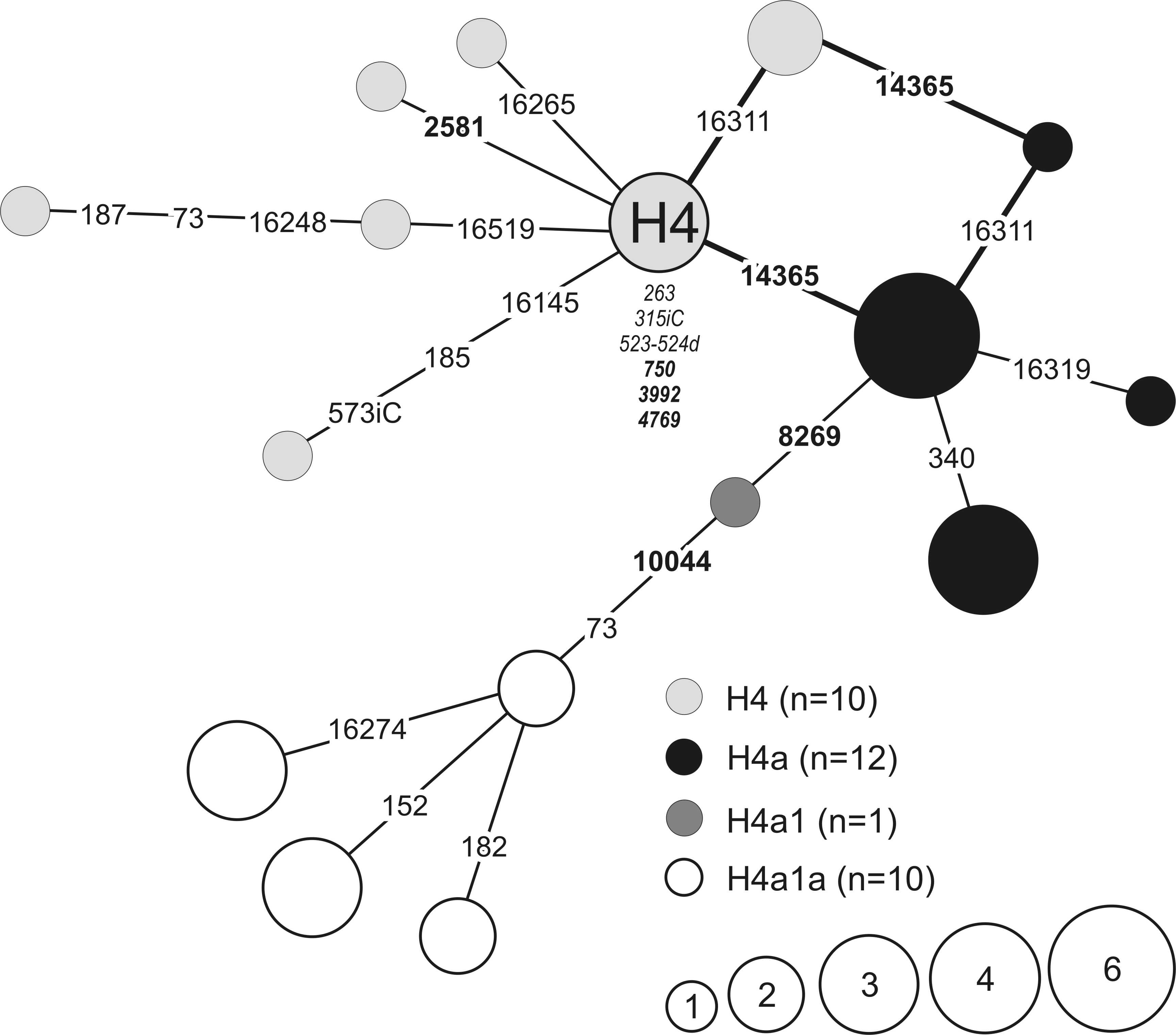

Supplement: Additional file 5 — Median joining network of hg H4. please see Additional file 2. [file 1471-2148-8-191-S5.jpeg]

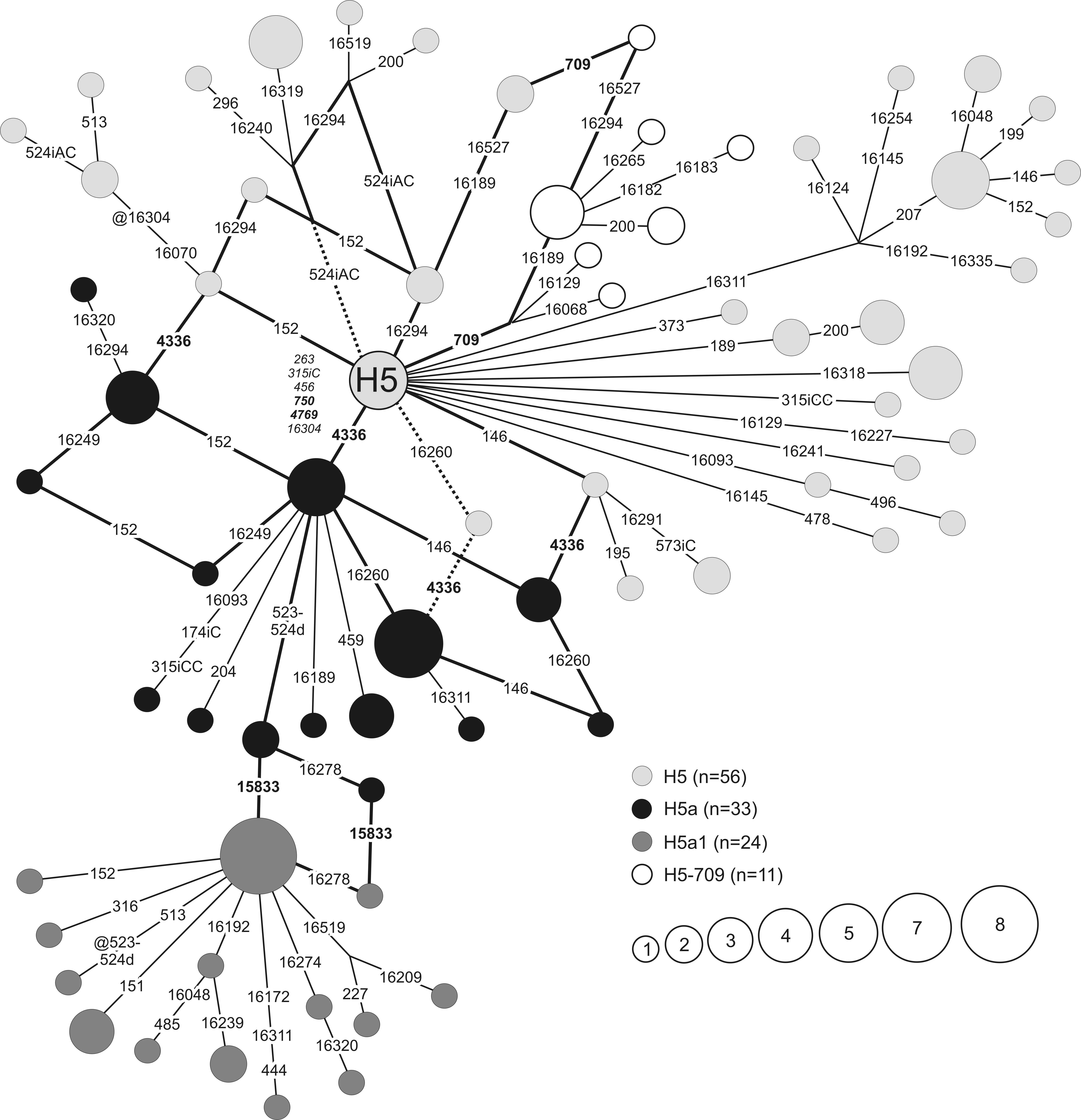

Supplement: Additional file 6 — Median joining network of hg H5. please see Additional file 2. [file 1471-2148-8-191-S6.jpeg]

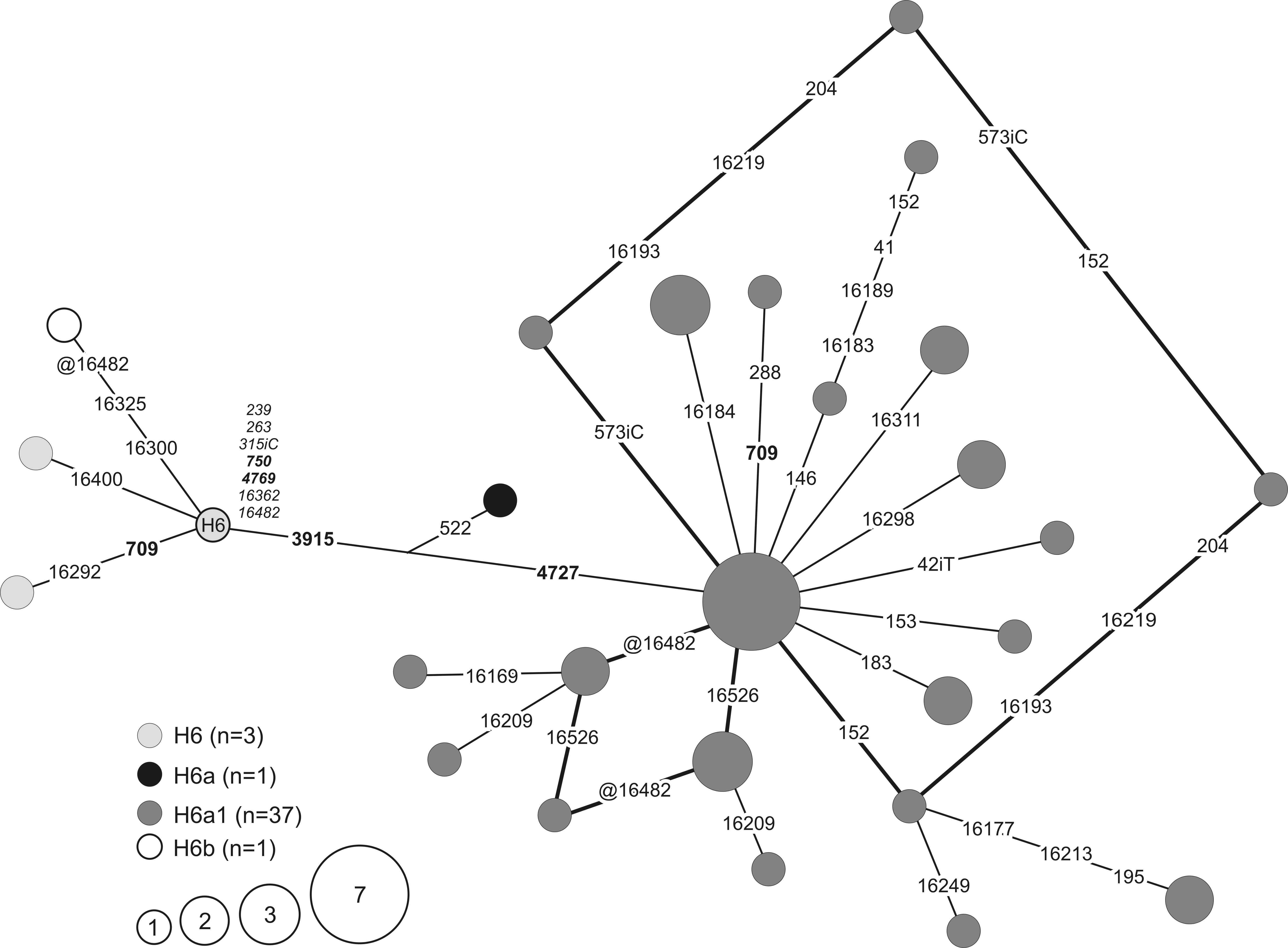

Supplement: Additional file 7 — Median joining network of hg H6. please see Additional file 2. [file 1471-2148-8-191-S7.jpeg]

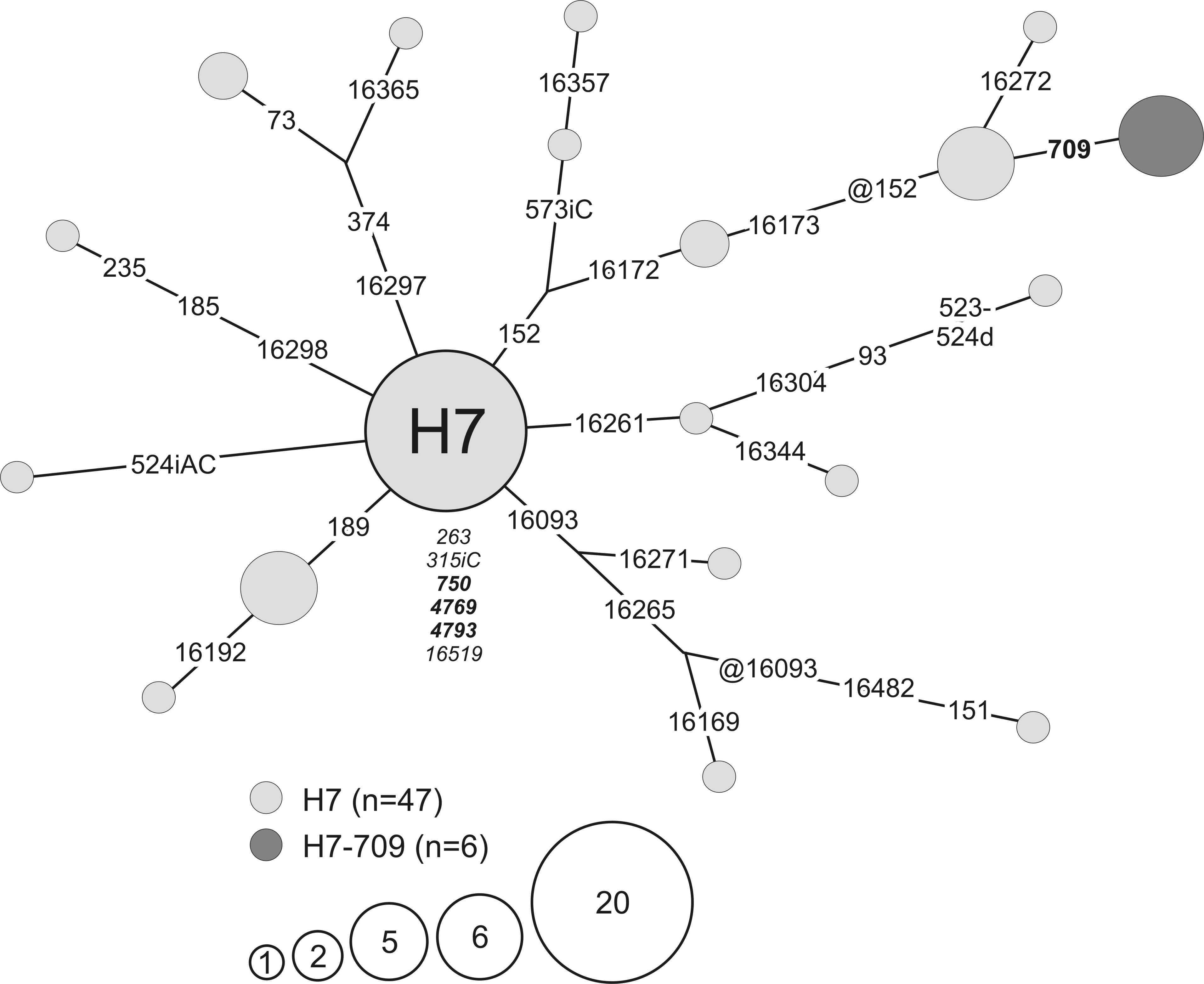

Supplement: Additional file 8 — Median joining network of hg H7. please see Additional file 2. [file 1471-2148-8-191-S8.jpeg]

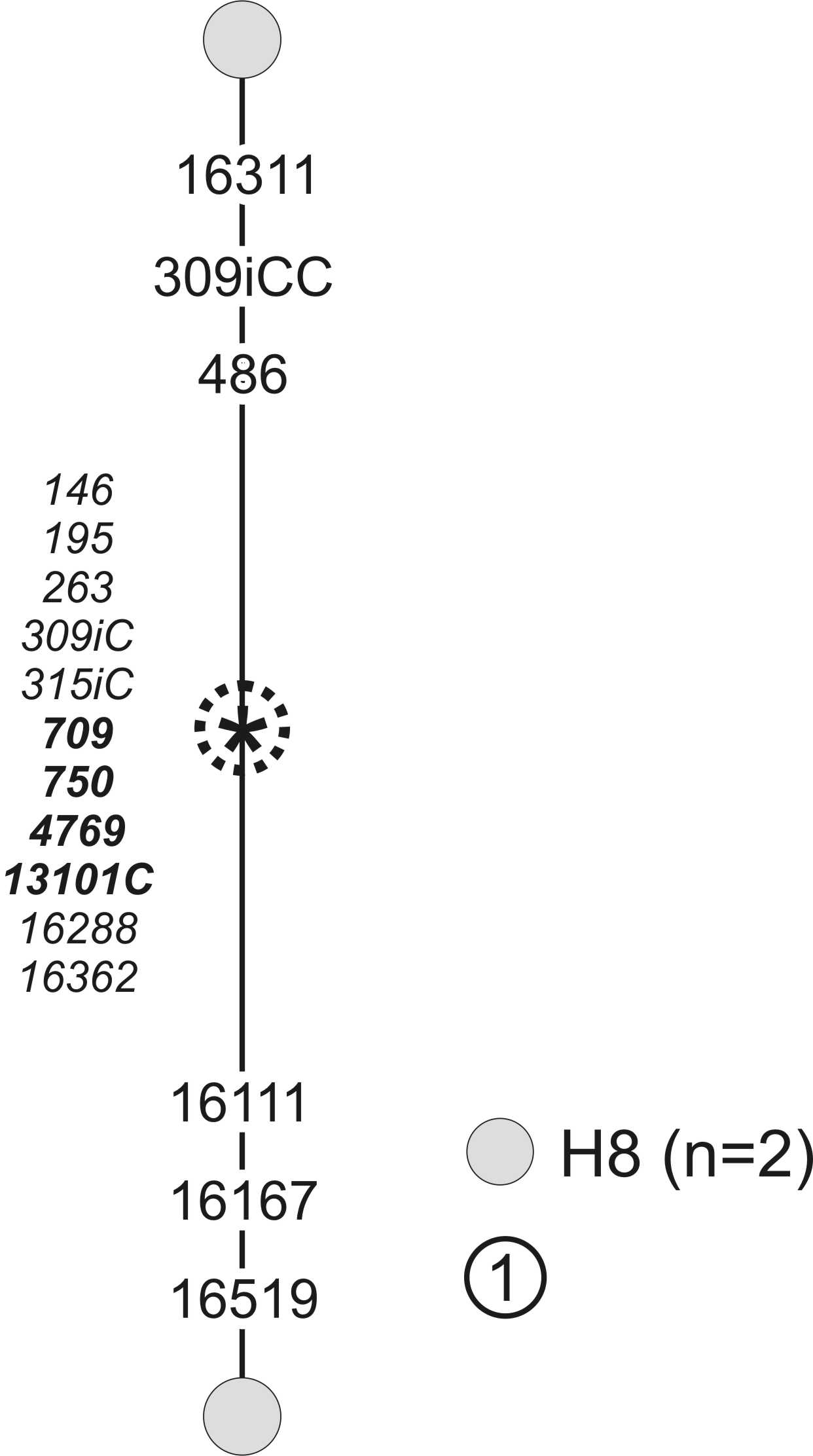

Supplement: Additional file 9 — Median joining network of hg H8. please see Additional file 2. [file 1471-2148-8-191-S9.jpeg]

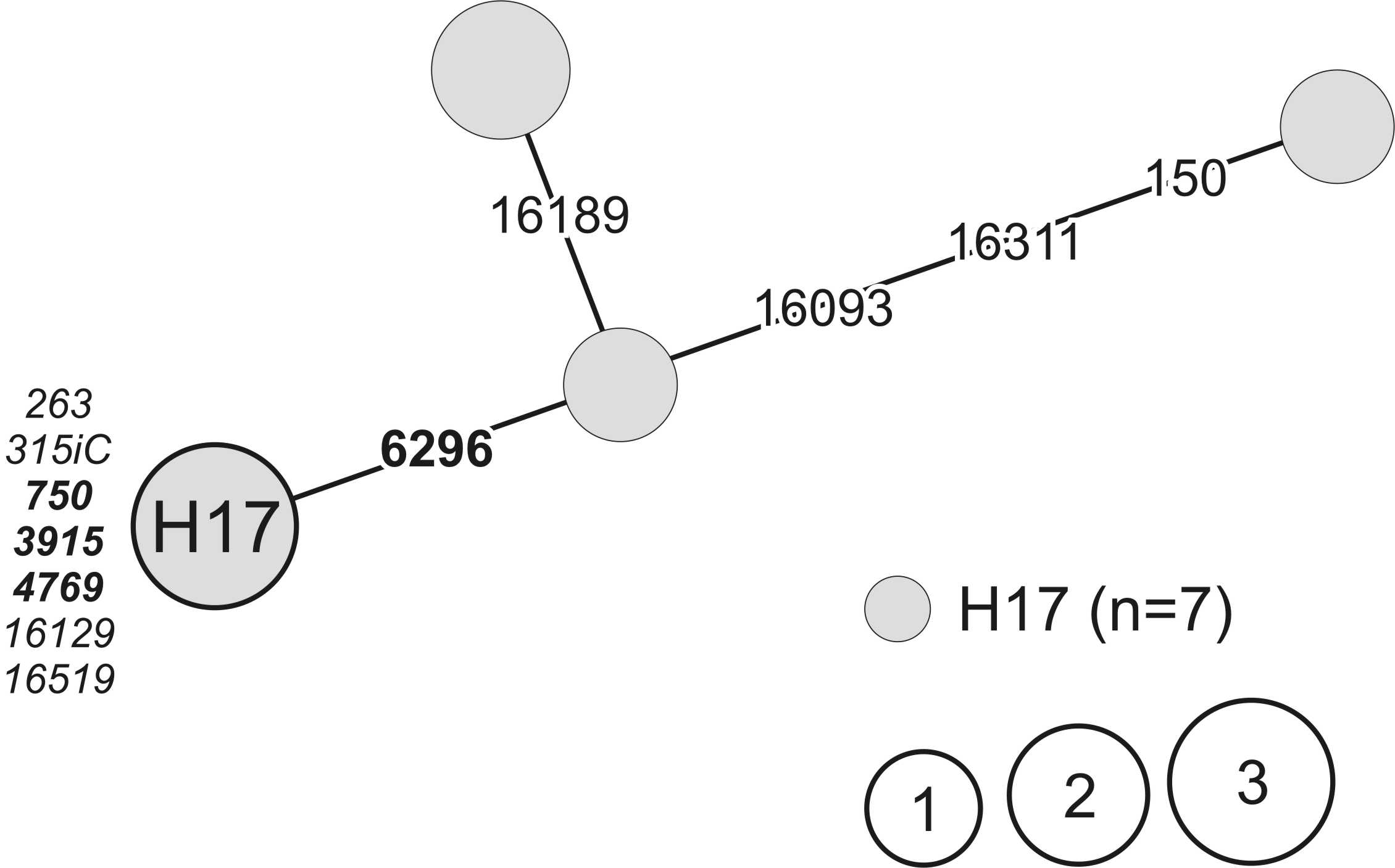

Supplement: Additional file 10 — Median joining network of hg H17. please see Additional file 2. [file 1471-2148-8-191-S10.jpeg]

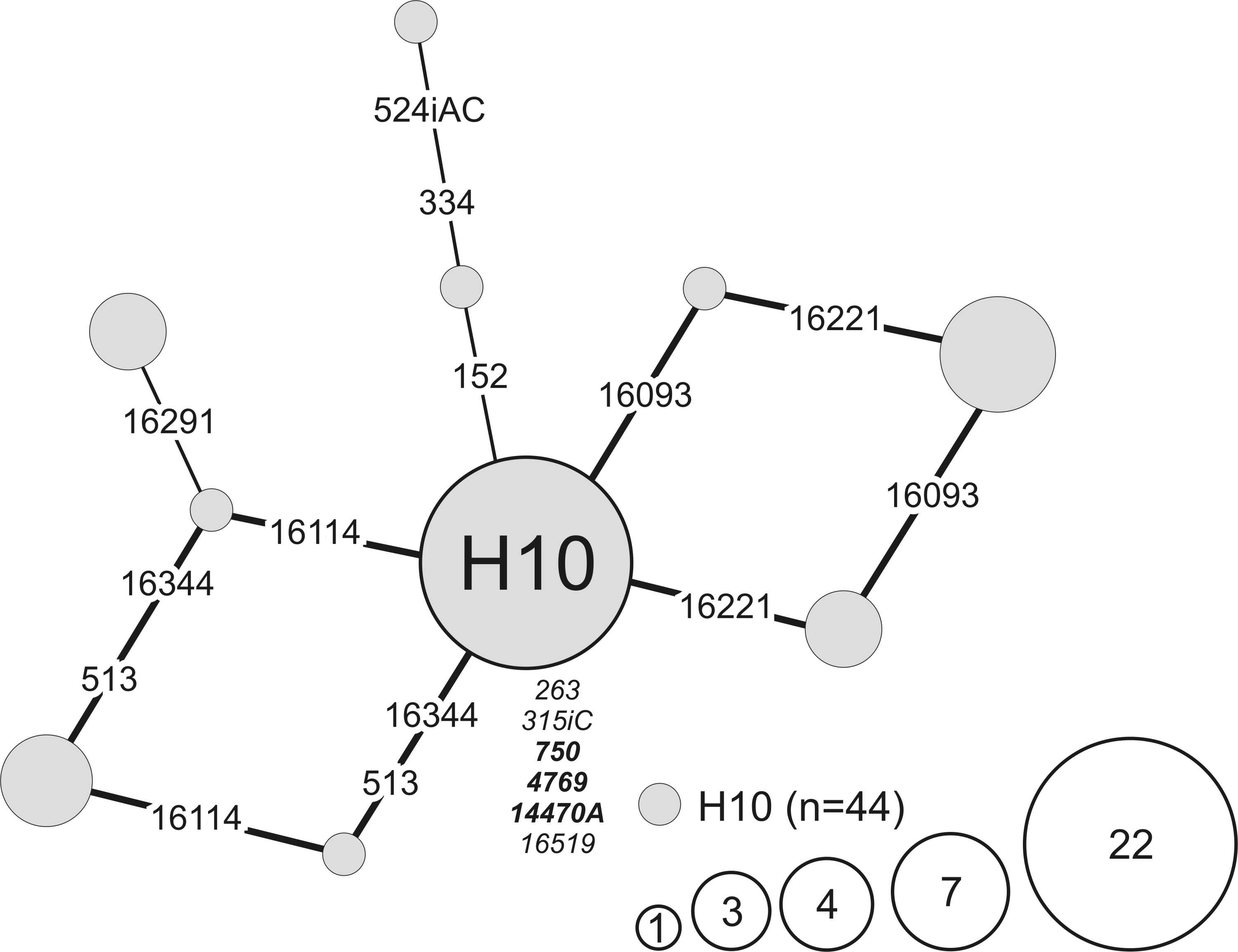

Supplement: Additional file 11 — Median joining network of hg H10. please see Additional file 2. [file 1471-2148-8-191-S11.jpeg]

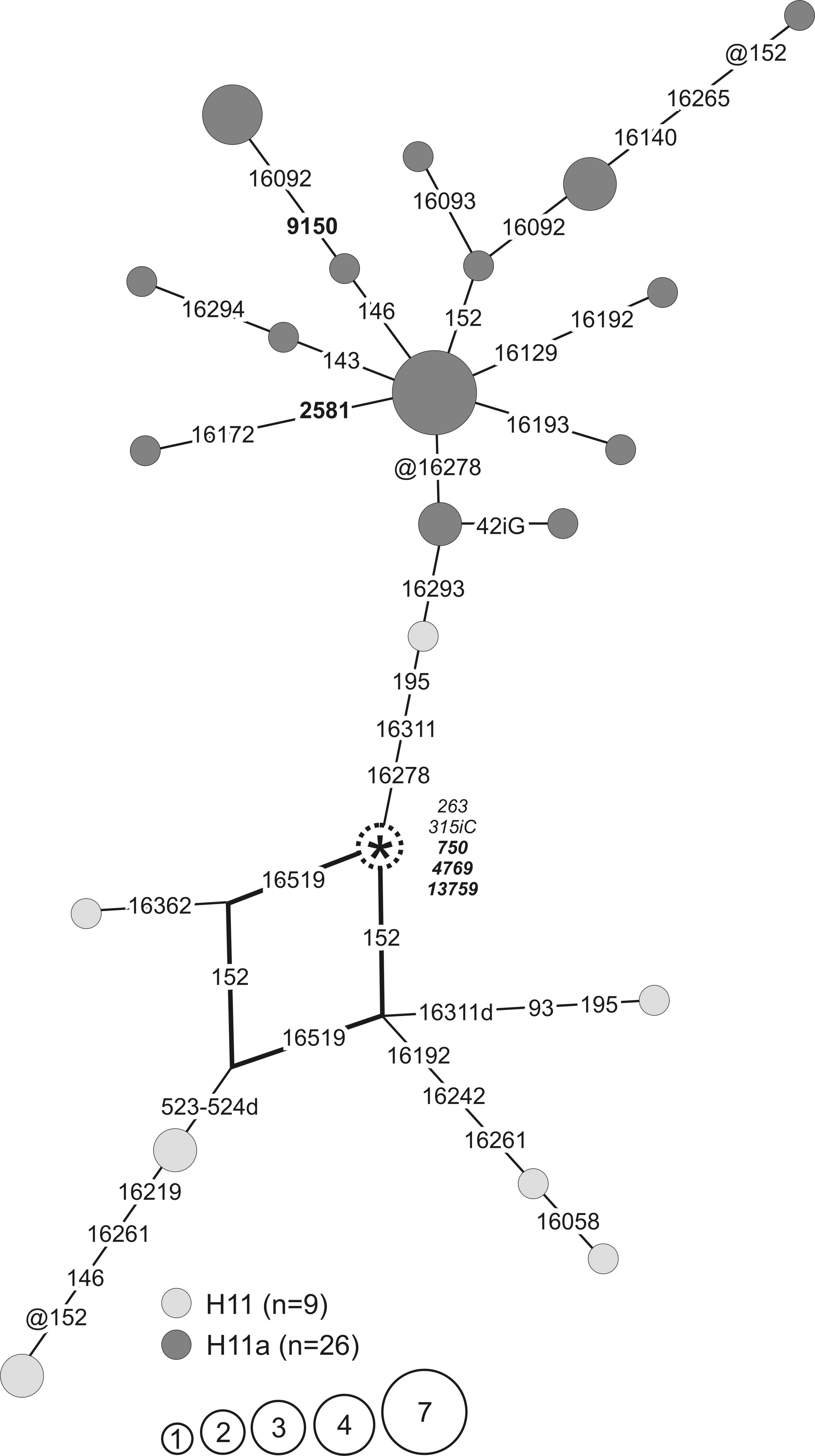

Supplement: Additional file 12 — Median joining network of hg H11. please see Additional file 2. [file 1471-2148-8-191-S12.jpeg]

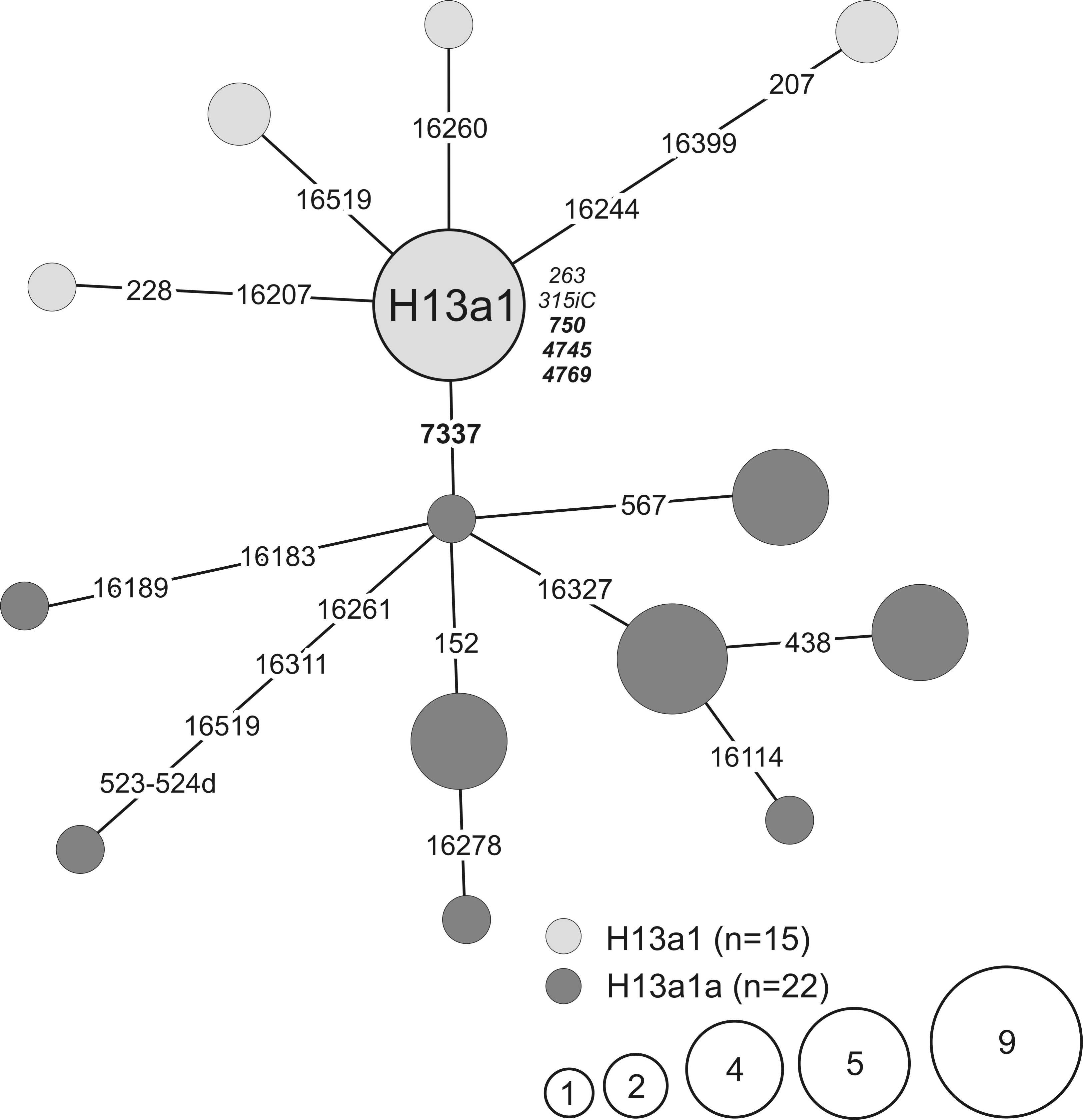

Supplement: Additional file 13 — Median joining network of hg H13a1. please see Additional file 2. [file 1471-2148-8-191-S13.jpeg]

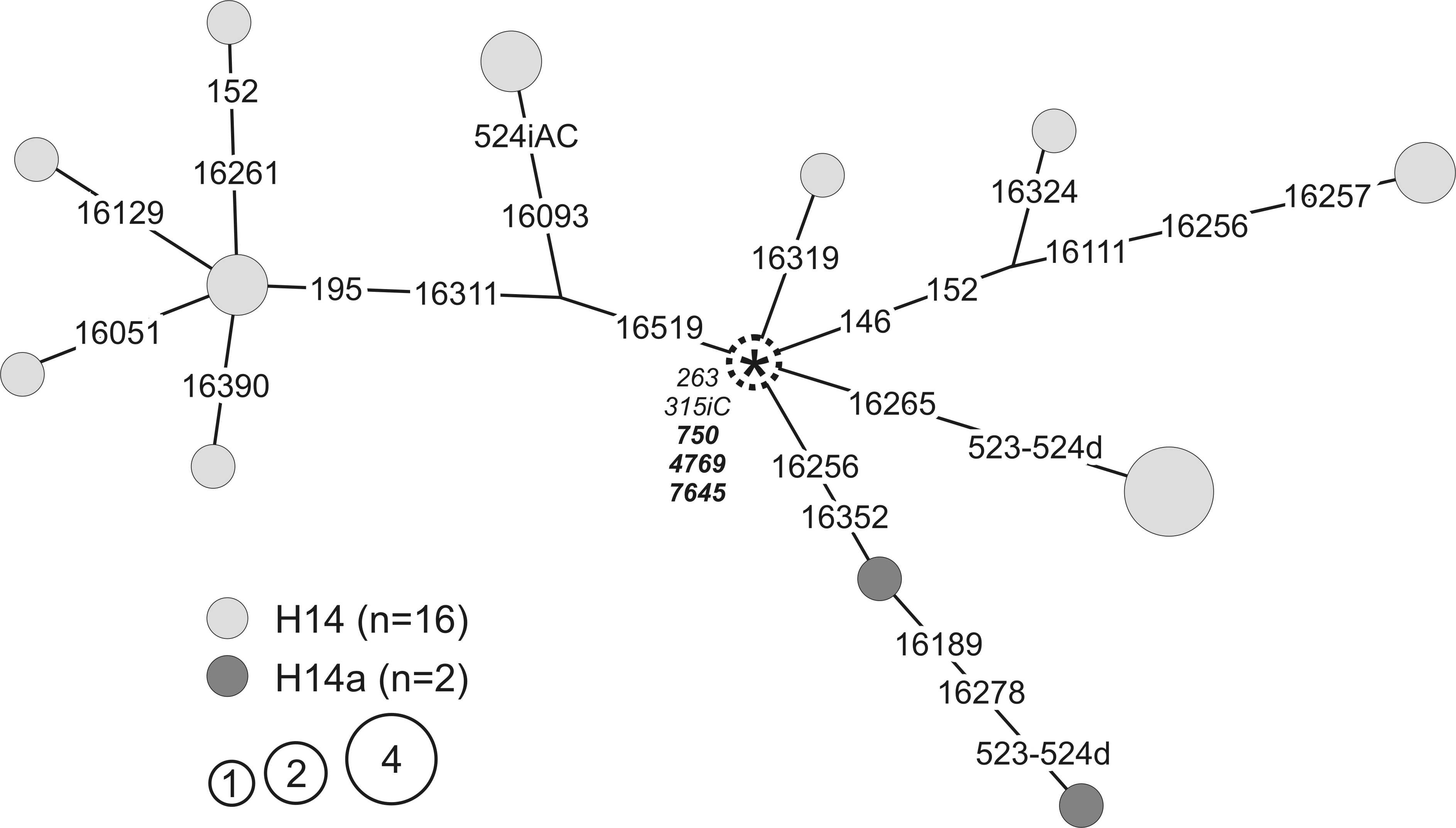

Supplement: Additional file 14 — Median joining network of hg H14. please see Additional file 2. [file 1471-2148-8-191-S14.jpeg]

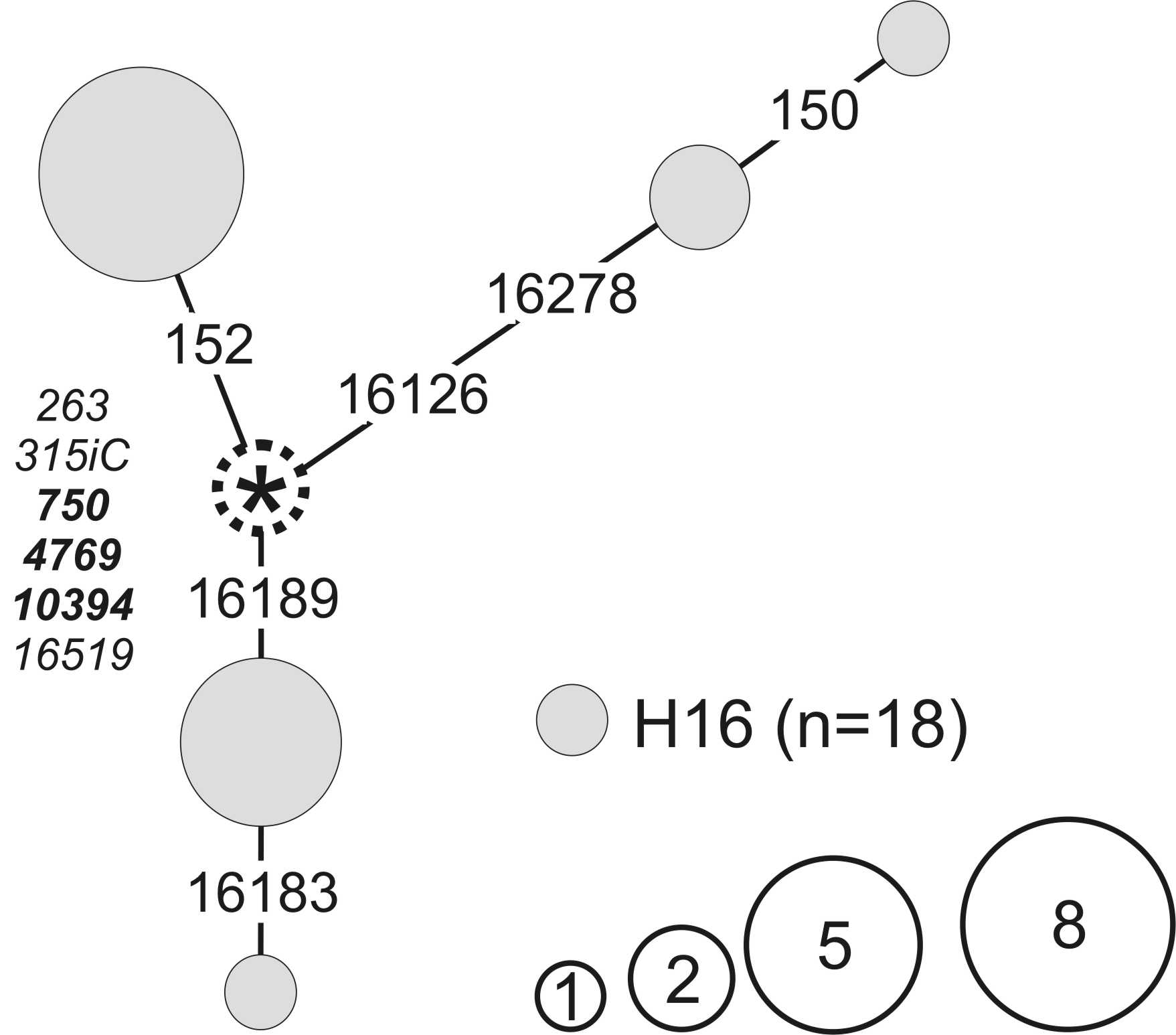

Supplement: Additional file 15 — Median joining network of hg H16. please see Additional file 2. [file 1471-2148-8-191-S15.jpeg]

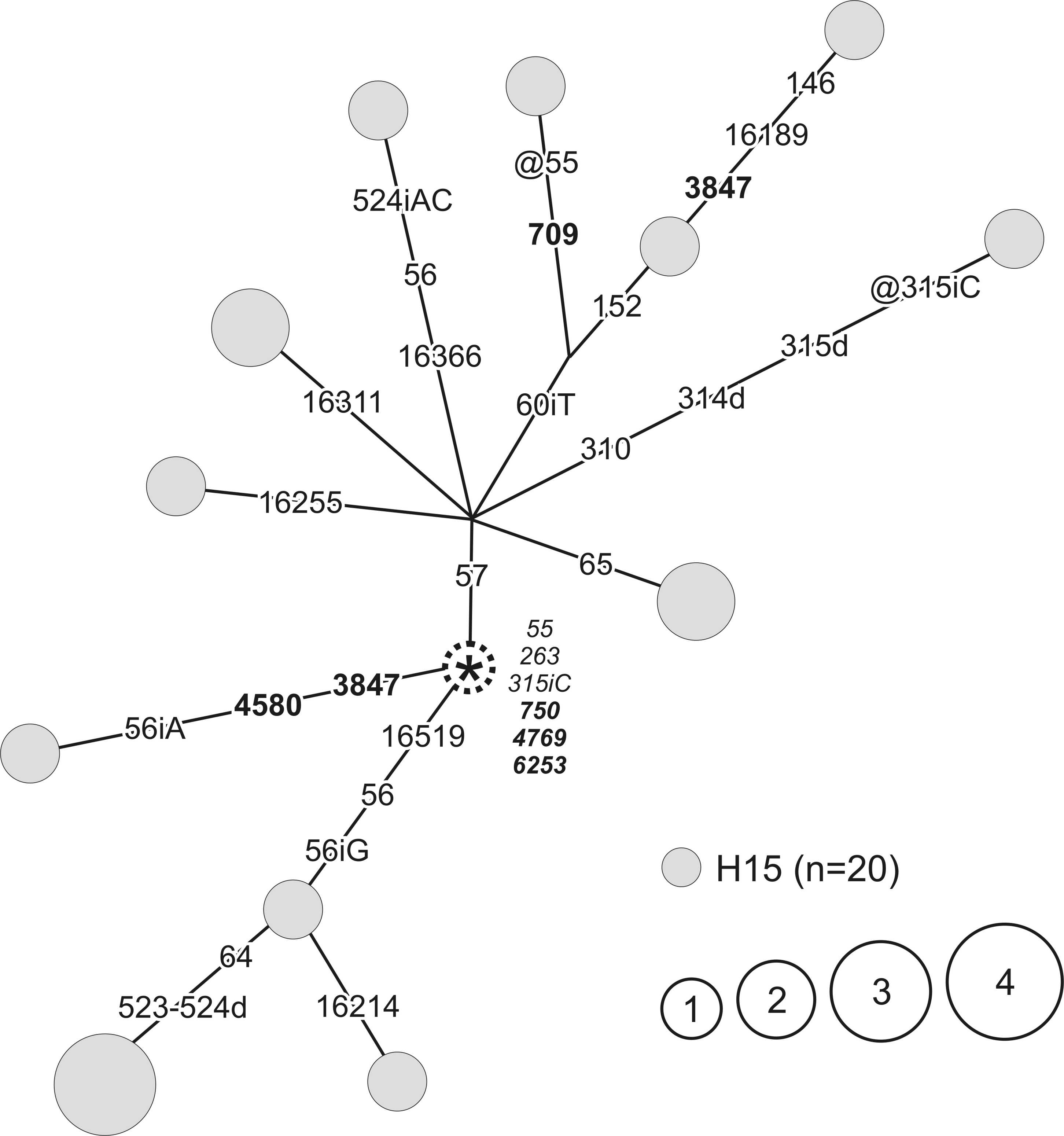

Supplement: Additional file 16 — Median joining network of hg H15. please see Additional file 2. [file 1471-2148-8-191-S16.jpeg]

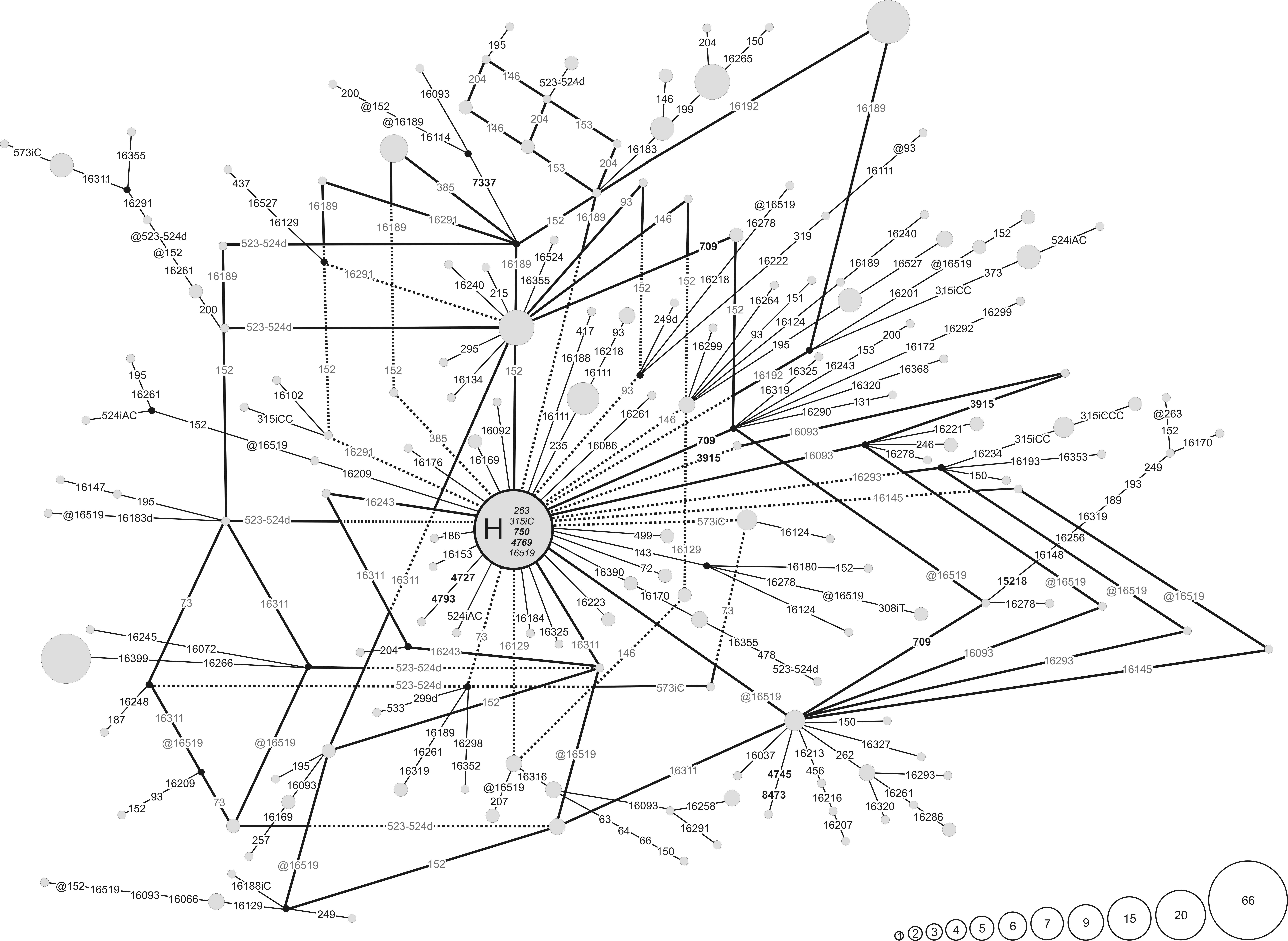

Supplement: Additional file 17 — Median joining network of hg H*. please see Additional file 2. [file 1471-2148-8-191-S17.jpeg]

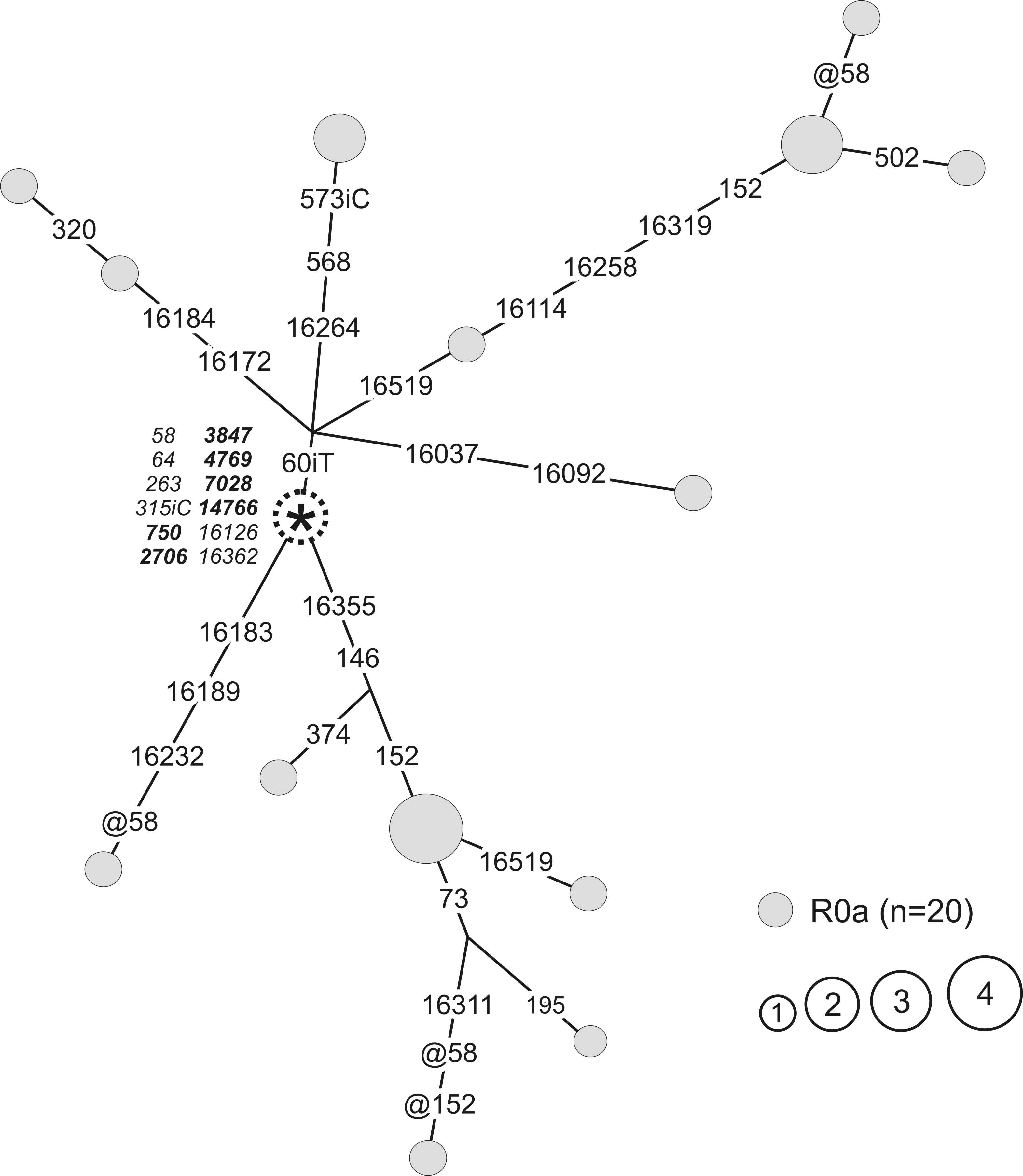

Supplement: Additional file 18 — Median joining network of hg R0a. please see Additional file 2. [file 1471-2148-8-191-S18.jpeg]

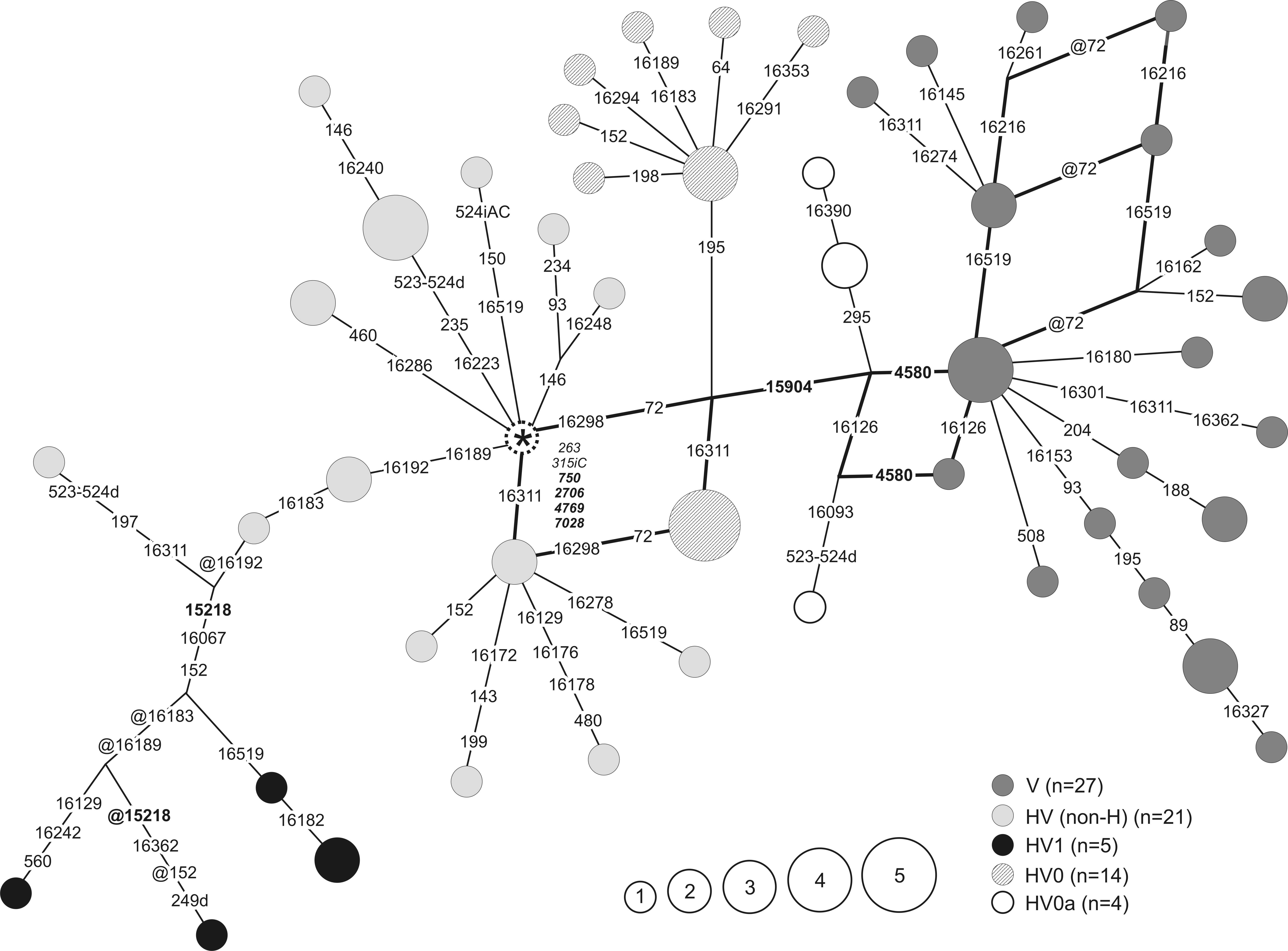

Supplement: Additional file 19 — Median joining network of hg HV*. please see Additional file 2. [file 1471-2148-8-191-S19.jpeg]
